# The amphibians and reptiles of Luzon Island, Philippines, VIII: the herpetofauna of Cagayan and Isabela Provinces, northern Sierra Madre Mountain Range

**DOI:** 10.3897/zookeys.266.3982

**Published:** 2013-02-07

**Authors:** Rafe M. Brown, Cameron D. Siler, Carl. H Oliveros, Luke J. Welton, Ashley Rock, John Swab, Merlijn Van Weerd, Jonah van Beijnen, Edgar Jose, Dominic Rodriguez, Edmund Jose, Arvin C. Diesmos

**Affiliations:** 1 Department of Ecology and Evolutionary Biology and Biodiversity Institute, University of Kansas, Lawrence, Kansas, 66045, USA; 2 Zoology Division, National Museum of the Philippines, Rizal Park, Padre Burgos Avenue, Ermita 1000, Manila, Philippines; 3 Department of Biology, University of South Dakota, Vermillion, South Dakota 57069, USA; 4 Isla Biodiversity Conservation; 9 Bougainvillea Street, Manuela Subdivision, Las Piñas City 1741, Philippines; 5 Institute of Environmental Sciences, Leiden University, PO Box 9518, 2300 RA Leiden, the Netherlands; 6 Mabuwaya Foundation, Isabela State University Campus Garita, Cabagan, Isabela, Philippines; 7 Tagburos centro, Puerto Princesa City, Philippines; 8 Herpetology Section, Zoology Division, Philippine National Museum, Rizal Park, Burgos St., Manila, Philippines

**Keywords:** Biodiversity, Cagayan River Valley, Cordillera Mountain Range, Sierra Madre Mountain Range, Northern Philippines

## Abstract

We provide the first report on the herpetological biodiversity (amphibians and reptiles) of the northern Sierra Madre Mountain Range (Cagayan and Isabela provinces), northeast Luzon Island, Philippines. New data from extensive previously unpublished surveys in the Municipalities of Gonzaga, Gattaran, Lasam, Santa Ana, and Baggao (Cagayan Province), as well as fieldwork in the Municipalities of Cabagan, San Mariano, and Palanan (Isabela Province), combined with all available historical museum records, suggest this region is quite diverse. Our new data indicate that at least 101 species are present (29 amphibians, 30 lizards, 35 snakes, two freshwater turtles, three marine turtles, and two crocodilians) and now represented with well-documented records and/or voucher specimens, confirmed in institutional biodiversity repositories. A high percentage of Philippine endemic species constitute the local fauna (approximately 70%). The results of this and other recent studies signify that the herpetological diversity of the northern Philippines is far more diverse than previously imagined. Thirty-eight percent of our recorded species are associated with unresolved taxonomic issues (suspected new species or species complexes in need of taxonomic partitioning). This suggests that despite past and present efforts to comprehensively characterize the fauna, the herpetological biodiversity of the northern Philippines is still substantially underestimated and warranting of further study.

## Introduction

The highly distinctive terrestrial vertebrate fauna of the northeastern Philippines has been the subject of intense interest, speculation, and debate since the first historical explorations of the northern extremes of the archipelago (Wallace 1860, 1876; Everett 1889; Boulenger 1894; Stejneger 1907; Hoogstral 1951; Allen et al. 2004, 2006). Although many past and recent explorations of this unique part of southeast Asia highlighted spectacular endemic species (Stejneger 1907; Ota and Ross 1994; Brown et al. 2008, 2009; Oliveros et al. 2011), the dominant view of the Philippines by the beginning of the 20^th^ century was the biogeographer’s concept of a “fringing” archipelago (Dickerson 1928; Kloss 1929; Darlington 1957; Myers 1960 1962; Brown and Alcala 1970a; Siler et al. 2012). According to this perception, archipelagos near a continental source for invasion by vertebrate colonists should show distribution patterns consistent with the classic “immigrant pattern” of faunal distributions (Myers 1962; Brown and Alcala 1970a; Lomolino et al. 2010). Thus, early biogeographers expected species to be distributed along possible migration corridors, with various groups extending no further in distance from the continental source, than their relative dispersal abilities would allow (Taylor 1928; Inger 1954; Darlington 1957; Myers 1962; Carlquist 1965; Brown and Alcala 1970a). With respect to the northern Philippines, the most often-cited dispersal corridors included the western island arc (Borneo–Palawan–Mindoro) and the eastern Island chain (Sulu Archipelago–Mindanao–Leyte–Samar; Myers 1962; Esselstyn et al. 2004; Brown and Guttman 2002; Jones and Kennedy 2008; Brown et al. 2009), with more limited evidence in support of southward colonization from Taiwan (Taylor 1928; Kennedy et al. 2000; Esselstyn and Oliveros 2010).

In the context of this biogeographical world view, islands like Luzon, at the tail ends of island chains and possible dispersal routes from the continental source (Diamond and Gilpin 1983; Brown and Guttman 2002; Jones and Kennedy 2008; but see Taylor 1928; Kennedy et al. 2000; Esselstyn and Oliveros 2010) were viewed as the extreme end points of faunal dispersal and dispersion (Huxley 1868; Darlington 1928; Myers 1962; Esselstyn et al. 2004). As a consequence, numerous classic works consider the biodiversity of such islands as “depauperate” in the sense that they contained a reduced set of species shared with a continental mainland source (Dickerson 1928; Taylor 1928; Inger 1954; Brown and Alcala 1970a; Dickinson et al. 1991; de Jong 1996; Lomolino et al. 2010). The view of a depauperate Luzon fauna has persisted throughout the last half century in discussions of its herpetofauna (Inger 1954; Leviton 1963a; Brown and Alcala 1970a, 1978, 1980). Recently however, a renewed interest in faunistic studies of the northern Philippines (Brown et al. 1996, 2000a, 2007, 2012; Diesmos et al. 2005; Siler et al. 2011a) has produced a series of notable discoveries (Alcala et al. 1998, 1999; R. Brown et al. 1999, 2000b, 2008, 2009; W. Brown et al. 1997a, b, c, 1999a, b; Diesmos et al. 2002; Siler et al. 2009, 2010a,b; Linkem et al. 2010; Welton et al. 2010; Fuiten et al. 2011), drawing attention to high levels of species diversity, preponderance of inferred autochthonous speciation, and substantial endemism in the northern reaches of the archipelago (Diesmos et al. 2005; Brown and Diesmos 2009; Brown et al. 2012). Together these studies have suggested that the northern portions of the archipelago may, in fact, be substantially more biologically diverse than currently appreciated. Thus, it is conceivable that, despite past expectations, species richness at a given northern Luzon site may be potentially as high as that demonstrated for the southern portion of the archipelago, adjacent to the Sunda Shelf (Nuñeza et al. 2010; Siler at al. 2011; Diesmos et al. 2005; Diesmos and Brown 2011; Brown et al. 2012).

**Figure 1.**
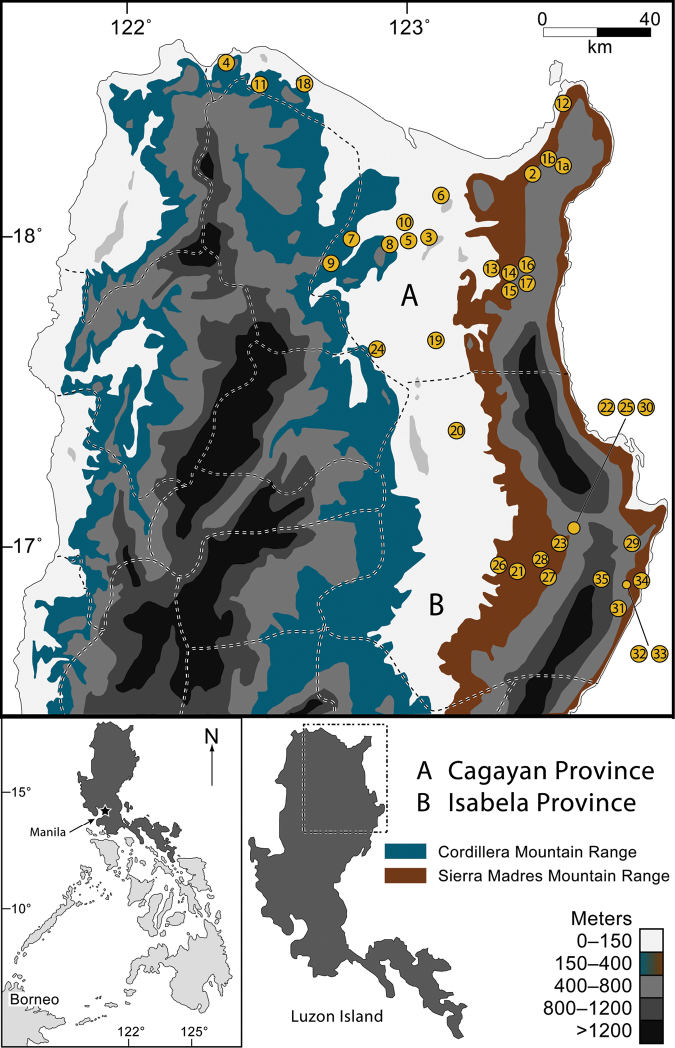
Map of northern Luzon Island, Philippines, with the Sierra Madre and Cordillera mountain ranges indicated (contour shading depicts elevational increments; see key, lower right). Provincial boundaries are indicated with dashed lines. Sampling localities marked with numbered circles, corresponding to localities listed in Table 1. The inset (bottom left) shows the location of Luzon Island (darkly shaded) within the Philippines.

Recent works suggest that the northern end of Luzon Island (Fig. 1) and the islands between Luzon and Taiwan (Oliveros et al. 2011) represent the very last extent of conceivable dispersion of faunal elements of Sundaic origin (Dickerson 1928; Inger 1954, 1999; Inger and Voris 2001). Recent studies have considered the diversity of herpetofaunas of the islands north of Luzon (Oliveros et al. 2011) and the northern end of the Cordillera Mountains of northwest Luzon (Diesmos et al. 2005; Brown et al. 2012).

In this paper we take the first step towards gaining a better understanding of the faunal communities of the northeastern-most extreme of Luzon by considering the amphibians and reptiles of the northern Sierra Madre Mountains (Fig. 1). We provide the first attempt to synthesize the known herpetological diversity of Cagayan and Isabela provinces (Fig. 1), northern Luzon Island (see also van Beijnen 2007, Diesmos 2008). We present data from our own survey work, as well as those from historical museum collections derived from the northern Sierra Madre Mountains. Because very little has been published previously on the herpetological communities of the area, all of these records constitute major range extensions and substantial expansion of our knowledge of resident biodiversity. This work contributes to a growing body of recent literature demonstrating that the herpetological communities of Luzon Island are species rich, composed of high percentages of endemic taxa, and are regionally unique in comparisons to the other zoogeographical regions of Luzon (Auffenberg 1988; Brown et al. 1996, 2000a, 2012; Diesmos et al. 2005; Welton et al. 2010; Siler et al. 2011a; McLeod et al. 2011; Devan-Song and Brown 2012).

**Cagayan and Isabela Provinces: Geography and Landscape.** Cagayan and Isabela provinces lie at the extreme northeastern portion of Luzon (Fig. 1), with land areas totaling more than 9,000 and 10,664 square kilometers, respectively. Cagayan contains 28 municipalities and 825 barangays (villages) while Isabela contains 35 municipalities and 1018 barangays. Their capital cities are Tuguegarao and Ilagan, respectively. Inhabited by six major enthnolinguistic groups (Ilocanos, Ibanags, Malauegs, Itawis, Gaddangs, and Aetas), together they are home to more than 2.6 million human residents (NSO 2010).

**Figure 2.**
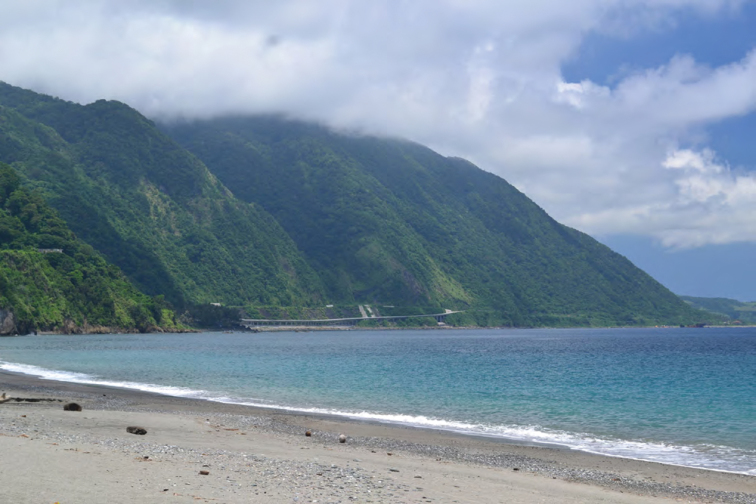
View of the north coast of Luzon, along the boundary between west Cagayan Province and Ilocos Province (fig. 1). Photo: JS.

**Figure 3.**
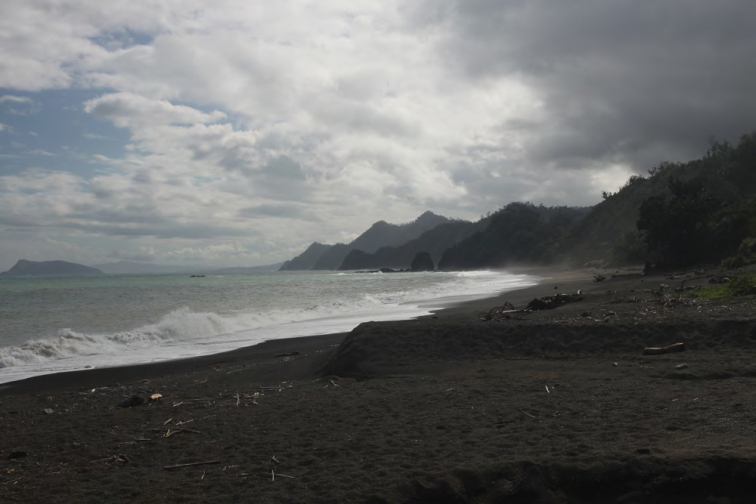
View of the forested west coast of Isabela Province (Dinapique). Photo: MVW.

**Figure 4.**
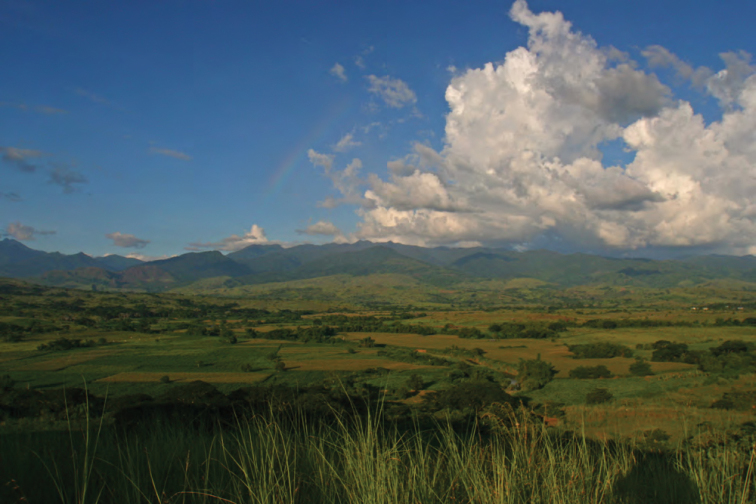
View of the Sierra Madre from the west, at the Municipality of Cabagan, Barangay Garita (Isabela Province). Photo: MVW.

**Figure 5.**
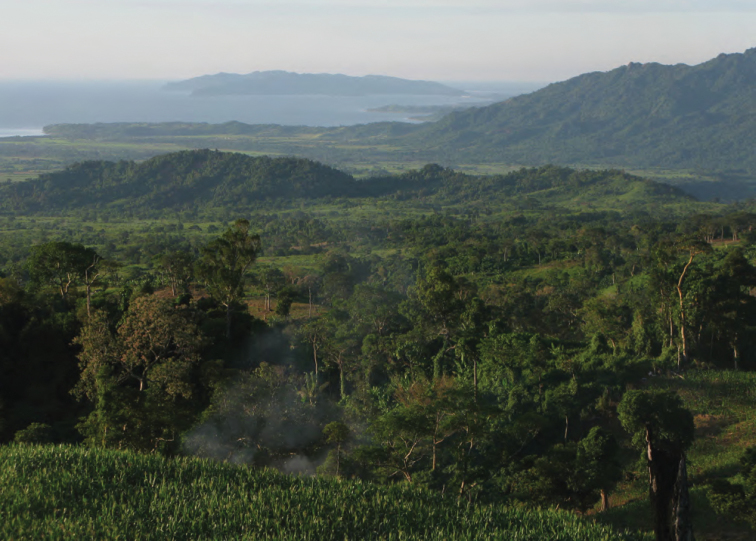
View of the northeast coast of Luzon from the foothills of Mt. Cagua, Municipality of Gonzaga. Note northern end of the Sierra Madre at right and Palaui Island in the background to the right. Photo: RMB.

**Figure 6.**
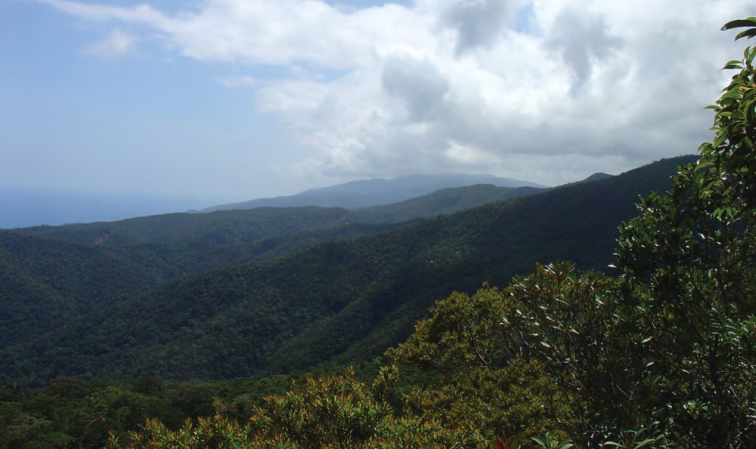
Ultrabasic forests above 1200 m at Barangay Diddadungan, Palanan, northern Isabela Province. Photo: MVW

**Figure 7.**
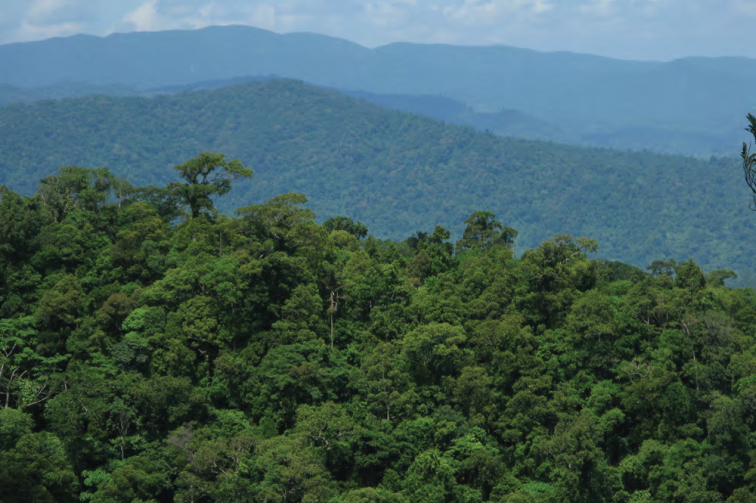
View south of the northern Sierra Madre (from peak of Mt. Cagua, Municipality of Gonzaga, Cagayan Province). Photo: LJW.

**Figure 8.**
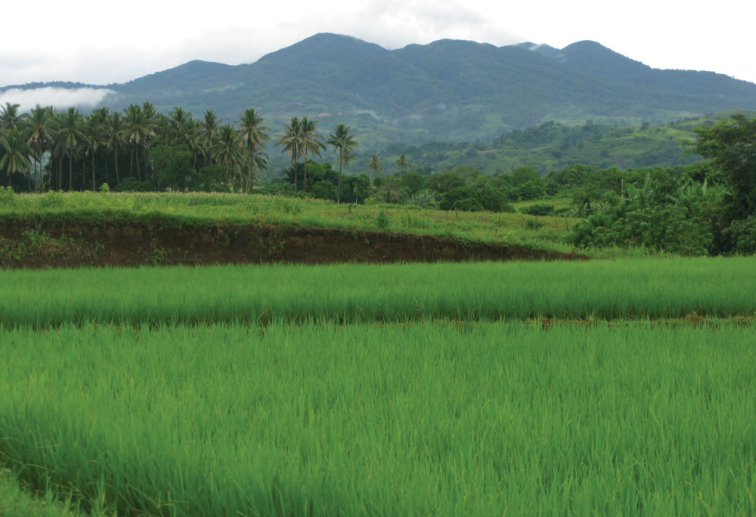
Mt. Cagua, Cagayan Province, with rice fields on the outskirts of Barangay Magrafil in the foreground. Photo: RMB.

Both Provinces are dominated by three strikingly distinct geographical and topographical features: the wide alluvial plains surrounding the Cagayan River valley, the northern extremes of a strikingly elongate north-south mountain range (The Sierra Madre; Figs 3–8), and the narrow strip of coastal forest along the north (Figs 2–3) and the east coasts of northern Luzon and the Philippine Sea (Fig. 1). Portions of the southwestern corner of Cagayan (bordering the province of Kalinga) and all of the western portions of Isabela (bordering Kalinga, Mountain, and Ifugao provinces) abut the foothills of the central Cordillera Mountains of Luzon (Fig. 1). Roughly a third of the land area of these provinces is near sea level; the majority of the remaining area constitutes the mountainous terrain of the northern Sierra Madre Mountain Range and the sprawling foothills to the west and east of this elongate mountain massif.

The Babuyan Island Group across the Balintang channel to the north of Luzon (Fig. 1) is included administratively in Cagayan Province; this biogeographically distinct region has recently been reviewed for its herpetofauna (Oliveros et al. 2011) and will not be treated in detail here.

## Materials and methods

**Figure 9.**
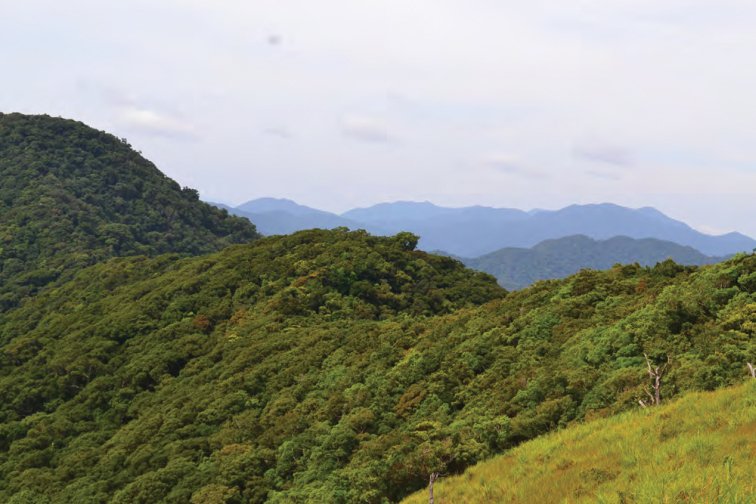
The forested edge of the Mt. Cagua volcanic crater with the northern Sierra Madre in the background. Photo: RMB.

**Figure 10.**
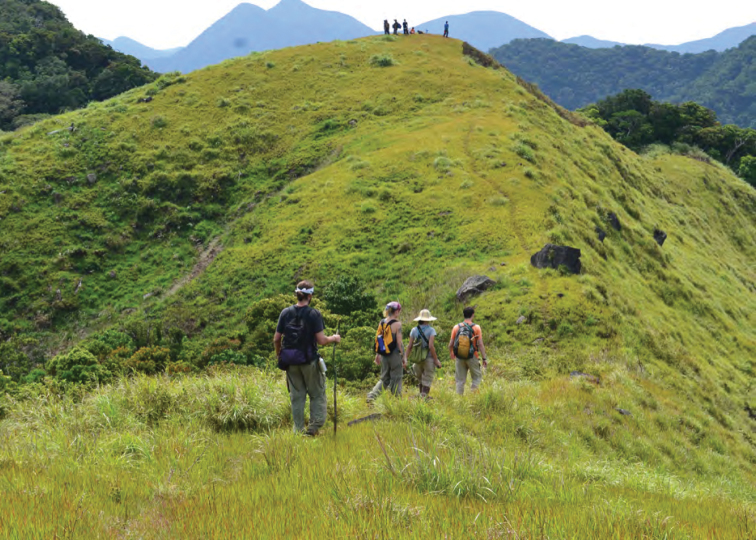
Natural grassland area on the edge of Mt. Cagua volcanic crater. Photo: JS.

**Figure 11.**
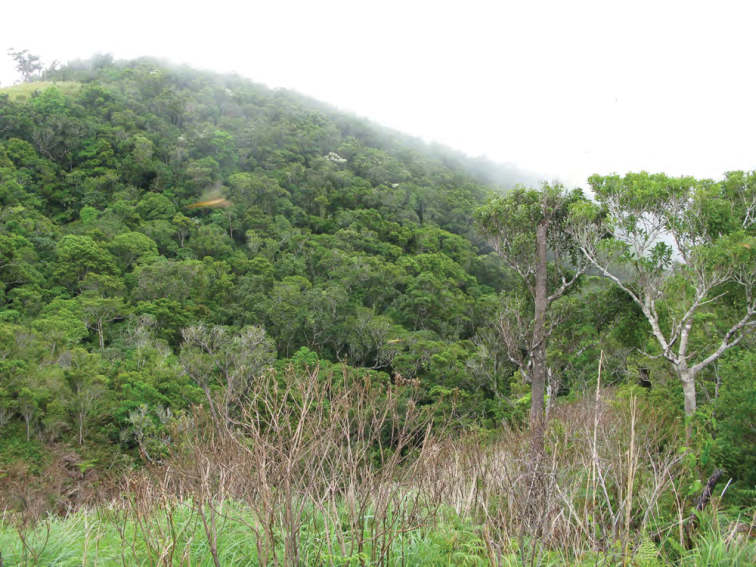
Appearance of lower edge of cloud forest, 1250 m, Mt. Cagua. Photo: RMB.

**Figure 12.**
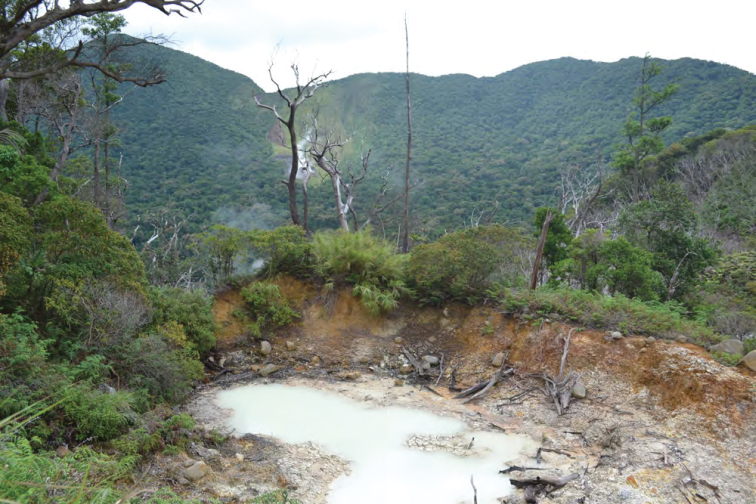
Volcanic vent on the forested floor of the Mt. Cagua crater. Photo: LJW.

**Figure 13.**
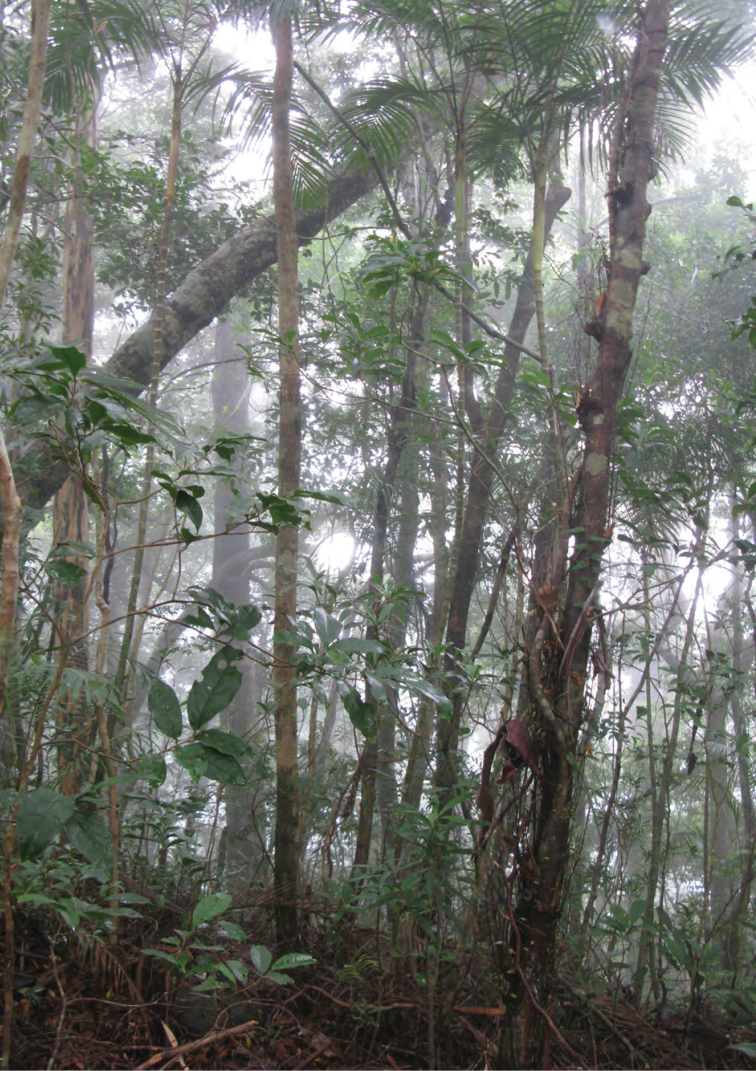
Appearance of Mt. Cagua cloud forest below the canopy at 1250 m asl. Photo: RMB.

**Figure 14.**
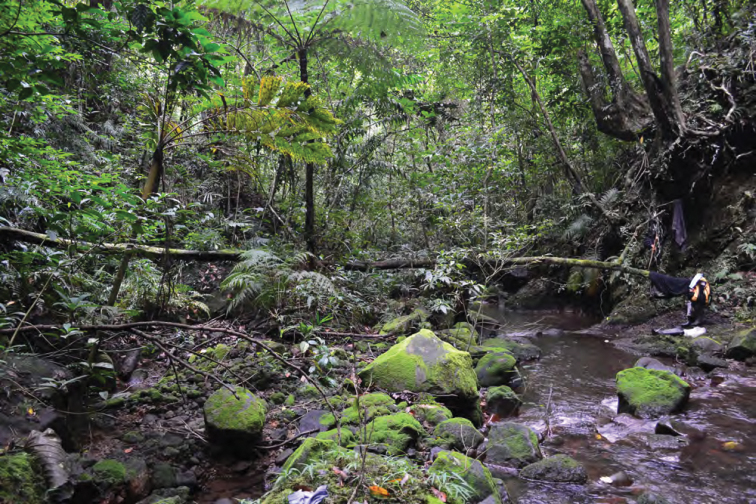
Mature forest at Location 1a in the crater floor of Mt. Cagua. Photo: JS.

**Figure 15.**
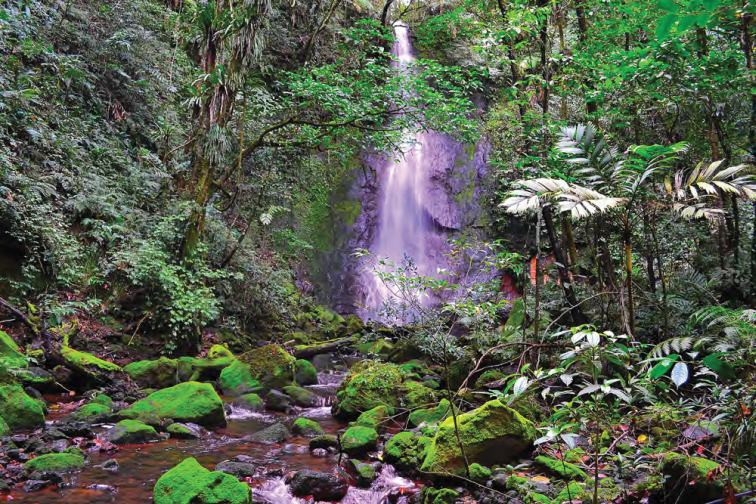
Unnamed waterfall at Location 1a in the crater floor of Mt. Cagua. Photo: JS.

**Figure 16.**
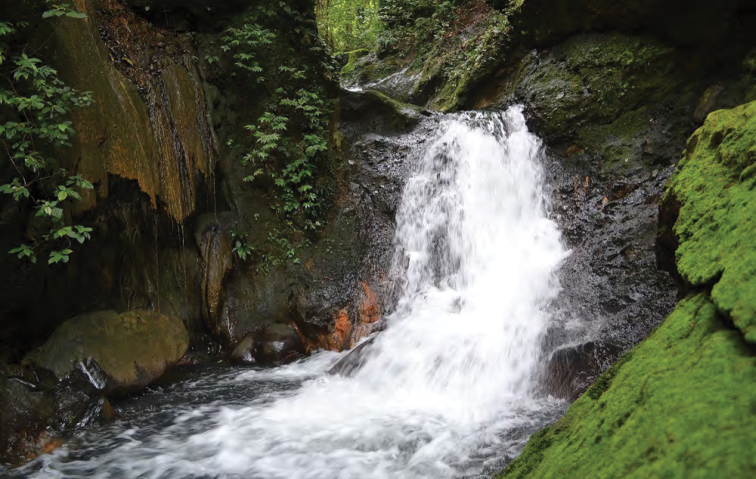
Streamside habitat typical of *Limnonectes macrocephalus*, *Hylarana similis*, *Sanguirana luzonensis*, and *Platymantis* sp. 2 (near Location 1a). Photo: LJW.

**Figure 17.**
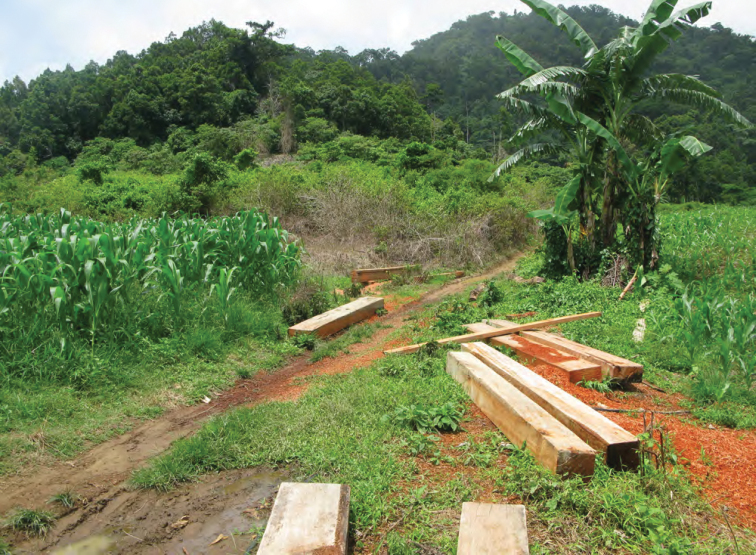
Signs of illegal timber poaching on the boundary of the Mt. Cagua protected area, Location 2. Photo: RMB.

We surveyed amphibian and reptile diversity at numerous sites throughout Cagayan and Isabela provinces (Table 2) using standardized sampling techniques (Heyer et al. 1994) and specimen collection and preservation methodology (Simmons 2002; ASIH 2004). Our most recent surveys (July–August, 2011) involved intensive elevational transects at the extreme northern end of the Sierra Madre Mountain Range in the Mt. Cagua area (Municipality of Gonzaga, Barangays Magrafil and and Santa Clara; Fig. 1). Surveys were conducted in early mornings, mid-day, afternoons, and evenings by experienced teams of four to eight individuals, sampling a wide variety of habitat types within each general study location. Habitats included dry forest on ridges, moist ravines, forest trails at all elevations, dry intermittent streambeds, small streams, large rivers, forest gaps and edges, and grassy open areas (Figs 9–17). Investigators at each sampling location made extensive surveys of each area (on foot) to ascertain habitat types and then visited each at varying times of the day. Nocturnal searches (1800–2400 hr) were conducted at each habitat type, within each sampling site, on dry and rainy nights. By concentrating field survey efforts to span the end of the dry season and the beginning of the rainy season (June–August) we were able to assure that each habitat type at each location was sampled under differing atmospheric conditions.

**Sampling Locations.** Data presented here include results of our own surveys (Table 1) and a variety of collections, both intensive and incidental, from major U.S. Museum collections (see acknowledgements). In addition, an extensive series of collections housed at the USNM (field work of R. I. Crombie), KU and PNM (field work of ACD and surveys of MVW, EJ, and DR) targeted several localities to the south, in central Cagayan Province and Isabela Province. To be as comprehensive as possible in our treatment of Cagayan and Isabela, we include all of these records here, with the caveat that methods of surveying herpetological communities most likely differed among collection efforts and locations.

**Table 1.** Cagayan and Isabela localities where amphibian and reptile specimens have been collected or observed (see Materials and Methods).

**Location**	**Municipality**	**Barangay/Barrio**		**GPS coordinates**
**Cagayan**				
1a	Gonzaga	Barangay Magrafil	Mt. Cagua crater	18.213N, 122.110E
1b	Gonzaga	Barangay Magrafil	Mt. Cagua low elevation	18.236N, 122.104E
2	Gonzaga	Barangay Santa Clara	Purok 7	18.228N, 122.060E
3	Gattaran	Barangay Nassiping		18.054N, 121.641E
4	Santa Praxedes	Taggat Forest Reserve		18.580N, 121.010E
5	Lasam	Lasam Centro		18.051N, 121.600E
6	Lasam	Battalan Barrio		18.171N, 121.723E
7	Lasam	Cabatacan Barrio		18.072N, 121.480E
8.	Lasam	Alannay Barrio		18.053N, 121.551E
9	Lasam	Vintar Barrio		18.045N, 121.43E
10	Lasam	San Pedro		18.081N, 121.597E
11	Santa Ana	Barangay San Vicente,	Sitio Angib	18.490N, 121.168E
12	Santa Ana	Santa Ana Centro		18.482N, 122.1569E
13	Baggao	Barrio Santa Margarita		17.923N, 121.960E
14	Baggao	Road between Barrio San Miguel and Barrio Imurung		17.914N, 121.988E
15	Baggao	Barrio Via	Vicinity of hot springs on bank of Ital river	17.887N, 121.986E
16	Baggao	Barrio San Miguel		17.917N, 122.000E
17	Baggao	Barrio Imurung		17.898N, 122.001E
18	Pamplona	ca. 4 km NW of Abulug River Bridge		18.464N, 121.339E
19	Solana	Barrio Nabbutuan		17.658N, 121.683E
20	Peñablanca	Barangay Malibabag	Callao Caves area	17.677N, 121.8444E
**Isabela**				
21	Cabagan	Barangay Garita, Mitra Ranch		17.417N, 121.8231E
22	San Mariano	Barangay Binatug		16.954N, 122.0669E
23	San Mariano	Barangay Dibuluan, Apaya Creek area	Sitio Apaya	17.029N, 122.1928E
24	San Mariano	Barangay Dibuluan, Dunoy Lake area	Sitio Dunoy	16.995N, 122.1579E
25	San Mariano	Barangay Del Pilar		16.8592N, 122.104E
26	San Mariano	Barangay Dibuluan, Dibanti Ridge, Dibanti River area		17.015N, 122.2036E
27	San Mariano	Barangay Alibadabad		16.964N, 122.0451E
28	San Mariano	San Jose		16.934N, 122.1275E
29	San Mariano	Barangay Disulap		16.963N, 122.1250E
30	Palanan	Barangay Didian	Northern Sierra Madre Natural Park	16.970N, 122.4122E
31	San Mariano	Barangay Dibuluan	Catalangan River	17.024N, 122.1794E
32	Palanan	Barangay Diddadungan	Dyadyadin, ultrabasic forest	16.798N, 122.3922E
33	Palanan	Barangay Diddadungan	Pangden, lowland dipterocarp forest	16.833N, 122.4181E
34	Palanan	Barangay Diddadungan	Limestone forest near Magsinaraw cave	16.941N, 122.4536E
35	Palanan	Barangay Diddadungan	Coastal habitat, Divinisa	16.834N, 122.4319E
36	Palanan	Barangay Didian	Dipagsanghan, lowland dipterocarp forest	16.879N, 122.3447E
37	Maconacon	Barangay Reina Mercedes	Blos River	17.508N, 122.1916E

**Table 2.** Amphibians (anurans) and reptiles (lizards , snakes, turtles, and crocodiles) from Cagayan Province, andIsabela Province (to the south; together the two provinces make up the northern Sierra Madre Mountain Range, of Luzon Island; Fig. 1). N = new provincial record (observed during this study, with voucher specimen). P = previously reported literature records for Cagayan or Isabela provinces (with vouchered specimens of photographic evidence deposited in museum collections). O = new observation (no voucher specimens collected). R = Range extension within Cagayan and/or Isabela Province. * = Luzon faunal region (Brown and Diesmos 2002, 2009) endemics; n= 46.

**AMPHIBIA**	**Cagayan**	**Isabela**
Bufonidae		
*Rhinella marina* (Linnaeus, 1758)	N	P
Ceratobatrachidae		
*Platymantis cagayanensis* Brown, Alcala & Diesmos, 1999*	P, R	N
*Platymantis corrugatus* (Duméril, 1853)	N	N
*Platymantis cornutus* (Taylor, 1922)*	N	N
*Platymantis polillensis* (Taylor, 1922)*	N	
*Platymantis pygmaeus* Alcala, Brown & Diesmos, 1998*	N	P
*Platymantis sierramadrensis* Brown, Alcala, Ong & Diesmos, 1999*	N	P
*Platymantis taylori* Brown, Alcala & Diesmos, 1999*		P
*Platymantis* sp. “Yokyok” *	N	N
*Platymantis* sp.2 “Cheep-cheep” *	N	N
*Platymantis* sp.3 “See-yok” *	N	N
*Platymantis* sp.	N	N
Dicroglossidae		
*Fejervarya moodiei* (Taylor, 1920)	N	
*Fejervarya vittigera* (Wiegmann, 1834)	N	N
*Hoplobatrachus rugulosu*s (Wiegmann, 1834)	N	N
*Limnonectes macrocephalus* (Inger, 1954)*	N	P
*Limnonectes woodworthi* (Taylor, 1923)*	N	N
*Occidozyga laevis* (Günther, 1859)	P	N
Microhylidae		
*Kaloula kalingensis* Taylor, 1922*	N	P
*Kaloula rigida* Taylor, 1922*	N	N
*Kaloula picta* (Duméril & Bibron, 1841)	N	N
*Kaloula pulchra* Gray, 1825	N	N
Ranidae		
*Hylarana similis* (Günther, 1873)*	N	P
*Sanguirana luzonensis* (Boulenger, 1896)*	N	P
*Sanguirana tipanan* (Brown, McGuire & Diesmos, 2000)*	P	
Rhacophoridae		
*Philautus surdus* (Peters, 1863)	N	N
*Polypedates leucomystax* Gravenhorst, 1829	N	N
*Rhacophorus pardalis* Günther, 1859	N	N
*Rhacophorus appendiculatus* (Günther, 1858)	N	
REPTILIA (Lizards)		
Agamidae		
*Bronchocela marmorata* Gray, 1845	N	N
*Draco spilopterus* (Wiegmann, 1834)	N, P	N
Gekkonidae		
*Cyrtodactylus philippinicus* (Steindacher, 1867)	N	N
*Gehyra mutilata* (Wiegmann, 1834)	O	N
*Gekko gecko* (Linnaeus, 1758)	N	N
*Gekko kikuchii* (Oshima, 1912)	N	N
*Hemidactylus frenatus* Duméril & Bibron, 1836	O	N
*Hemidactylus platyurus* (Schneider, 1792)	O	
*Hemidactylus stejnegeri* Ota & Hikida, 1989	N	
*Lepidodactylus* cf. *lugubris* (Duméril & Bibron, 1836)	N	N
*Luperosaurus* cf. *kubli* Brown, Diemsos & Duya, 2007*	N	N
*Pseudogekko compressicorpus* (Taylor, 1915)	N	
Scincidae		
*Brachymeles bicolor* (Gray, 1845)*	N	P
*Brachymeles bonitae* Duméril & Bibron, 1839	N	N
*Brachymeles kadwa* Siler & Brown, 2010*		P
*Brachymeles muntingkamay* Siler, Rico, Duya & Brown, 2009*	N	
*Eutropis cumingi* (Brown & Alcala, 1980)	N	N
*Eutropis multicarinata borealis* Brown & Alcala, 1980	N	N
*Eutropis multifasciata* (Kuhl, 1820)	O	N
*Lamprolepis smaragdina philippinica* Mertens, 1829	N	N
*Lipinia* cf. *vulcania* Girard, 1857		N
*Otosaurus cumingi* Gray, 1845	N	N
*Pinoyscincus abdictus aquilonius* (Brown & Alcala, 1980)	N	N
*Parvoscincus decipiens* (Boulenger, 1895)*	N	N
*Parvoscincus* cf. decipiens*	N	N
*Parvoscincus leucospilos* (Peters, 1872)*	O	N
*Parvoscincus steerei* (Stejneger, 1908)	N	N
*Parvoscincus tagapayao* (Brown, McGuire, Ferner & Alcala, 1999)*	N	N
Varanidae		
*Varanus marmoratus* (Wiegmann, 1834)*	N	P
*Varanus bitatawa* Welton, Siler, Bennet, Diesmos, Duya, Dugay, Rico, Van Weerd & Brown, 2010*	P, R	P
REPTILIA (Snakes)		
Colubridae		
*Ahaetulla prasina preocularis* (Taylor, 1922)	N	N
*Boiga cynodon* (Boie, 1827)	N	N
*Boiga dendrophila divergens* Taylor, 1922*	N	
*Boiga philippina* (Peters, 1867)*	N	
*Calamaria bitorques* Peters, 1872*	N	N
*Calamaria gervaisi* Duméril & Bibron, 1854	N	N
*Coelognathus erythrurus manillensis* (Jan, 1863)*	N	P
*Cyclocorus lineatus lineatus* (Reinhardt, 1843)*	N	N
*Dendrelaphis luzonensis* Leviton, 1961*	N	N
*Dendrelaphis marenae* Vogel & van Rooijen, 2008	N	N
*Dryophiops philippina* Boulenger, 1896	N	N
*Gonyosoma oxycephalum* (Boie, 1827)	N	N
*Hologerrhum philippinum* Günther, 1858*	N	N
*Lycodon capucinus* (Boie, 1827)	N	N
*Lycodon muelleri* Duméril, Bibron & Duméril, 1854*		N
*Lycodon solivagus* Ota & Ross, 1984*		N
*Oligodon ancorus* (Girard, 1858) *		N
*Psammodynastes pulverulentus* (Boie, 1827)	N	N
*Pseudorhabdion* cf. *mcnamarae* (Taylor, 1917)*		N
*Pseudorhabdion* cf. *talonuran* Brown, Leviton & Sison, 1999*		N
*Ptyas luzonensis* (Günther, 1873)	N	
*Rhabdophis spilogaster* (Boie, 1827)	N	N
*Tropidonophis dendrophiops* (Günther, 1883)	N	N
Elapidae		
*Hemibungarus calligaster* (Wiegmann, 1835)*		N
*Naja philippinensis* Taylor, 1922; *Ophiophagus hannah* (Cantor, 1836)		NN
Homolopsidae		
*Cerberus schneideri* (Schlegel, 1837)		N
Lamprophiidae		
*Oxyrhabdium leporinum leporinum* (Günther, 1858)*	N	
Pythonidae		
*Python reticulatus* (Schneider, 1801)	N	P
Typhlopidae		
*Ramphotyphlops braminus* (Daudin, 1803)	N	N
*Typhlops ruficaudus* (Gray, 1845)*		N
*Typhlops* sp. 1*		N
*Typhlops* sp. 2*		N
Viperidae		
*Trimereserus flavomaculatus* (Gray, 1842)	N	N
*Tropidolaemus subannulatus* (Gray, 1842)		O
		
REPTILIA (Turtles)		
Geomydidae		
*Cuora amboinensis amboinensis* (Daudin, 1802)	P	N
Trionychidae	P	P,O
*Pelochelys cantorii* Gray, 11864	P	P,O
Cheloniidae		
*Caretta caretta* (Linnaaeus, 1758)		O
*Chelonia mydas* (Linnaaeus, 1758)		O
*Eretmochelys imbricata* (Linnaaeus, 1766)		O
Crocodylidae		
*Crocodylus mindorensis* Schmidt, 1935	P,O	P,O
*Crocodylus porosus* Schneider, 1801	P,O	P,O

## Results

We document 101 species of amphibians and reptiles from Cagayan and Isabela provinces, including 29 frog species, 30 lizards, 35 snakes, two freshwater turtles, three marine turtles, and two crocodilian taxa (Table 2). Taken together this diversity represents approximately 35% percent of the total Philippine herpetofauna (approximately 350 species; Brown 2007; Brown et al. 2008; Diesmos et al. 2002; Diesmos and Brown 2011; Brown and Stuart 2012) and 70% of the taxa recorded are Philippine endemics. Below we provide accounts for each species, provide notes on their natural history and habitat, and highlight many unresolved taxonomic problems (involving 38% of the species included) that are relevant to particular taxa. We also comment on the conservation status of individual species when data presented here suggest that existing conservation status assessments (IUCN 2010, 2011) are out of date (Siler et al. 2011; McLeod et al. 2011; Brown et al. 2012) or will soon require revision.

## Species accounts

### Amphibia. Family Bufonidae

***Rhinella marina* (Linnaeus, 1758)**

**Figure 18.**
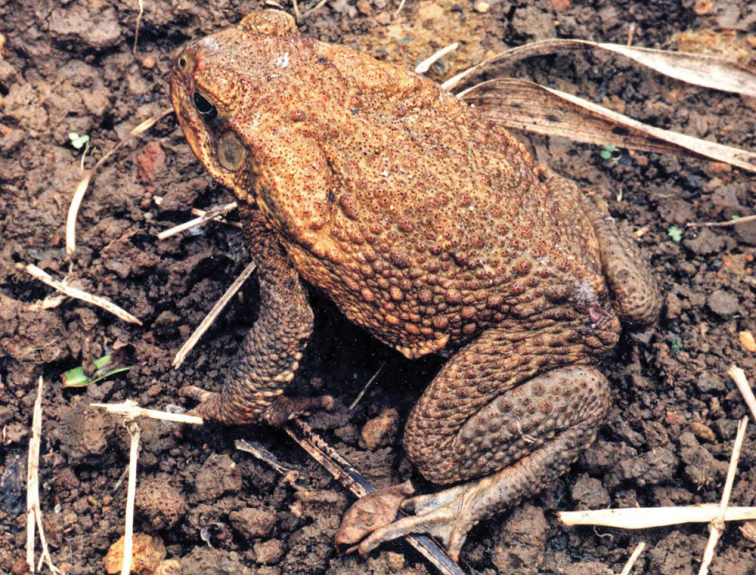
*Rhinella marina* at San Mariano (Location 23; specimen not collected); Photo: ACD.

*Rhinella marina* (Fig. 18) is a non-native species that may have originally been introduced to the Philippines during the industrial revolution and the major sugar cane agricultural production boom on the central Philippine island of Negros (Brown and Alcala 1970a; Alcala and Brown 1998; Diesmos et al. 2006). Since its introduction it has spread widely throughout the country and has been found throughout low elevation agricultural areas where densities may be particularly high (Alcala 1957; Afuang 1994), in foot hills of major mountains, and even as high as 1200 masl in selected areas (RMB, CDS, ACD, *personal observation*). Our specimens (only a few collected among the many encountered) were located on trails in selectively logged forests, at mid elevations, and near forest edges and shifting agricultural plantations.

Cagayan Province—Location 1b: KU 330585; Location 5: USNM 498512–15, PNM 7424.

Isabela Province—Location 21: KU 307440.

### Family Ceratobatrachidae

***Platymantis cagayanensis* Brown, Alcala & Diesmos, 1999**

**Figure 19.**
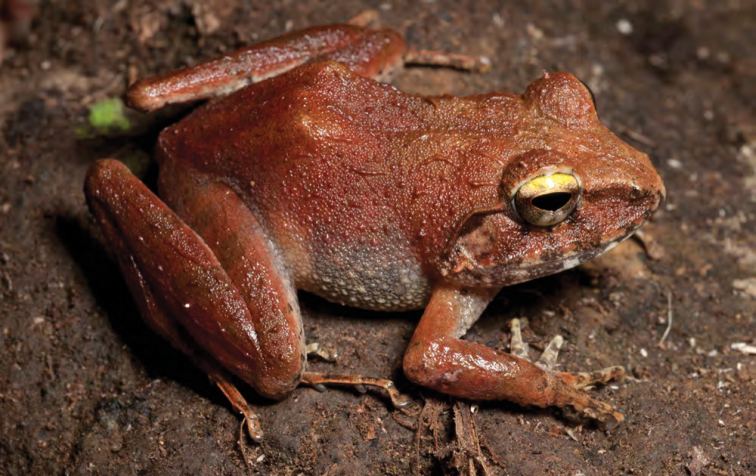
*Platymantis cagayanensis* (KU 330716) from mid-elevation of Mt. Cagua (Location 1b). Note diagnostic yellow coloration of upper iris. Photo: RMB.

Originally described from extreme northwest Cagayan Province at the Municipality of Santa Praxedes along the border with Ilocos Norte Province (Brown et al. 1999b), this species is now known to be widespread throughout the north coast of Luzon, including the northern ends of the Cordillera and Sierra Madre Mountain ranges (Alcala and Brown 1998, 1999; Brown et al. 2012). The identification of this species in the field is complicated by its variable coloration, tuberculate dorsal surfaces, and moderate body size, a suite of characters it shares with many frogs in the *Platymantis dorsalis* species group (Alcala and Brown 1998, 1999). However, this species may be reliably diagnosed in life from its sympatric congeners *Platymantis* sp. “seeyok” and *Platymantis* sp. “yok-yok” (see below) by its bright yellow or yellow-orange iris color above the pupil (Fig. 19) and its distinct advertisement calls, sounding to the human ear like “Eeeerrr-root” or “Kreeee-eek” (Brown et al. 1999b; *personal observation*). Our observation of this species as locally abundant, widespread, and commonly encountered in northern Cagayan Province supports Brown et al.’s (2012) downgrading of its conservation status from “Vulnerable” to “Near Threatened” (IUCN 2010, 2011).

Cagayan Province—Location 1a: KU 330300–01; Location 1b: KU 330302–26; Location 3: PNM 7614–24; Location 4: CAS 207447–50 (paratypes); PNM 6691 (holotype), 6692–93 (paratypes).

Isabela Province—Location 36: no specimens (MVW photo voucher).

***Platymantis corrugatus* (Duméril, 1853)**

**Figure 20.**
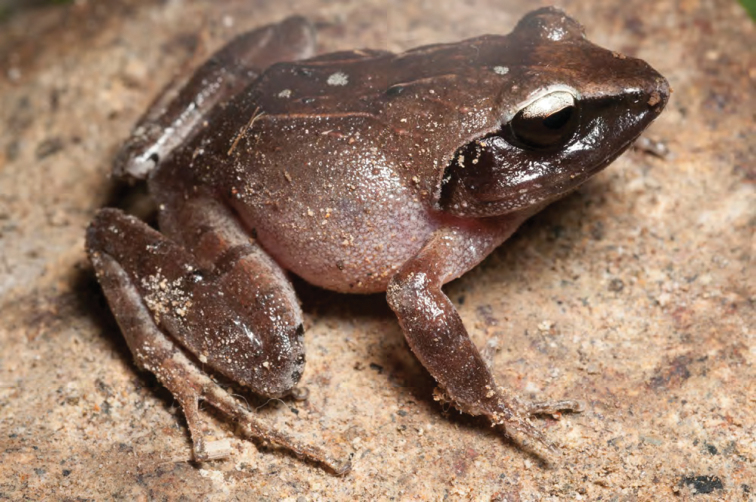
*Platymantis corrugatus* (KU 330255) from mid-elevation of Mt. Cagua (Location 1b) Location 1b. Photo: RMB.

*Platymantis corrugatus* (Fig. 20), as presently recognized, is a widespread endemic species found throughout the archipelago. There is considerable color pattern variation, but the species can be generally diagnosed by its medium body size, some form of a dark (gray, brown, or black) facial mask, and elongate tubercular ridges running along the dorsal surface. We observed this species at many locations calling most intensively at sunset (1800–1900 hr) after which it only called intermittently. The species commonly calls from beneath some kind of ground cover (leaf of other debris) on the forest floor. Its call sounds to the human ear like a raspy “whaaah…whaaah.”

Cagayan Province—Location 1a: KU 330249–54: Location 1b: KU 330255–63; Location 11: PNM 6453; Location 15: USNM 498730.

Isabela Province—Location 30: PNM 6448, MVW photo voucher.

***Platymantis cornutus* (Taylor 1922)**

**Figure 21.**
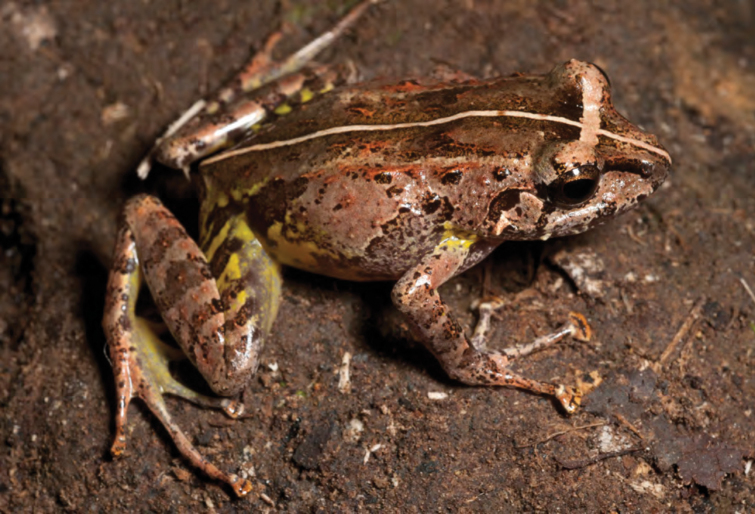
*Platymantis cornutus* (KU 330390) from mid-elevation of Mt. Cagua (Location 1b). Note diagnostic yellow inguinal coloration and white infratympanic tubercle. Photo: RMB.

Originally described on the basis of a single specimen from Balbalan, Kalinga, in the northern Cordillera Mountain Range (holotype CAS 231501; Taylor 1920, 1922a), this species (Fig. 21) is widespread, commonly encountered, and locally abundant (given sufficient precipitation) at mid- to high-elevation sites in the Sierra Madre Range (Brown et al. 2000a; 2012; Diesmos et al. 2005; Siler et al. 2011a). We have no reliable records of any other member of the *Platymantis guentheri* Group (Brown et al. 1997b, 1997c) of frogs at the same localities where *Platymantis cornutus* has been recorded in the mountains of extreme northern Luzon, rendering our confidence in this identification very high. *Platymantis cornutus* calls from understory vegetation immediately following rain and is most frequently encountered on axils and along fronds of aerial ferns. This species deposits direct-developing embryos in small clutches (6–8 eggs) on fern axils (Brown et al. 2012). It has one of the most rapid advertisement calls of any Philippine *Platymantis*, sounding to the human ear like “Tuk-tuk-tuk-tuk…” with 10–20 rapidly-delivered individual pulses. Geographic records reported here contribute to the continued expansion of this species’ range throughout much of northern Luzon, supporting Brown et al.’s (2012) action of downgrading this species from “Vulnerable” (VU) to “Near Threatened” (IUCN 2011).

Cagayan Province—Location 1a: KU 330362–89; Location 1b: KU 330390–92.

***Platymantis polillensis* (Taylor 1922)**

**Figure 22.**
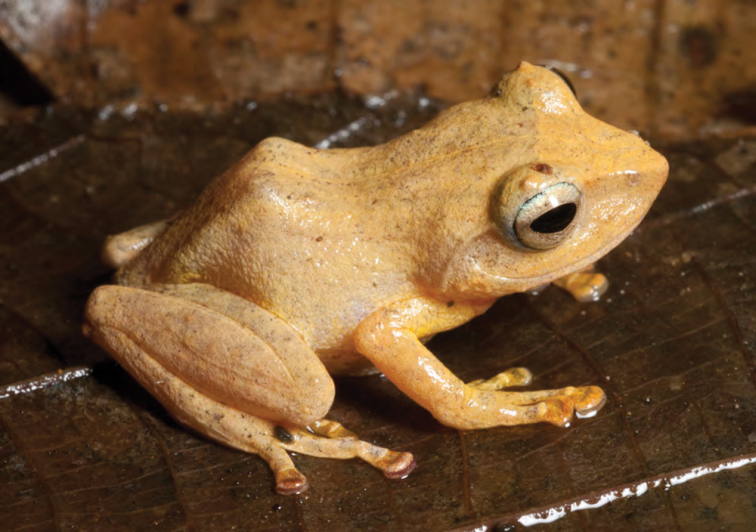
Female *Platymantis polillensis* (KU 330235) from mid elevation of Mt. Cagua (Location 1b). Photo: RMB.

*Platymantis polillensis* (Fig. 22) is a small, herbaceous-layer specializing arboreal species encountered most often in ferns and shrubs colonizing disturbed forest edges, secondary growth forest, forest gaps, and tree falls. Previously considered “Critically Endangered,” or “Endangered” (IUCN 2011) and endemic to the island of Polillo (Quezon Province, off the coast of SE Luzon; holotype CAS 62250), this species is now known to be widespread, commonly encountered (given occurrence of precipitation and preferred habitat type), and often locally abundant (Brown et al. 2000a, 2012; Siler et al. 2011a; McLeod et al. 2011). The major range extension reported here supports Brown et al.’s (2012) downgrading of this species conservation status to “Near Threatened” (IUCN 2010) based on its additional presence in Aurora Province, southern Luzon. This species calls with a slow series of amplitude-modulated high frequency “chirps” following sufficient precipitation.

Cagayan Province—Location 1a: KU 330234–35; Location 1b: KU 330236–38.

Isabela Province—Location 33: no specimens (MVW photo voucher).

***Platymantis pygmaeus* Alcala, Brown & Diesmos, 1998**

**Figure 23.**
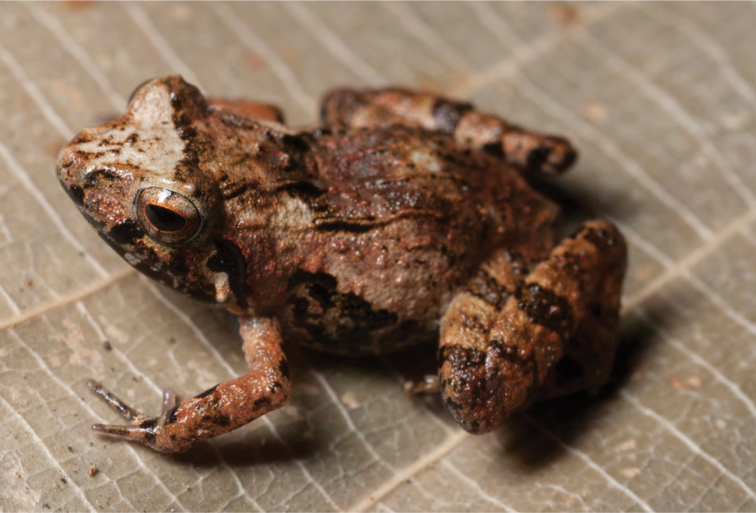
*Platymantis pygmaeus* (specimen not collected) at San Mariano (Location 33). Photo: MVW.

*Platymantis pygmaeus* (Fig. 23) was originally described from Palanan, Isabela Province, and is now known to be widespread and abundant in Bulacan, Quezon, Aurora, Kalinga, Isabela, Cagayan, and Ilocos Norte provinces (Alcala et al. 1998; Brown 2000b; Siler 2010; McLeod et al. 2011). The substantial distributional record reported here, while not surprising, constitutes additional evidence in support of Brown et al.’s (2012) downgrading of this species conservation status from “Vulnerable” (IUCN 2011) to “Near Threatened” (IUCN 2010). This is the smallest species of *Platymantis* in the Philippines (male SVL 12–15 mm) and it can be recognized in life by its high frequency “click-click-click…” advertisement call and its preference for calling from low (0.3–1.0 m), shrub layer vegetation.

Cagayan Province—Location 1a: KU 330239–43; Location 1b: KU 330244–48; Location 13: PNM 7800–01; Location 33: no specimen (MVW photo voucher).

Isabela Province—Location 30: CAS 204762–66 (paratypes), PNM 6255 (holotype), 7792–99; Location 33: no specimens (MVW photo voucher).

***Platymantis sierramadrensis* Brown, Alcaka, Ong & Diesmos, 1999**

*Platymantis sierramadrensis* was described on the basis of specimens from Barangay Umiray, Municipality of General Nakar, Quezon Province (holotype PNM 6465), from Aurora Province (paratypes 204738, 204742–45), and other, non-type material from Palanan, Isabela Province (CAS 204739, 204740, CAS 204741). Subsequent confusion in identification of *Platymantis sierramadrensis* has involved a suspicion that two separate taxa may have been attributed to this species, a confusion that may have undermined the type description (Brown et al. 1999; Brown et al. 2000a, Siler et al. 2011a). Since the realization of this potential problem, we have twice noted (Brown et al. 2000a; Siler et al. 2011a) the presence of two sympatric small bodied *Platymantis hazelae* Group (Brown et al. 1997b) species, one of which appears to be most abundant at lower elevations (approximately 400–700 m) in disturbed habitats and another that is often encountered at the upper end of this elevational range, but is most abundant at elevations above 900 m. We consider the lower elevation species, with a “chirp” mating call, to be the widespread, common species *Platymantis polillensis*,and the slightly larger bodied, high elevation species, tentatively assigned to *Platymantis sierramadrensis*. The latter calls with a pure, constant frequency call, sounding to the human ear like the ringing of a small bell (thus differing from the “chirp” call of *Platymantis polillensis*). Current IUCN conservation classification for this species is “Vulnerable (B1ab(iii)),” based on our assessment from 2004 (IUCN 2011). Considering the taxonomic confusion still surrounding this species, the lack of reliable past records, and the absence of any convincing evidence of population, area of occurrence, or habitat decline, we now consider this species to be “Data Deficient (DD; IUCN 2010, 2011). Once the taxonomy of this species is clarified with a return to the type locality in General Nakar to determine which call type occurs there, direct, field-based data gathered from natural populations (and not inferred from forest cover) will be necessary to reconsider a higher possible conservation threat level (IUCN 2011).

Cagayan Province—Location 1a: KU 330637–51.

Isabela Province—Location 30: CAS 204739–41; Location 36: PNM 6461–63, 6470–74.

***Platymantis taylori* Brown, Alcala, Diesmos, 1999**

**Figure 24.**
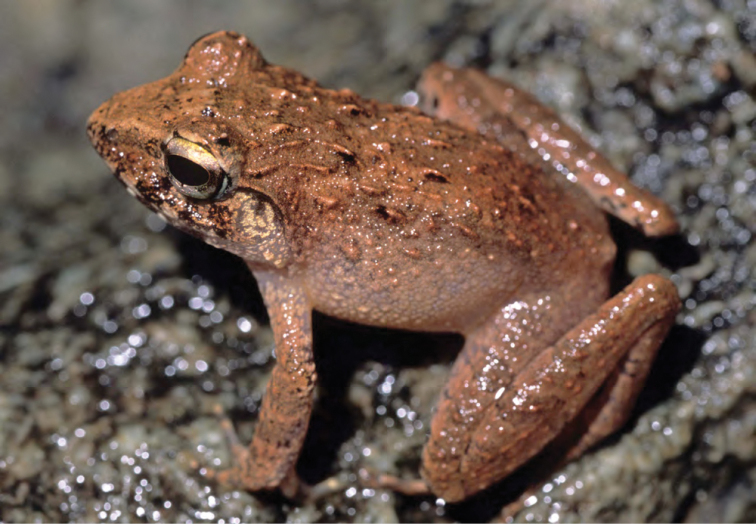
*Platymantis taylori* (PNM 8676) from San Mariano (Location 29). Photo: ACD.

Since the time of its discovery (Brown et al. 1999), this species (Fig. 24) has been documented only at the Municipality of Palanan (Barangay Didian). This taxon was diagnosed primarily on the basis of its relatively large body size and distinctive advertisement call, sounding to the human ear like the buzz produced by a Geiger counter. This species previously has been classified by IUCN as “Endangered” (EN; B1ab(iii); IUCN 2011, 2011), on the basis of its purported limited range and anticipated decline in habitat due to the presence of logging at low elevations along Luzon’s east coast near Palanan.

Long overdue for a conservation status revision, we categorize this species as “Data Deficient” (DD) because (1) it has been recorded only once and no repeat surveys to the immediate or surrounding areas have been undertaken to determine the extent of its range, and (2) there is no evidence that this taxon requires intact, low-elevation forest and no evidence to suggest that it is range-restricted. Thus, there is no way to determine whether continued degradation of lowland coastal forests in Palanan will adversely affect this species. Originally characterized as “common and widespread” at the original collection site (Brown et al. 1999; IUCN 2004), its range presumably includes an extremely large protected area, supporting our conviction that this species must be downgraded to a low conservation threat category (e.g., “Near Threatened,” NT) or, more appropriately, considered “Data Deficient” until some attempt is made to study it in the field and more surveys in surrounding areas are conducted. *Platymantis taylori* is another example of a case in which negative data have been used inappropriately for conservation status assessment (Brown et al. 2012), resulting in a higher level of threat category when, in reality, virtually nothing is known of its biology, natural history, habitat requirements, and actual conservation status.

Isabela Province—Location 21: PNM 8676; Location 26: ACD specimens deposited in PNM; Location 30: CAS 207440–46 (paratypes), PNM 6684, 8659–74, 8953.

***Platymantis* sp. 1 “Yokyok”**

**Figure 25.**
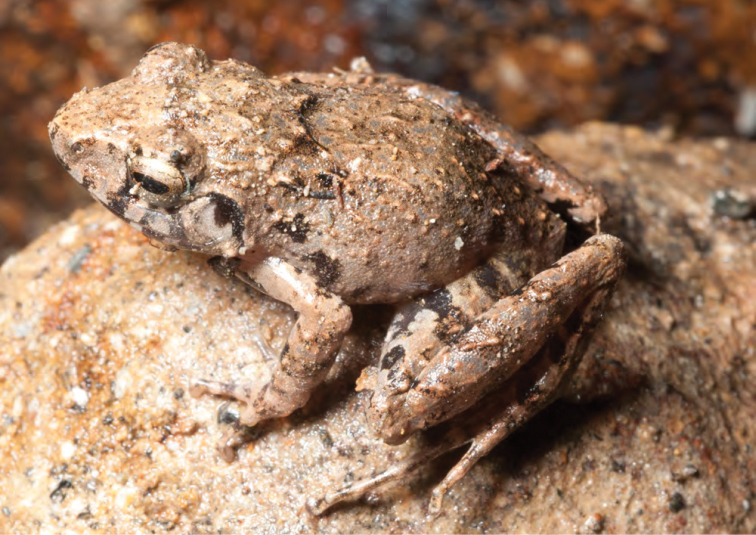
*Platymantis* sp. 1 (“Yokyok;” KU 330628) from lower elevation Mt. Cagua, Municipality of Gonzaga, below Location 1b. Photo: RMB.

This distinctive form (Fig. 25) is now known from two sites in Cagayan and Isabela provinces (both between 400 and 500 m in disturbed forested habitats). We suspect that this possible undescribed species is much more widespread and will be frequently encountered if surveys can be conducted in intervening localities. This terrestrial species is slightly smaller than the morphologically similar *Platymantis cagayanensis* and *Platymantis* sp. 3 “seeyok,” and calls with a long pulse train, sounding to the human ear like “Yok-yok-yok-yok….”

Cagayan Province—Location 1b: KU 330628–35.

Isabela Province—Location 3: KU 307608–09, 327587; Location 30: no specimens (MVW photo voucher).

***Platymantis* sp. 2 “Cheep-cheep”**

**Figure 26.**
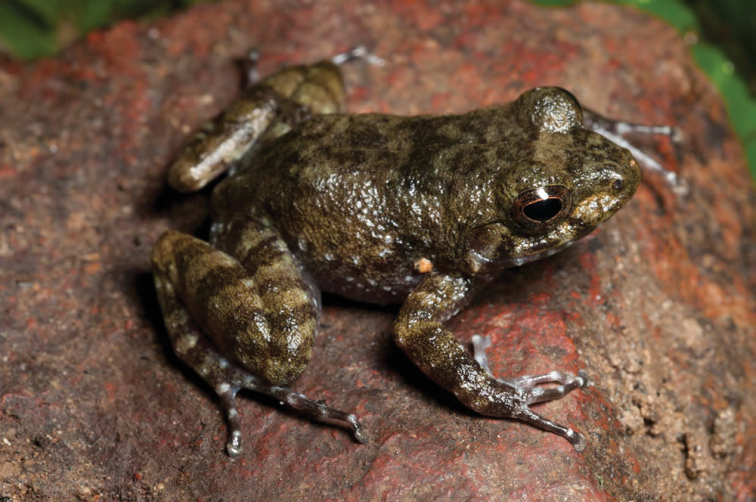
*Platymantis* sp. 2 (“Cheep-cheep;” KU 330606) from the crater of Mt. Cagua, Location 1a. Photo: RMB.

We encountered another potentially distinct species (Fig. 26) of *Platymantis* at both high and low elevation sites on Mt. Cagua, Municipality of Gonzaga. The suspected new species appears phenotypically most similar to *Platymantis lawtoni* from Sibuyan Island (Brown and Alcala 1974; Alcala and Brown 1998, 1999), but is distinguished from other Luzon taxa by its distinct coloration, smooth dorsum, semi-aquatic microhabitat preference, and distinctive “cheep-cheep-cheep…” vocalizations.

Cagayan Province—Location 1a: KU 330588–330600; Location 1b: 330601–615.

***Platymantis* sp. 3 “See-yok”**

**Figure 27.**
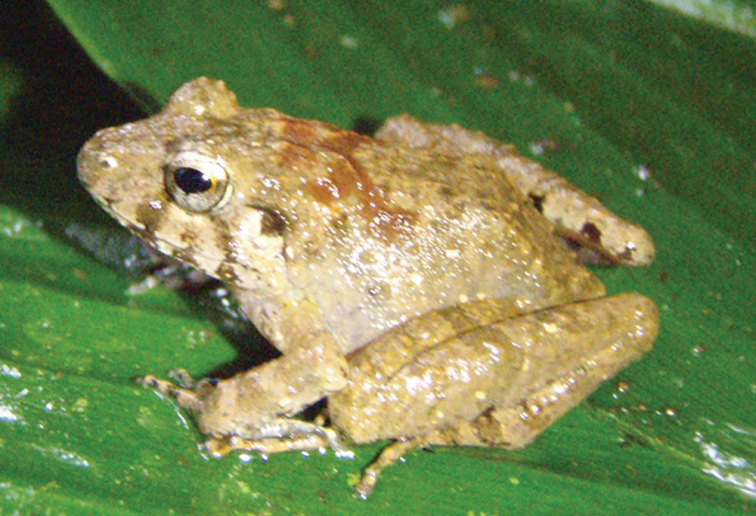
*Platymantis* sp. 3 (“See-yok;” specimen not collected) from Location 36. Photo: MVW.

**Figure 28.**
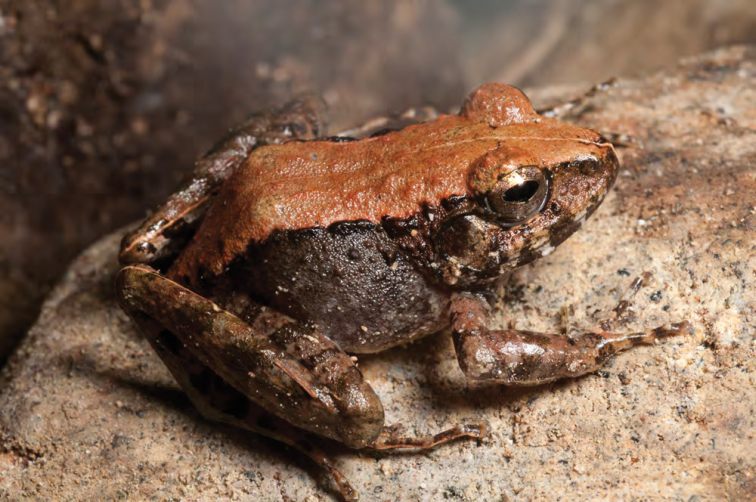
Another color variant of *Platymantis* sp. 3 (“See-yok;” KU 330627) from mid-evelation, Mt. Cagua (Location 1b). Photo: RMB.

This suspected new species (Figs 27, 28) was first observed in Old Balbalan Town (Kalinga Province; RMB and ACD,* personal observations*) and has since been recorded at many sites throughout central and northern Luzon. Morphologically most similar to *Platymantis cagayanensis*, this species can reliably be identified by its silvery iris (versus the bright yellow-orange iris in *Platymantis cagayanensis*) and by distinctive advertisement call, sounding to the human ear like “seee-yok…seee-yok” (Brown et al. *unpublished data*).

Cagayan Province—Location 1b: KU 330618–27, PNM 8678–90.

Isabela Province—Location 36: no specimens (MVW photo voucher).

***Platymantis* sp.**

Without genetic data, information on mating calls, and/or photographs in life, numerous museum specimens of ground-dwelling, medium sized, dorsally tuberculate members of the genus *Platymantis* cannot confidently be identified to species. Many have previously been identified by field collectors as *Platymantis dorsalis* on the basis of generalized morphological similarity to that southern Luzon (type locality: Laguna Bay) species (Brown et al. 1997c; Alcala and Brown 1998, 1999). They are clearly morphologically distinguishable from the terrestrial species *Platymantis corrugatus* (color pattern differences and presence of dorsolateral dermal tubercular ridges in *Platymantis corrugatus*), *Platymantis* sp. 2 “cheep-cheep” (color pattern differences, and absence of any dorsal tubercles in *Platymantis* sp. 2 “cheep-cheep), *Platymantis pygmaeus* (much larger body size), and the arboreal species *Platymantis cornutus*, *Platymantis polillensis*, and *Platymantis sierramadrensis* (all of which have expanded finger and toe pads). With on-going taxonomic work, these specimens may be identifiable to *Platymantis cagayanensis*, *Platymantis* sp. 1 “Yokyok,” *Platymantis* sp. 3 “See-yok” or they may eventually prove to be new, undescribed species.

Cagayan Province—Location 1a: KU 330653–713; Location 1b: KU 330714–16; Location 6: USNM 498524–28; Location 13: USNM 498692–93; Location 15: USNM 498731–34.

Isabela Province—Location 21: KU 307611–17.

### Family Dicroglossidae

***Fejervarya moodiei* (Taylor 1920)**

**Figure 29.**
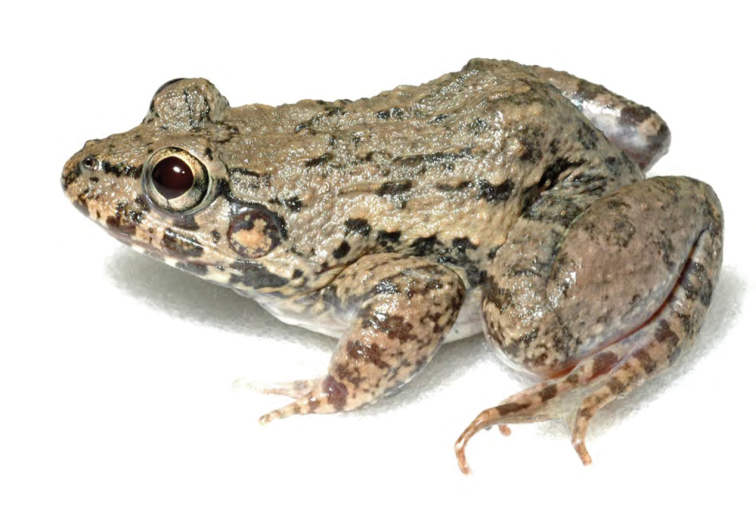
*Fejervarya moodiei* (ACD specimen deposited in PNM) from Ilocos Norte Province. Photo: ACD.

*Fejervarya moodiei* (Fig. 29) is a widespread, endemic estuarine specialist that can be found in a variety of coastal areas including brackish water swamps. Previously considered conspecific with the widespread Southeast Asian species *Fejervarya cancrivora*, recent genetic evidence suggests that the Philippine populations are genetically distinct; the available name for the Philippine population is *Fejervarya moodiei* (Kurniawan et al. 2010, 2011). Widespread and common at most coastal areas throughout the Philippines, this species is clearly most appropriately considered “Least Concern” (LC; IUCN 2011).

Cagayan Province—Location 5: PNM 7424; Location 11: PNM 5654, Location 12: PNM 5654, 5675.

***Fejervarya vittigera* (Wiegmann, 1834)**

*Fejervarya vittigera* is a widespread, low elevation species typically observed in highly disturbed areas with standing water (rice fields, ponds and lakes) or along small, denuded streams near coastal areas or canals bordering agricultural areas. Our specimens were found along muddy stream banks in disturbed forests at the edge of agricultural plantations. Although until now this species has always been considered “Least Concern” (IUCN 2011), and not threatened, recent evidence suggests that populations of this endemic low elevation taxon may be in decline (ACD and M. L. Diesmos, *personal* observation) due to the spread of exceptionally high density populations of the introduced (Diesmos et al. 2006) Asian Bullfrog, *Hoplobatrachus rugulosus* (see below), which appears to displace, out-compete, or otherwise competitively exclude *Fejervarya vittigera* in some areas (Brown et al. 2012).

Cagayan Province—Location 1b: KU 330225; Location 5: USNM 498529–45, 498973–80; PNM 6256–60; Location 6: USNM 498546; Location 11: USNM 498649; Location 14: USNM 498761–62; Location 19: PNM 6191–93.

Isabela Province—Location 21: 307469.

***Hoplobatrachus rugulosus* (Wiegmann, 1834)**

**Figure 30.**
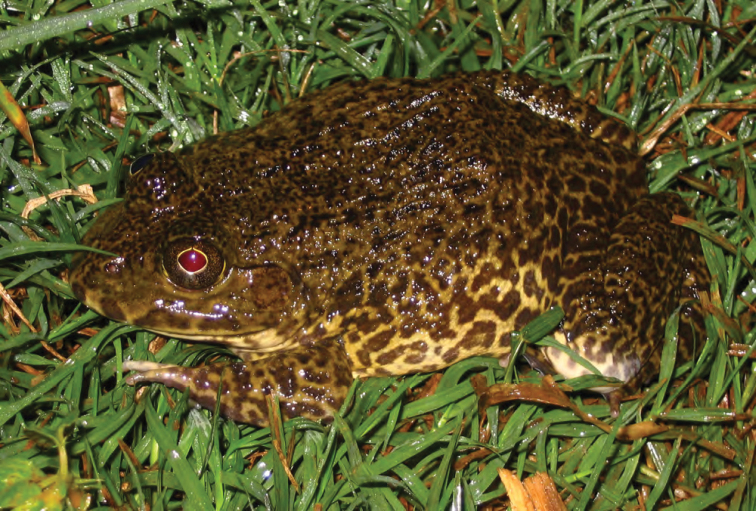
*Hoplobatrachus rugulosus* from Minanga (ACD 3159, deposited in PNM). Photo ACD.

This introduced species (Fig. 30) was first detected in Laguna province in 1996 (Diesmos et al. 2006), but has since been encountered throughout low-lying valley systems bisecting most major islands in the Philippines. *Hoplobatrachus rugulosus* achieves remarkable population densities in large areas of rice cultivation and we have witnessed thousands of individuals in a single day’s hike, actively foraging during day light hours, voraciously hunting any potential prey item (including juveniles of their same species and sympatric congeners; ACD and RMB, *personal observations*). In 2001 RMB and ACD drove the length of the Cagayan Valley, stopping frequently to interview farmers about the densities of frogs in their fields. All reported that these distinctively larger frogs were now the dominant species in the area (and the smaller, previously more common species [presumably *Fejervarya vittigera*] was now far less common). Additionally, in recent trips to Ilocos Norte (Brown et al. 2012), ACD and party found exceptionally high densities of *Hoplobatrachus rugulosus* in agricultural areas and along riverbanks and very few native *Fejervarya vittigera* in the presence of this invasive species.

Cagayan Province—Location 1b: KU 330225; Location 3: PNM 9448.

Isabela Province—Location 21: KU 307488.

***Limnonectes macrocephalus* (Inger 1954)**

**Figure 31.**
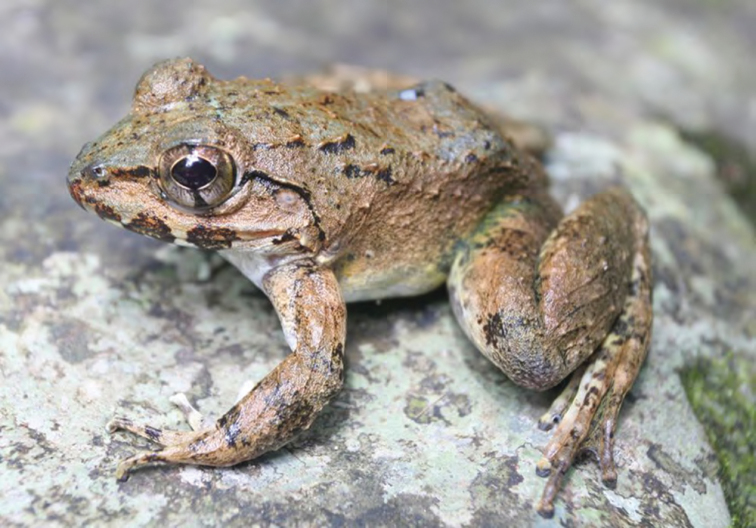
*Limnonectes macrocephalus* (specimens not collected from Dipagsanghan, Location 36. Photo: MVW.

The Luzon fanged frog *Limnonectes macrocephalus* (Fig. 31) inhabits rivers and streams from sea level up to high elevation forests. Although targeted by humans for food and potentially at risk from predation and competition from invasive species (Diesmos et al. 2006), the Luzon fanged frog has always been characterized as common in mid- to high-elevation forests (Brown et al. 1996, 2000a, 2012; Diesmos et al. 2005; Siler et al. 2011a; McLeod et al. 2011). Although this is Luzon’s largest species, low elevation populations, subject to predation by humans and introduced frog species, consistently have a smaller average body size than do high-elevation populations inhabiting inaccessible montane areas (RMB and ACD, *personal observations*). The largest individuals have been documented from small, high-elevation mountain streams that lack above ground connections to large rivers at lower elevations (Brown et al. 2000a; Diesmos et al. 2005). Thus, we assume a lack of connectedness has impeded subsistence harvesting in these areas and *Limnonectes macrocephalus’* indeterminate growth pattern had allowed these populations to achieve high average body sizes (of up to 350–400 g in males) in the absence of human predation.

Cagayan Province—Location 1a: KU 330425–54; Location 1b: KU 330455–69; Location 13: USNM 498704–10, PNM 5888–93; Location 15: USNM 498750–57, 498967–70.

Isabela Province—Location 21: KU 307493–94, 307498–99, 307501–503; Location 22: KU 307506–18; Location 23: KU 327509–17; Location 30: no specimens (MVW photo voucher); Location 36: no specimens (MVW photo voucher).

***Limnonectes woodworthi* (Taylor 1923)**

**Figure 32.**
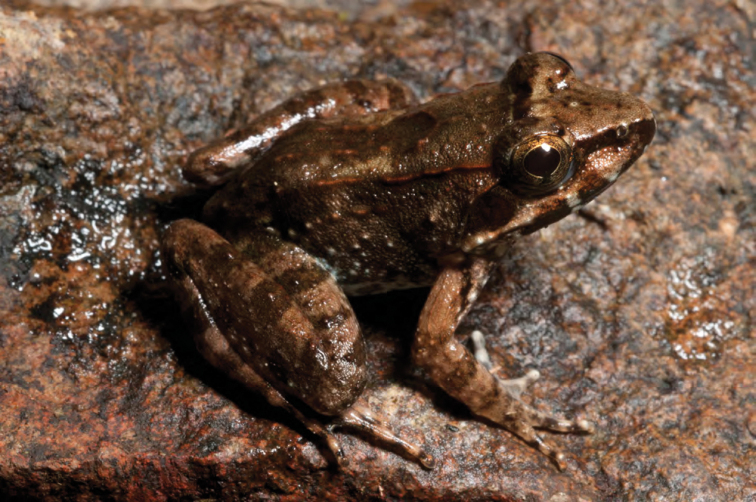
*Limnonectes woodworthi* (KU 330226) from mid-elevation Mt. Cagua (Location 1b). Photo: RMB.

*Limnonectes woodworthi* (Fig. 32) is a commonly encountered stream frog in the mountains of southern Luzon and throughout the Bicol Peninsula (Diesmos 1998; Alcala and Brown 1998; Brown et al. 1996, 2000a); more recent studies have determined that this species may also occur farther north, along the foothills of the Sierra Madre Mountains in Aurora Province (Siler et al. 2011a), Isabela Province (ACD, *unpublished data*), and as far north as Cagayan Province, the Babuyan Islands, and Ilocos Norte Province (Oliveros et al. 2011; Brown et al. 2012). However, the northern populations have a somewhat distinctive color pattern, suggesting they may be taxonomically differentiated. Future studies involving morphometrics, advertisement calls, and genetic data will be necessary to test for the presence of possible species boundaries within *Limnonectes woodworthi*.

Cagayan Province—Location 1a: KU 330227; Location 1b: KU 330226; Location 4: PNM 7523; Location 12: PNM 7522.

Isabela Province—Location 21: KU 307491–92, 307496–97, 307500; Location 23: KU 326471–75.

***Occidozyga laevis* (Günther, 1859)**

**Figure 33.**
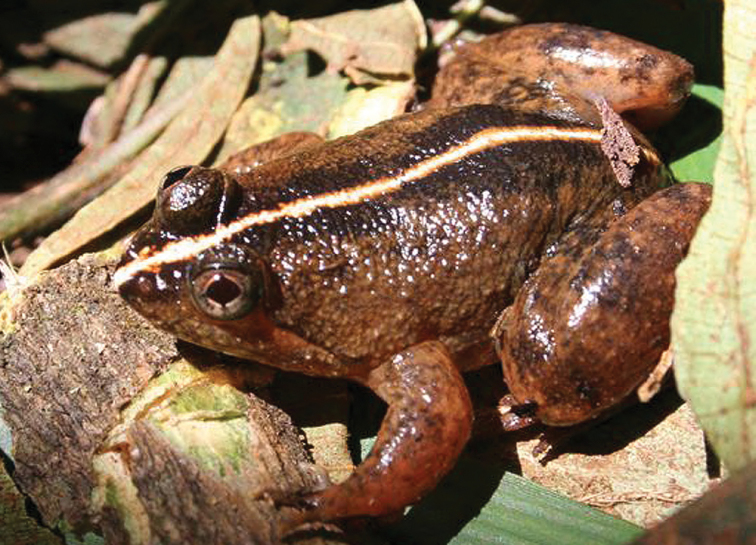
*Occidozyga laevis* (specimens not collected) from Dyadyadin (Location 32). Photo: MVW.

*Occidozyga laevis* (Fig. 33) is a common, widespread species known throughout many of the islands and neighboring continental landmasses of Southeast Asia (Inger 1954, 1999; Inger and Voris 2001). Although we have noted body size and call variation at a few sites in the Philippines (ACD and RMB, *unpublished data*), no taxonomic studies have as of yet targeted this variable taxon. Our specimens were collected along banks of rivers and streams (in quiet side-pools and adjacent puddles), or in puddles on basins on the forest floor, adjacent to flowing water. In the Philippines, individuals aggregate to form breeding groups, with males emitting clicking pulses, sounding to the human ear like the tapping together of small stones.

Cagayan Province—Location 1a: KU 330327–52; Location 1b: KU 330353–61, 330717, PNM 5256–63; Location 2: KU 320164–71, 323421; Location 5: USNM 498519; Location 6: USNM 498520–23; Location 15: USNM 498727–19, 499017.

Isabela Province—Location 21: KU 307540–57; Location 23: KU 326478–79; Location 30: no specimens (MVW photo voucher); Location 36: PNM 5179–86; MVW photo voucher.

### Family Microhylidae

***Kaloula kalingensis* Taylor 1922**

**Figure 34.**
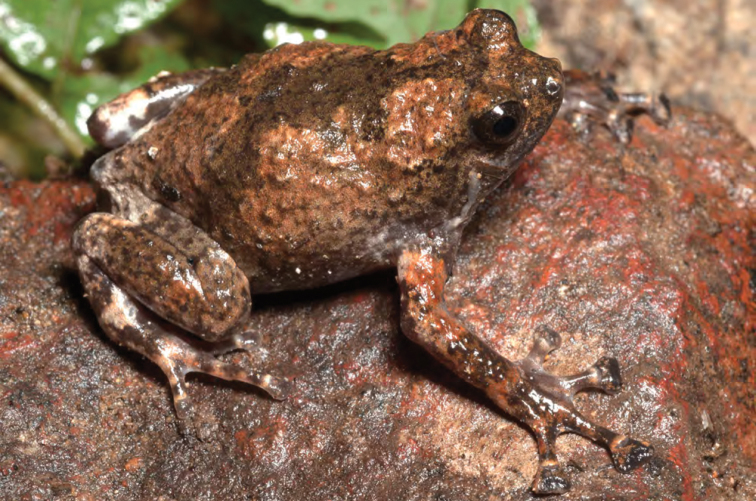
*Kaloula kalingensis* (KU 330273) from forests below the crater of Mt. Cagua (near Location 1a). Photo: RMB.

*Kaloula kalingensis* (Fig. 34) originally was described from Balbalan, Kalinga Province (Taylor 1922a). However, as currently understood, this taxon is now considered common and widespread throughout much of northern Luzon (Brown et al. 1996, 2000a, 2012; Diesmos et al. 2005; Siler et al. 2011a; McLeod et al. 2011; Blackburn et al. in review). Typically encountered in water-filled holes in trees (30–100 cm trunk diameter; holes 1–4 m above the ground) in low to mid-elevation forested areas, this species tolerates high levels of disturbance and is often even found in thick invasive stands of introduced species of bamboo, provided that water-filled cavities provide its favored calling, courtship, breeding, and egg deposition microhabitat (Brown and Alcala 1982; *personal observation*). These observations recently prompted Brown et al. (2012) to downgrade this species IUCN conservation status from “Vulnerable” (IUCN 2011) to “Near Threatened” (IUCN 2010) and our data support this action. However, recent molecular studies by Blackburn et al. (in review) suggest that *Kaloula kalingensis* may be a complex of three or four taxonomically distinct entities, which may result in one or more of these putative species (or, at least significant evolutionary units [ESUs] for conservation) to exhibit a more restricted geographical range. If so, the conservation status of these individual putative species (or ESUs) will need to be individually assessed for conservation threats using field-based data of the actual population abundances and distribution (i.e., not inferences from forest cover).

Cagayan Province—Location 1a: 330264–72; Location 1b: KU 330273–78, PNM 7485–89; Location 11: PNM 7461–63.

Isabela Province—Location 34: no specimens (MVW photo voucher).

***Kaloula picta* (Duméril and Bibron, 1841)**

**Figure 35.**
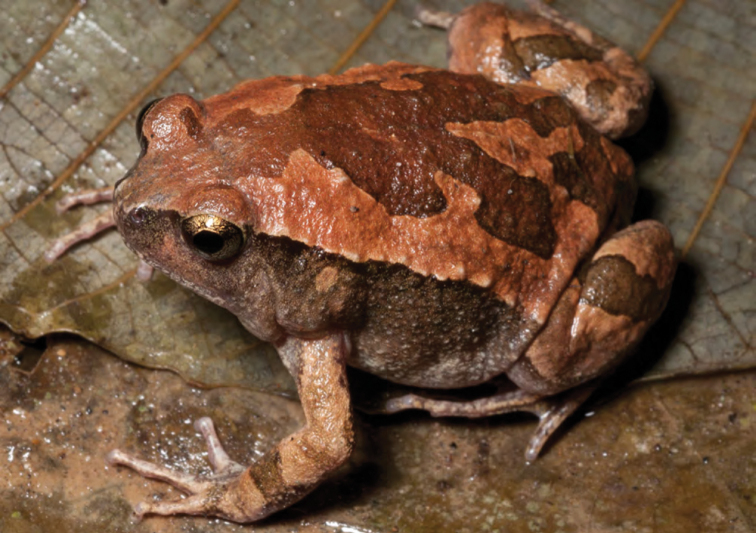
*Kaloula picta* (KU 330616) from the forest edge just above Barangay Magrafil (near Location 1b). Photo: RMB.

*Kaloula picta* (Fig. 35) is a widespread Philippine endemic, distributed widely in low elevation agricultural areas, along riparian habitats in the foothills of mountain systems, and along low-elevation river valleys and coastal areas (Inger 1954; Brown and Alcala 1970a; Alcala and Brown 1998). Nearly genetically identical throughout the archipelago (Blackburn et al. in review), *Kaloula picta* may be another species that has recently undergone rapid range expansion as a result of population transplantation in agricultural shipments, coupled with the conversion of most low-elevation coastal floodplains into its preferred habitat (i.e., flooded rice fields; Brown et al. 2010a).

Cagayan Province—Location 1b: KU 17; Location 5: USNM 498516–17; Location 6: 498518; Location 11: USNM 498638–48, PNM 6701–07; Location 12: PNM 6708–12; Location 14: USNM 498719; Location 15: USNM 498720.

***Kaloula pulchra* Gray, 1825**

*Kaloula pulchra* is an invasive species (Diesmos et al. 2006) only detected in the country in the last decade and suspected of being introduced through the pet trade. This species has become widely distributed on Luzon and several other islands. Recent observations suggest that, in disturbed habitats, *Kaloula pulchra*’s impact on native species may be increasing (Brown et al. 2012). We encountered this species in agricultural areas and heavily disturbed riparian habitats (polluted streams near residential areas) along the Cagayan Valley and wide floodplains surrounding the Cagayan River. It is considered “Least Concern” (LC; IUCN 2011).

Cagayan Province—Location 31: no specimens (ACD field observation).

Isabela Province—Cagayan River banks: no specimens (ACD and RMB field observations).

***Kaloula rigida* Taylor 1922**

**Figure 36.**
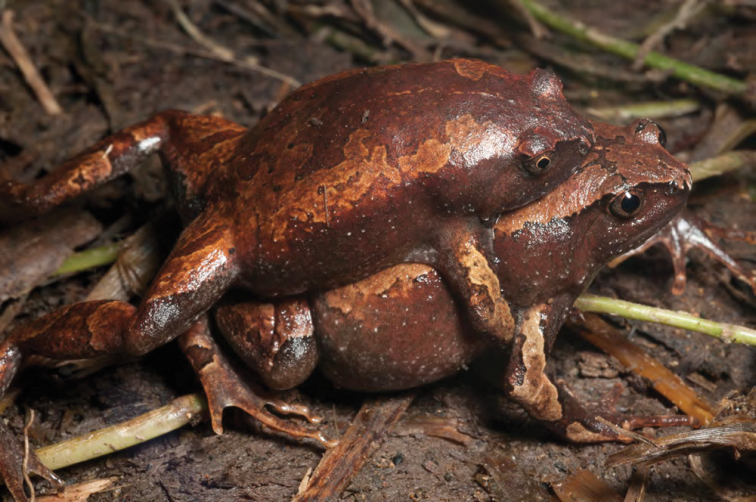
*Kaloula rigida* male (KU 330507) and female (KU 330508) in amplexus, following heavy rains near Location 1b. Photo: RMB.

*Kaloula rigida* (Fig. 36) was described from Baguio City, Benguet Province (Taylor 1922a) and is now known to be widespread in Kalinga, Apayao, Ifugao, and Benguet Provinces of the Cordillera Mountain Range and Isabela and Cagayan Provinces of the northern Sierra Madre (Taylor 1922a; Inger 1954; Alcala and Brown 1998; Diesmos et al. 2005; Brown et al. 2012). This species is a fossorial, ephemeral, pool-breeding specialist that emerges immediately following heavy rains at the onset of the rainy season (June–August) but may be otherwise undetectable if field surveys are conducted in dry months (Brown et al. 2012). Our new records, constituting a major range extension and confirmation of this species continued existence in heavily disturbed forest, further support Brown et al.’s (2012) downgrading of the IUCN (2011) conservation status for this species from “Vulnerable” to “Near Threatened” (IUCN 2010). This species calls in large choruses in temporary pools following heavy rains. Individuals call with repeated pulses, sounding to the human ear like the striking together of two pieces of wood; in large choruses, the collective sound of many individuals calling sounds like a single-stroke engine or small generator.

Cagayan Province—Location 1a: KU 330470–74; Location 1b: KU 330475–515; Location 3: PNM 7492; Location 13: PNM 9666–67; Location 15: USNM 498721–16, 498950, 498963–64, 499016.

Isabela Province—Location 23: KU 326467–70.

### Family Ranidae

***Hylarana similis* (Günther, 1873)**

**Figure 37.**
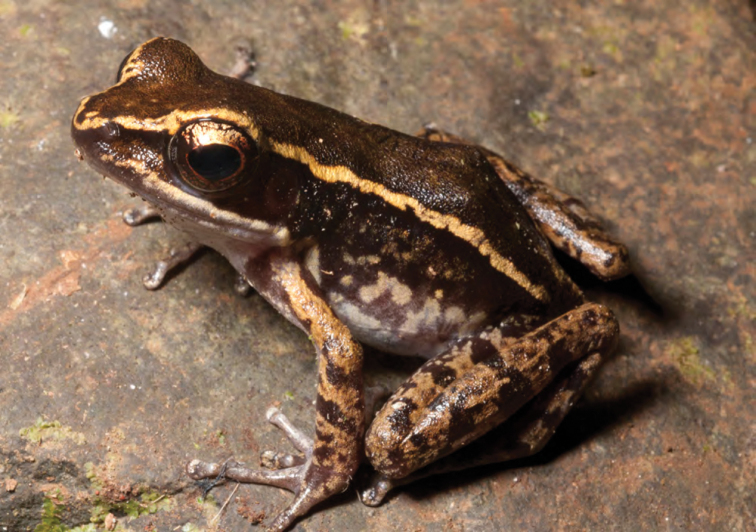
*Hylarana similis* (KU 329815) from mid-elevation, Mt. Cagua, Location 1b. Photo: RMB.

*Hylarana similis* (Fig. 37) is ubiquitous throughout Luzon and associated land-bridge islands (Brown and Diesmos 2002, 2009) where it is locally abundant in all riparian habitats sampled (Brown and Guttman 2002). This species ranges from coastal plains near sea level, to the foothills of all of Luzon’s major mountain ranges, where it is particularly abundant, to mid- and high-elevation forested regions. Without any evidence of population declines and considering its wide distribution, Brown et al. (2012) argued for the downgrading of this species from “Vulnerable (IUCN 2010) to “Near Threatened” (IUCN 2010). This latter designation was considered a compromise because although no declines have been noted (and given current IUCN criteria for assessing conservation threat, this species is most appropriately classified as “Least Concern”), recent studies have determined that this species exhibits high levels of chytrid fungus infection at one low elevation site in southwest Luzon (Swei et al. 2011).

Cagayan Province—Location 1a: KU 330516–63; Location 1b: KU 330564–84; Location 2: KU 320252–65; Location 12: PNM 8378; Location 13: USNM 498711–15, PNM 8301–05; Location 15: USNM 498758–60, 498972.

Isabela Province—Location 23: 326367–69; Location 30: PNM8371; MVW photo voucher; Location 34: no specimens (MVW photo voucher); Location 36: no specimens (MVW photo voucher).

***Sanguirana luzonensis* (Boulenger, 1896)**

**Figure 38.**
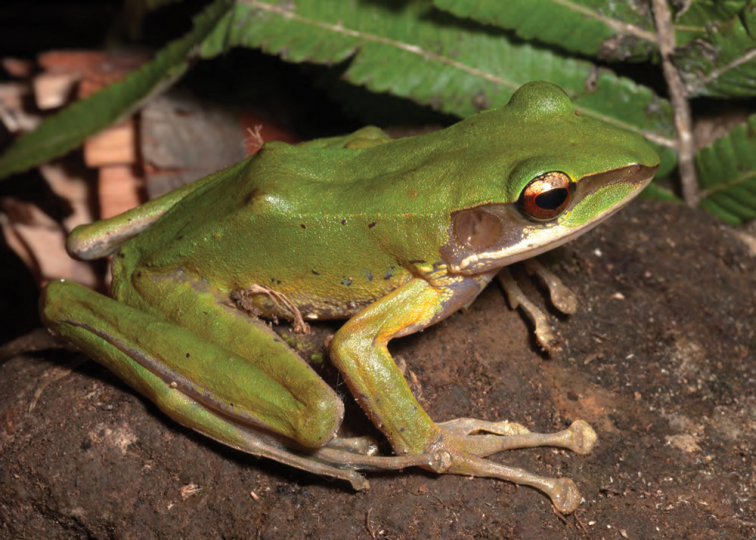
*Sanguirana luzonensis* female (KU 330408) from mid-elevation, Mt. Cagua, Location 1b. Photo: RMB.

This widespread, Luzon faunal-region (Brown and Diesmos 2002, 2009) endemic (Fig. 38) is morphologically variable and exhibits a particularly broad set of habitat tolerances, from coastal waterways, to disturbed lowland riparian habitats, and rivers and streams at the foothills of all Luzon mountain ranges. This species is particularly abundant from low- (200–300 m) to high (up to 1700–1800 m) elevation forested areas and appears quite tolerant of anthropogenic disturbances. It is found on rocks, exposed gravel beds, muddy banks, and low, shrub layer vegetation along nearly all of Luzon’s waterways. The major range extensions and wide variety of habitat types reported here support Brown et al.’s (2012) downgrading of the conservation status (IUCN 2011) of this ubiquitous, disturbance-tolerant species, from “Near Threatened” to “Least Concern” (IUCN 2010). *Sanguirana luzonensis* calls in quiet side pools or when water levels are low and ambient noise is reduced; thus it appears to breed in the late dry season (March–May) and calls with a soft series of descending-frequency “peeps” and “whistles” (Brown et al. 2000a, 2000b; Fuiten et al. 2011).

Cagayan Province—Location 1a: KU 330393-95; Location 1b: KU 330396–424; Location 13: USNM 498694–703, PNM 8128–37; Location 15: USNM 498737–49, 498951–52, 498965–66, 499020–21; Location 16: USNM 498735.

Isabela Province—Location 21: KU 307636; Location 23: KU 326491; Location 30: PNM 8162–66, MVW photo voucher; Location 36: no specimens (MVW photo voucher).

***Sanguirana tipanan* (Brown, McGuire, and Diesmos, 2000)**

**Figure 39.**
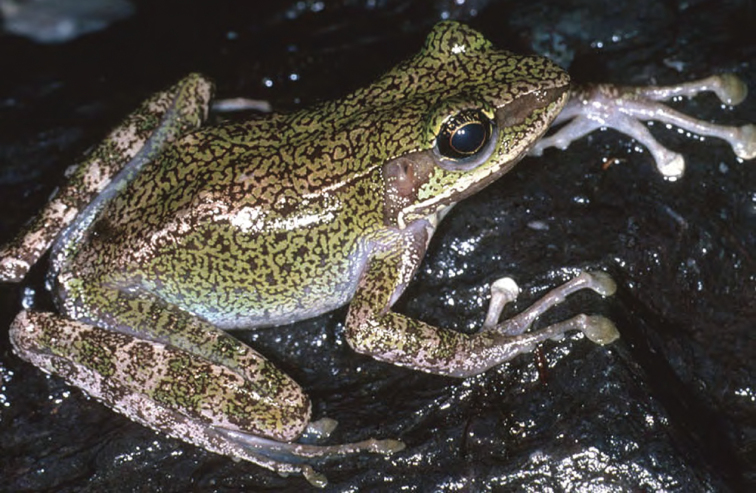
*Sanguirana tipanan* (CMNH 5582) photographed in Aurora Province (Brown et al. 2000a, b). This species has been ovserved at Location 30 by ACD. Photo: J. McGuire.

The presence of this species (Fig. 39), originally described from Aurora Province (Brown et al. 2000a, b), in Palanan has been confirmed by ACD; to date no specimens are available in museum collections from Cagayan or Isabela Provinces. Although no follow-up surveys have been performed in Palanan, additional surveys in Aurora Province (Siler et al. 2011) have documented this taxon at four new sites, suggesting that it probably no longer qualifies for “Vulnerable” (VU; IUCN 2010, 2011) status. This species was not documented in our extensive montane surveys at the northern tip of Luzon (Mt. Cagua, Municipality of Gonzaga). However, until more fieldwork is conducted in the intervening forested mountains of Isabela and Cagayan Provinces to determine the extent of this species range, little can be interpreted from the apparent northern extent of *Sanguirana tipanan*’s occurrence at Palanan.

Isabela Province—Location 30: no specimens (ACD photo voucher).

### Family Rhacophoridae

***Philautus surdus* (Peters, 1863)**

A single specimen of this widespread Luzon-region rhacophorid frog has been collected in Palanan. The loud “crunch…crunch” vocalizations of this species have been heard by the authors at Barangay Nassiping, Municipality of Gattaran.

Cagayan Province—Location 3: no specimens (RMB and ACD field observations).

Isabela Province—Location 30: PNM 5378.

***Polypedates leucomystax* (Gravenhorst, 1829)**

**Figure 40.**
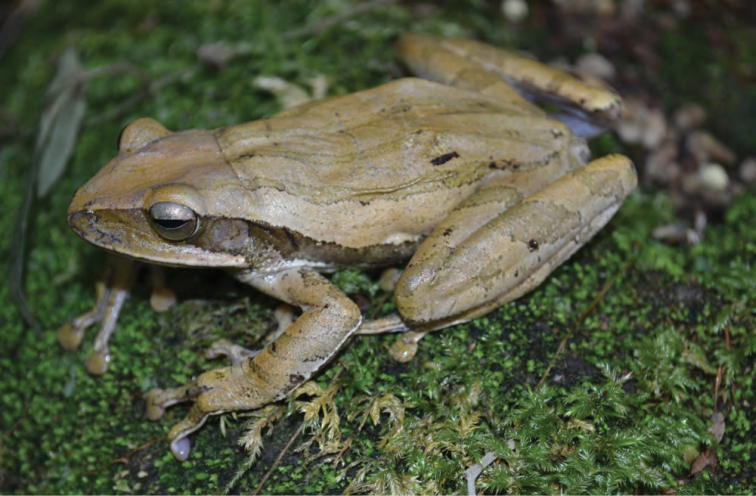
Female *Polypedates leucomystax* (KU 330233) from the crater of Mt. Cagua, Location 1a. Photo: JS.

Philippine *Polypedates leucomystax* (Fig. 40) is a genetically distinct variant of a widespread species complex ranging throughout much of Southeast Asia (Inger 1954, 1999; Brown et al. 2010a; Kuriashi et al. 2012). Within the archipelago, this species is genetically identical throughout most of its range, but with two genetic types occurring in the Mindanao faunal region (Brown and Diesmos 2002, 2009), one of which is shared with northern Borneo and southern Peninsular Malaysia, suggesting two invasions of the Philippines (Brown et al. 2010a). The existence of a widespread single haplotype throughout the Philippines suggested to Brown et al. (2010a) that this distribution may have arisen from demographic range expansion following the last several centuries of habitat conversion and human mediated dispersal throughout the country. This species is known from dry, coastal areas near agriculture, to 1000+ m high in the Northern Cordillera where it has been found in pristine forests at high elevation (Diesmos et al. 2005, 2006). *Polypedates leucomystax* constructs foam nests above water (Brown and Alcala 1982) and calls with loud, single “Craaaak!” or “Plehht!” vocalizations.

Cagayan Province—Location 1a: KU 330233; Location 1b: KU 330230–32; Location 3: KU 307624–30; Location 5: USNM 498547–49, 498981–89, PNM 3886–90; Location 6: USNM 498550; Location 7: USNM 498551–53; Location 8: USNM 498554–58; Location 11: USNM 498650–66, PNM 3891–3907; Location 14: USNM 498763; Location 15: USNM 498765–76, 498992–94; Location 17: USNM 498764.

Isabela Province—Location 21: KU 307631–35; Location 22: 307618–23; Location 33: no specimens (MVW photo voucher); Location 36: PNM 3916.

***Rhacophorus pardalis* Günther, 1859**

**Figure 41.**
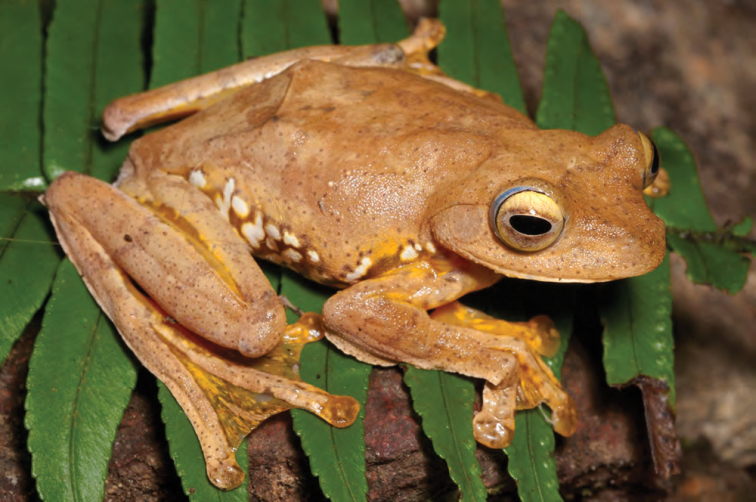
*Rhacophorus pardalis* female (KU 330294) from mid-elevation, Mt. Cagua, Location 1b. Photo: RMB.

This species of Southeast Asian “flying frog” (Fig. 41) is also known from the islands of Indonesia and Malaysia (Brown and Alcala 1994; Alcala and Brown 1998; Inger 1999). In the Philippines it breeds in vegetation above stagnant water in side pools along rivers, water buffalo wallows, or temporary pools in forests. *Rhacophorus pardalis* constructs foam nests above water (Brown and Alcala 1982) and calls with soft “rattle” or “buzz” (*personal observations*).

Cagayan Province—Location 1a: KU 330279–92; Location 1b: KU 330293–99; Location 15: USNM 498777–82, 499023, PNM 5473.

Isabela Province—Location 23: KU 326492–94; Location 32: no specimens (MVW photo voucher); Location 37: PNM 8607.

***Rhacophorusappendiculatus* (Günther, 1858)**

**Figure 42.**
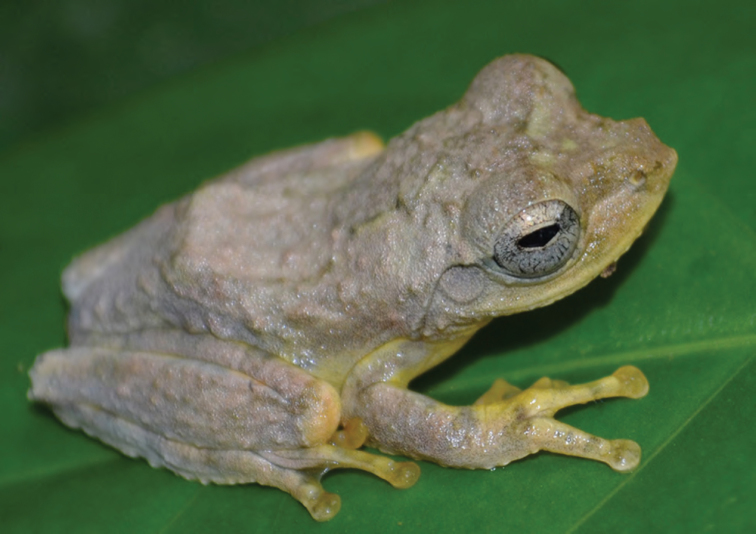
*Rhacophorus appendiculatus* (KU 330228) from the forest in the crater of Mt. Cagua, near Location 1a. Photo: JS.

*Rhacophorus appendiculatus*, althoughconsidered widely distributed (Inger 1954, 1999; Brown and Alcala 1994; Alcal and Brown 1998) on numerous Philippine islands, is patchily distributed on Luzon (Brown and Alcala 1994; Siler et al. 2011; McLeod et al. 2011). We have most often encountered this species following heavy rains, in dense choruses surrounding large temporary swamps or pools in forests of varying degrees of disturbance, and at low- to mid-elevations (300–700 m; see Siler et al. 2011a; McLeod et al. 2011). Two recently collected small specimens (Fig. 42) from high-elevation forests on Mt. Cagua appear to fit this species diagnosis (Brown and Alcala 1994) with the caveat that their small body size and reduced tarsal dermal fringes suggest to us at least the possibility that some morphological variation in this group, and possible taxonomic significance if bolstered by future studies of ecology, morphology, habitat, genetic, and call variation.

Cagayan Province—Location 1a: KU 330228–29.

### Reptilia: Lizards. Family Agamidae

***Bronchocela marmorata* Gray, 1845**

**Figure 43.**
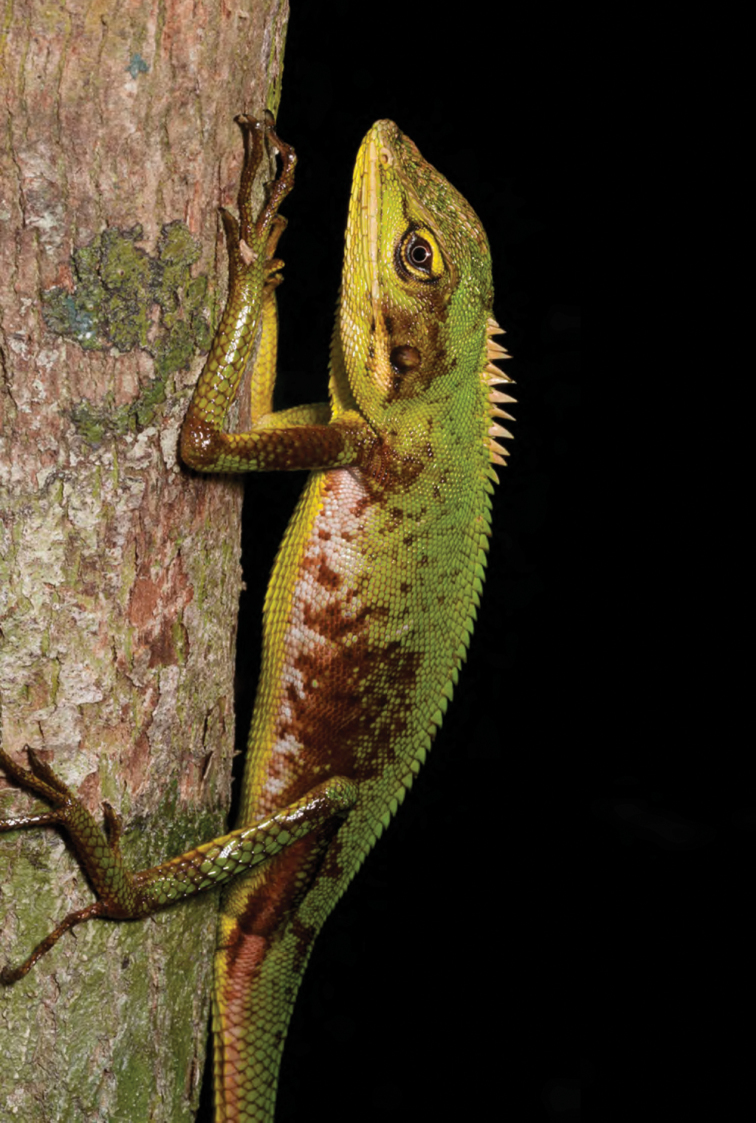
Male *Bronchocela marmorata* (KU 330106) from low-elevation, Mt. Cagua (just above Barangay Magrafil), below Location 1b. Photo: RMB.

We collected individuals of this widespread northern Luzon species (Fig. 43) 4–8 m above the ground in secondary growth trees and agricultural hedgerows. Our specimens are clearly diagnosable, in accordance with Taylor’s (1922b) definition, as *Bronchocela marmorata*. However at numerous sites throughout the southern portions of Luzon (Brown et al. 2000a, 2012; Siler et al. 2011a; McLeod et al. 2011), specimens appear to match the definition of *Bronchocela cristatella* (Kuhl 1820; Hallermann 2005), and yet are genetically identical to specimens that key out to *Bronchocela marmorata* (Hallermann 2005; Brown, Welton, Rock, Siler, and Diesmos, *unpublished data*). These observations suggest the strong possibility that the characters utilized to define these two nominal species’ on Luzon vary clinally and/or ontogenetically. Clearly, further study is warranted; we note that if, as we suspect, only a single species in this group exists on Luzon (as unpublished genetic data would suggest), the correct name for that species would be *Bronchocela marmorata* (Taylor 1922b).

Cagayan Province—Location 1b: KU 330104–06; Location 2: KU 320283–84; Location 3: PMM 7559; Location 13: USNM 498716; Location 15: USNM 498783; Location 20: PNM 7470.

***Draco spilopterus* (Wiegmann, 1834)**

**Figure 44.**
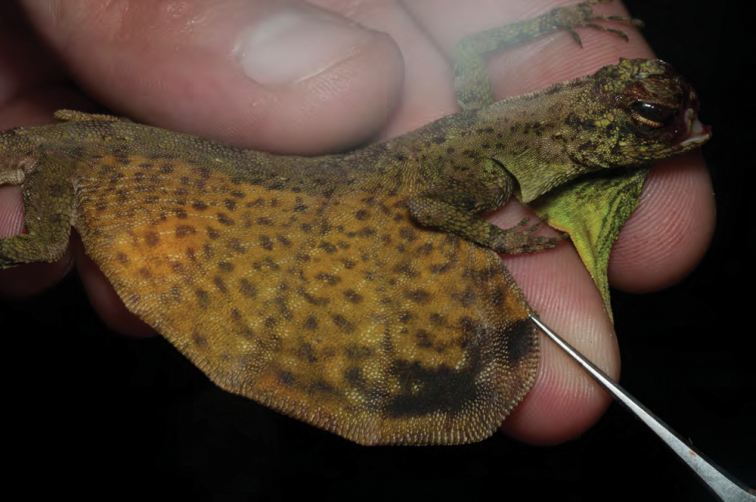
Male *Draco spilopterus* (KU 330062) from low-elevation, Mt. Cagua (just above Barangay Magrafil), below Location 1b. Photo: RMB.

This widely distributed Luzon and Visayan faunal-region (Brown and Diesmos 2002, 2009) endemic achieves particularly high densities in coastal coconut palm plantations, but it is also found at lower densities in disturbed and primary forests throughout the northern Philippines (McGuire and Alcala 2000). Our specimens (Fig. 44) were collected in patchy, disturbed (selectively logged) forests at low elevations, adjacent to clearings caused by shifting, slash-and-burn agriculture.

Cagayan Province—Location 2b: KU 330061–62; Location 3: KU 307457–48, 327734, 327736.

Isabela Province—Location 22: KU 327735; Location 25: KU 327737; Location 26: KU 327739; Location 32: no specimens (MVW photo voucher); Location 36: PNM 1007–08; Location 36: PNM 1011–12.

### Family Gekkonidae

***Cyrtodactylus philippinicus* (Steindacher, 1867)**

**Figure 45.**
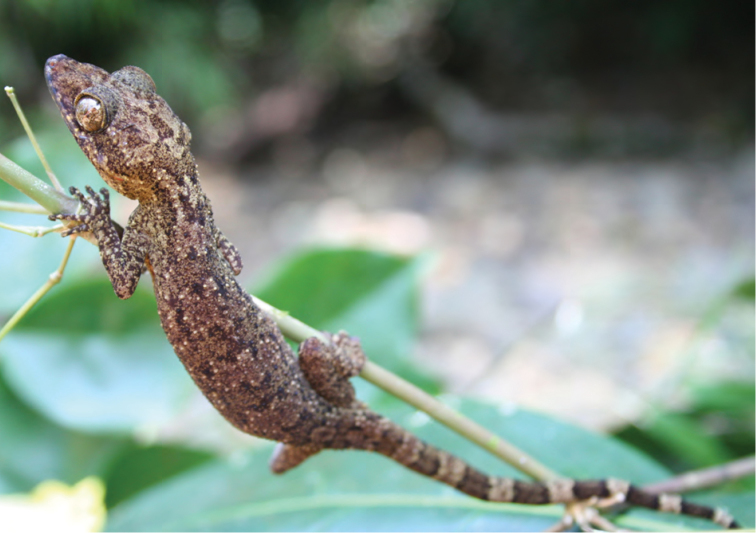
*Cyrtodactylus philippinicus* from Palanan (Location 30). Photo: MVW.

One of the most common squamates in the northern Philippines, *Cyrtodactylus philippinicus* (Fig. 45) is common from low- to mid-elevation forests, at elevations of 800 or 900 m (Brown et al. 1996, 2000a, 2012; Diesmos et al. 2005; Siler et al. 2011a). This species is typically found along riparian habitats, and is active at night on rocks and boulders, over-hanging stumps and logs, or on root balls of large trees, exposed by flowing water.

Cagayan Province—Location 1a: KU 330168–78; Location 1b: KU 330179–97, PNM 1467; Location 20: PNM 1466.

Isabela Province—Location 23: KU 327071–76; Location 26: 327077–78; Location 33: no specimens (MVW photo voucher).

***Gehyra mutilata* (Wiegmann, 1834)**

**Figure 46.**
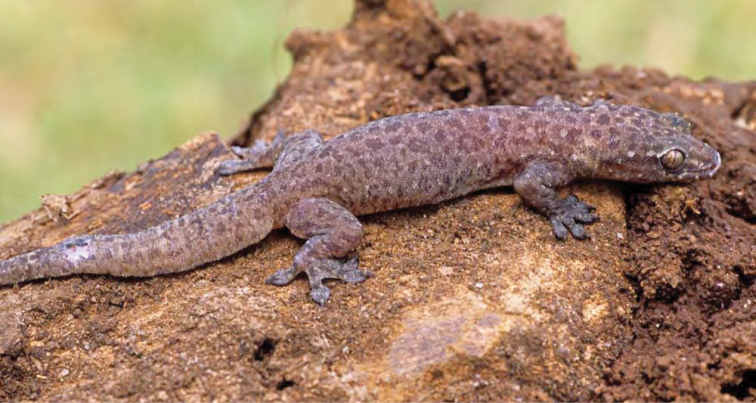
*Gehyra mutilata* (PNM 6501) from Barangay Dibuluan (Location 31). Photo: ACD.

*Gehyra mutilata* (Fig. 46) is a common, widespread “house” gecko that differs from the other most common human commensals (*Hemidactylus platyurus* and *Hemidactylus frenatus*) in that it prefers dark perches, away from overhead lights, probably as a result of competitive interactions with these latter species (Ota 1989). Our specimens were found on darkened walls of houses and on trunks of trees in residential areas.

Cagayan Province—Location 3: KU 307471; Location 5: USNM 498559–61, 499244–46, 499249; Location 7: USNM 498562–63, 499247–48, PNM 5367; Location 9: USNM 498601–05; Location 14: USNM 498791; Location 17: USNM 498786–90, 499250.

Isabela Province—Location 36: PNM 6500–01.

***Gekko gecko* (Linnaeus, 1758)**

**Figure 47.**
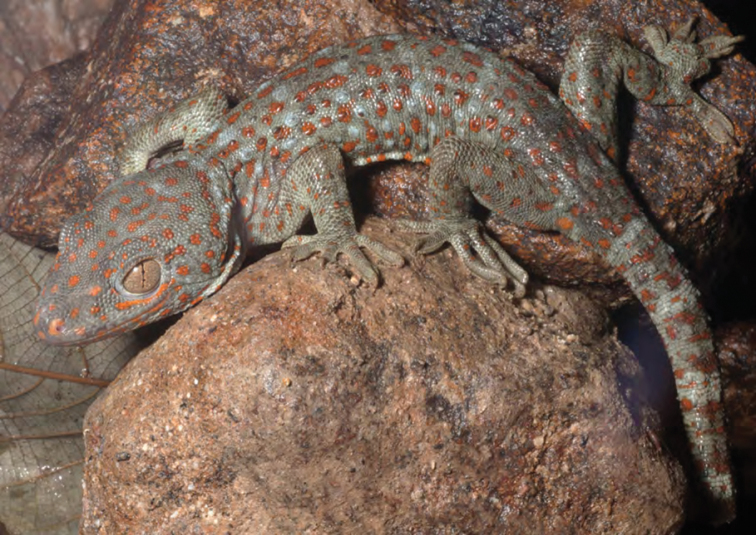
Male *Gekko gecko* (KU 330057) the outskirts of Barangay Magrafil. Photo: RMB.

*Gekko gecko* (Fig. 47) is a widespread Southeast Asian species. In the northwrn Sierra Madre, specimens have been observed on a variety of man-made structures in and around human habitation (but this species is less frequently encountered in forests). Although we heard its distinctive vocalizations at many of the sites we visited in Cagayan and Isabela Provinces (including Locations 2–4, 19, 20–22, 28–30), we seldom endeavored to collect this identifiable and well-known species. One specimen, captured on a house in a small village, was brought to us by a resident of Barangay Magrafil.

Cagayan Province—Location 1b: KU 330057.

***Gekkokikuchii* (Oshima, 1912)**

**Figure 48.**
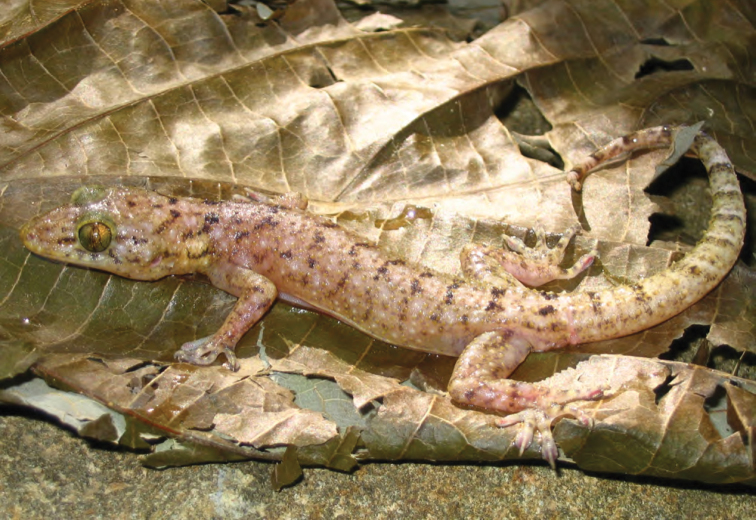
*Gekko kikuchii* (ACD 3077, deposited in PNM) from Barangay Binatug, San Mariano (Location 22). Photo: K. M. Hesed.

*Gekko kikuchii* (Fig. 48) is the name available for the genetically distinct northern Luzon and Lanyu Island (Taiwan) lineage (Siler et al. 2012) of the *Gekko mindorensis* complex (Bauer 1994; Roesler et al. 2011; Siler et al. 2012). This species is found on large boulders in riparian habitats and on concrete structures bordering water (culverts, bridge pylons, walls, and cinderblock buildings). Clearly, the widespread Philippine *Gekko mindorensis* Complex (Taylor 1922; Brown and Alcala 1978; Siler et al. 2012) will require extensive taxonomic revision in the near future (Siler et al. *unpublished data*).

Cagayan Province—Location 11: USNM 340413–21, PNM 4114–22; Location 13: USNM 340376–412; Location 15: USNM 499241–42.

Isabela Province—Location 21: KU 307472; Location 25: KU 327390.

***Hemidactylus frenatus* Duméril & Bibron, 1836**

**Figure 49.**
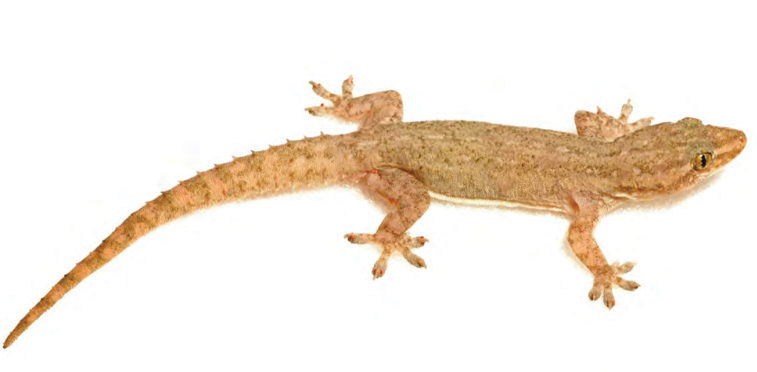
*Hemidactylus frenatus* (specimen in PNM) from Barangay Dibuluan (Location 31). Photo: ACD.

One of the most common “house” geckos (Fig. 49) in the Philippines, this species is frequently encountered under exterior lights of buildings, preying on insects attracted to artificial illumination. It can be easily diagnosed from *Hemidactylus platyurus* by its round tail and smooth, non-frilled flanks.

Cagayan Province—Location 3: KU 307475–76; Location 5: USNM 498564–78, 499251; Location 7: USNM 498579–90, PNM 5368–70, 5490; Location 8: USNM 498591–600; Location 11: PNM 5444–5459; Location 12: USNM 498669; Location 15: USNM 498953–55, 499257; Location 17: USNM 498792–94, 499258; Location 18: USNM 499252; Location 36: PNM 7039.

Isabela Province—Location 21: KU 307477–87.

***Hemidactylus platyurus* (Schneider, 1792)**

Like its congener, *Hemidactylus frenatus*, *Hemidactylus platyurus* is one of the most common “house” geckos in the Philippines and is frequently encountered under exterior lights of buildings, preying on insects attracted to artificial illumination. It is diagnosable from *Hemidactylus frenatus* by the presence of expanded dermal flanges along the sides of the body and by its flattened, transversely expanded tail.

Cagayan Province—Location 3: KU 307442–48; Location 5: USNM 499243, 499253–56; Location 11: USNM 498670–84; Location 12: USNM 498667–68, PNM 6118–19; Location 15: USNM 498795–828; Location 17: USNM 498784–85.

***Hemidactylus stejnegeri* Ota & Hikida, 1989**

Diagnosed as a triploid species (Ota and Hikida 1989a,b), this taxon has only been encountered a few times in recent years (once in downtown Tuguegarao City, ACD *personal observation*). We suspect that it may be widespread and fairly common (and possibly common in existing collections) but that it escapes recognition by herpetologists who may, at a glance, misidentify this species as *Hemidactylus platyurus* or *Hemidactylus frenatus*. Outside of the Philippines, it has been documented in Taiwan and Vietnam (Ota et al. 1993).

Cagayan Province—Location 15: USNM 291834.

***Lepidodactylus* cf. *lugubris* (Duméril & Bibron, 1836)**

**Figure 50.**
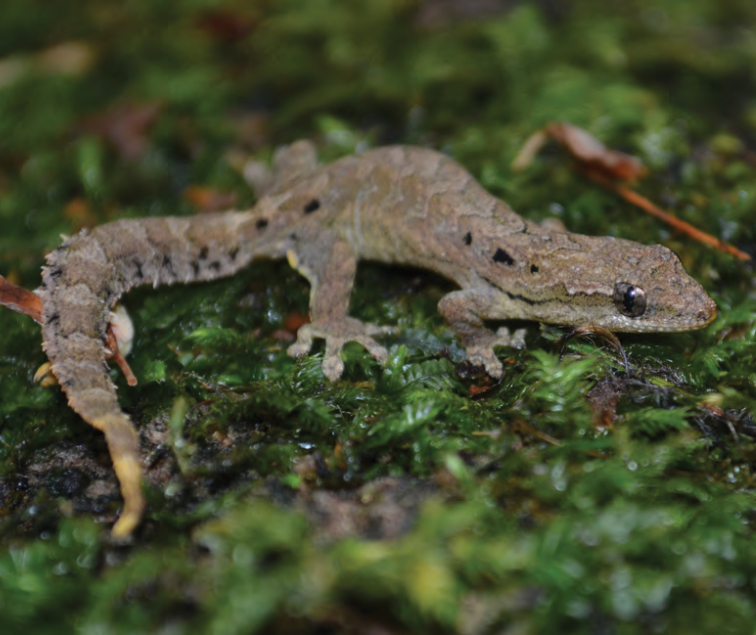
*Lepidodactylus* cf. *lugubris* (KU 330065) from the forested crater of Mt. Cagua, Location 1a. Photo: JS.

Although Brown and Alcala (1978) noted no populations of *Lepidodactylus* on Luzon Island, we have consistently (over the past decade) captured small numbers of specimens (Fig. 50) from a variety of habitats on the Bicol Peninsula, Bulucan, Aurora, Kalinga, Ilocos, and now Cagayan and Isabela provinces (Brown et al. 2000a, 2012; Diesmos et al. 2005; Siler et al. 2011a; McLeod et al. 2011). We continue to identify these as *Lepidodactylus* cf. *lugubris*, albeit with the same reservations as articulated by Brown and Alcala (1978): these populations are quite variable, may represent one or more undescribed species (or triploid clone of *Lepidodactylus lugubris*; R. Fisher, *personal communication*), often resemble *Lepidodactylus planicaudus* (i.e., possessing lateral tail tuberculation; Stejneger 1905 [known from Polillo and Mindoro islands, immediately adjacent to Luzon; Brown and Alcala 1978]), or *Lepidodactylus balioburius* (Ota and Crombie 1989 [known only from Batan Island, north of Luzon; Oliveros et al. 2011]), and/or may be referrable to other possible taxa currently residing in the synonymy of *Lepidodactylus planicaudus* (e.g., *Lepidodactylus naujanensis* Taylor, 1919). Once detailed studies (in particular, with genetic data) become available, the taxonomic status of these Luzon populations will require careful consideration.

Cagayan Province—Location 1b: KU 330065–66.

Isabela Province—Location 22: KU 327729–30.

***Luperosaurus* cf. *kubli* Brown, Diesmos & Duya, 2007**

**Figure 51.**
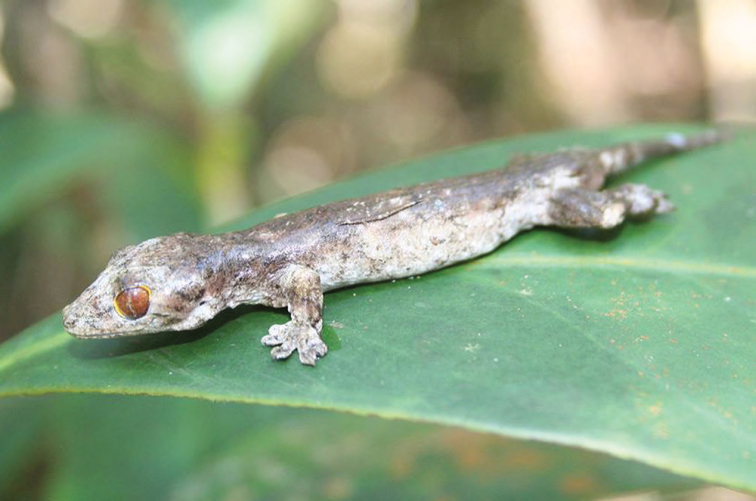
*Luperosaurus* cf. *kubli* (specimen not collected) from Palanan (Location 32). Photo: MVW.

A single specimen provisionally assigned to the rare species *Luperosaurus kubli* (Brown et al. 2007; Fig. 51) has been observed in ultrabasic forests at Dyadyadin but to date, no specimens have been secured. Described from Nagtipunan (Qurino Province, just south of Isabela Province), *Lepidodactylus kubli* has been observed and collected only once (holotype = PNM 9156) and is considered a member of the large, robust-bodied Philippine endemic clade of *Luperosaurus* (Brown et al. 2000c). Like most species of *Luperosaurus*, the microhabitat preference of this species is unknown (Brown and Diesmos 2000).

Isabela Province—Location 32: no specimens (MVW photo voucher).

***Pseudogekko compressicorpus* (Taylor, 1915)**

**Figure 52.**
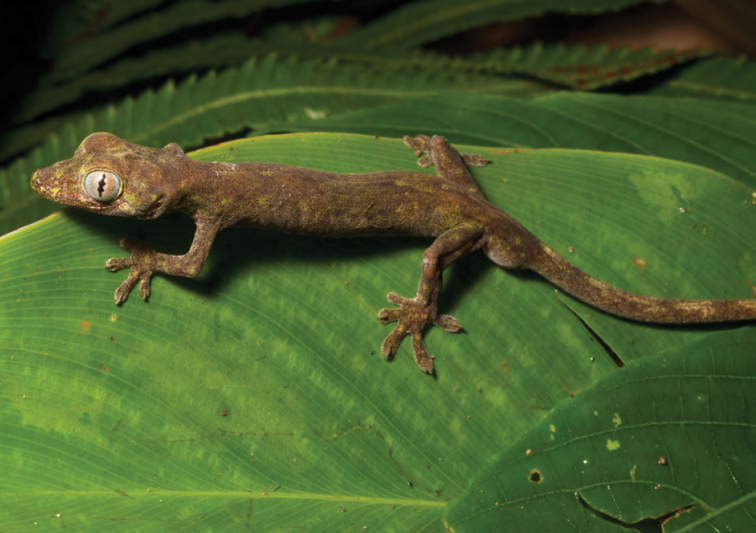
*Pseudogekko compressicorpus* (KU 330058) from 850 m on Mt. Cagua, above Location 1b. Photo: RMB.

As currently defined, this species is widely distributed throughout the Philippines (Fig. 52), from extreme southwestern Mindanao, throughout the eastern island arc (Leyte–Samar) and the Bicol faunal region (including Polillo and Catanduanes islands), and widely throughout the rest of Luzon (Brown and Alcala 1978; Siler et al. 2010a; RMB and CDS, *unpublished data*). This species is typically encountered on large leaves in shrub- and understory layer vegetation, at low- to mid-montane forested sites. Interestingly, it is often encountered on leaves at night following heavy rains (RMB, *personal observation*).

Cagayan Province—Location 2b: KU 330058; Location 3: PNM 8270.

### Family Scincidae

***Brachymeles bicolor* (Gray, 1845)**

**Figure 53.**
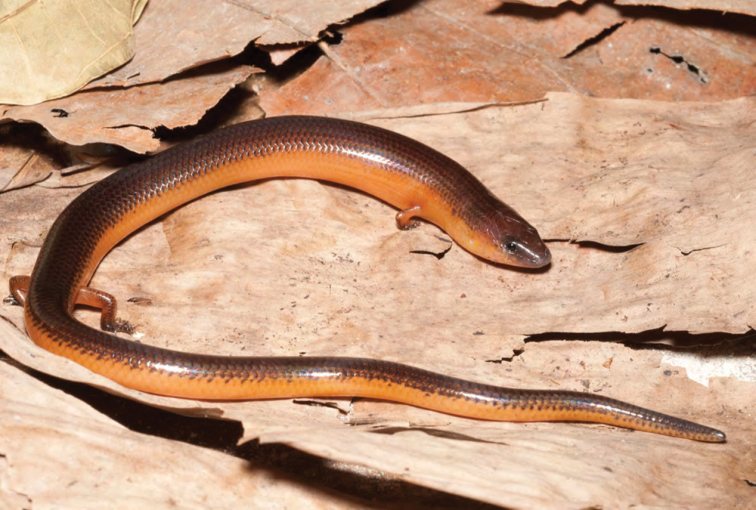
*Brachymeles bicolor* (KU 330074) from mid-elevation, Mt. Cagua, Location 1b. Photo: RMB.

*Brachymeles bicolor* (Fig. 53) has remained one of the Philippines most distinctive and enigmatic skinks since the time of its original discovery (Gray 1845). Detected only a few times since its original description (Brown and Alcala 1980; Brown et al. 2000a; Diesmos et al. 2005), the species was recently redescribed on the basis of material from Aurora, Isabela, and Cagayan Provinces (Siler et al. 2011c). *Brachymeles bicolor* is a forest species that is now predictably found in mid- to high-elevation forests (400–1200 m) and can be located by digging around rotting logs, stumps, and tree buttresses in forests of varying levels of disturbance. Specimens of *Brachymeles bicolor* were caught in both contiguous forests and natural forest remnants at elevations between 150 and 400 m within boundary of San Mariano town, Isabela Prov.

Cagayan Province—Location 1b: KU 330073–78; Location 111 PNM 1341; Location 13: USNM 498717, PNM 1340; Location 15: USNM 498829–33, 498997, CAS 186111.

Isabela Province—Location 23: KU 326112–13; 329452–56; Location 24: KU 329458; Location 26: KU 329459; Location 28: KU 329460; Location 33: no specimens (MVW photo voucher).

***Brachymeles bonitae* Duméril & Bibron, 1839**

**Figure 54.**
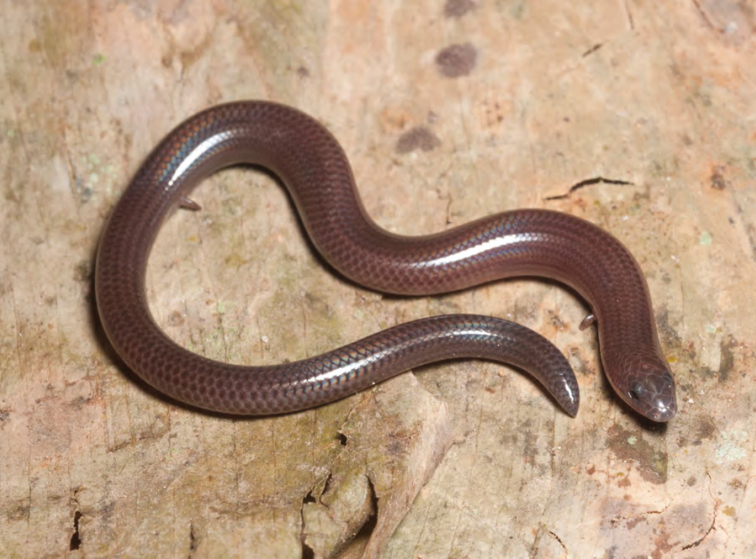
*Brachymeles bonitae* (KU 330100) from mid-elevation, Mt. Cagua, Location 1b. Photo: RMB.

As currently defined, *Brachymeles bonitae* (Fig. 54) is a common, widespread species, endemic to the Luzon faunal region (Brown and Diesmos 2002, 2009), Masbate, Mindoro, Lubang, and Camiguin Norte islands (Brown and Alcala 1980; Oliveros et al. 2011; Siler et al. 2011b). Commonly found within, around, and under rotting logs in loose soil, this slender burrowing skink appears to tolerate varying degrees of forest disturbance, but is usually absent in low-elevation plantations and agricultural areas, where it appears to be replaced by larger, surface dwelling species of *Brachymeles* (CDS, *personal observation*). The low-elevation (150–300 m) forests and coconut palm plantations in the Municipality of Gonzaga (Locations 1b, 2) appear to be an exception in that we found this species commonly in disturbed areas like coconut groves, but in the absence of larger pentadactyl species (e.g., *Brachymeles kadwa*; Siler and Brown 2010). Recent genetic evidence suggests *Brachymeles bonitae* is paraphyletic with respect to *Brachymeles tridactylus* of the Visayan island group (Brown and Alcala 1980; Brown and Diesmos 2002, 2009) suggesting that this may be yet another complex of multiple evolutionary lineages, deserving of taxonomic partitioning (Siler and Brown 2010, 2011; Siler et al. 2011b; 2012; in press).

Cagayan Province—Location 1b: KU 330094–100; Location 2: KU 320468–70, 330101–03; Location 15: USNM 498835–37; Location 16: USNM 498834.

Isabela Province—Location 21: KU 307436; Location 23: KU 326087, 326091–95, 326561.

***Brachymeles kadwa* Siler & Brown 2010**

**Figure 55.**
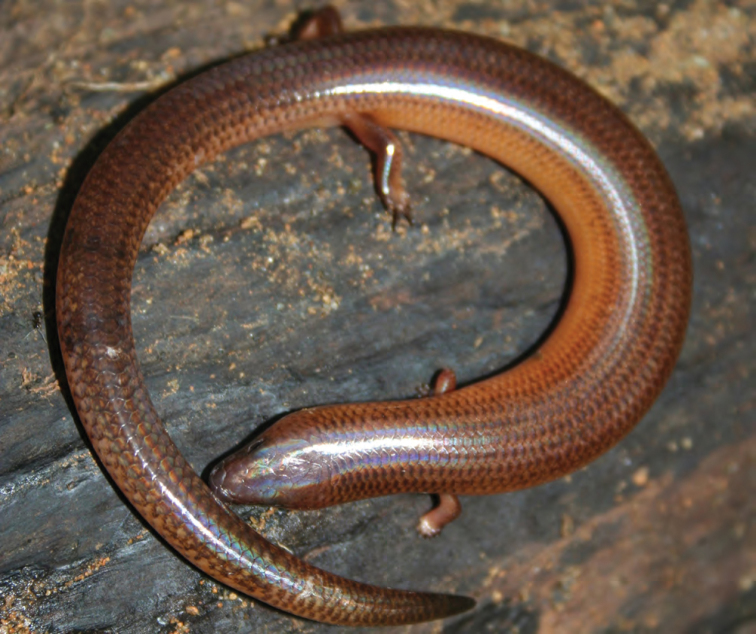
*Brachymeles kadwa* (specimen not collected) from Location 33. Photo: MVW.

This recently described species (Fig. 55) is known from numerous localities across Luzon (Bicol Peninsula, Aurora, Isabela, Laguna, and Cagayan provinces) and also Calayan and Camiguin Norte islands, north of Luzon (Oliveros et al. 2011; Siler and Brown 2010). A large-bodied, pentadactyl species, it is active on the surface in a variety of forest types (with varying levels of disturbance) and, as a result of this activity pattern, it is frequently collected in pitfall traps. Often, it is exceedingly common in low-elevation coconut palm plantations (Siler and Brown 2010; Oliveros et al. 2011).

Cagayan Province—Location 3: KU 307437, 326139–66.

Isabela Province—Location 21: KU 326131–36; Location 24: KU 326124–28; Location 25: KU 326129–30; Location 33: no specimens (MVW photo voucher).

***Brachymeles muntingkamay* Siler, Rico, Duya & Brown 2009**

**Figure 56.**
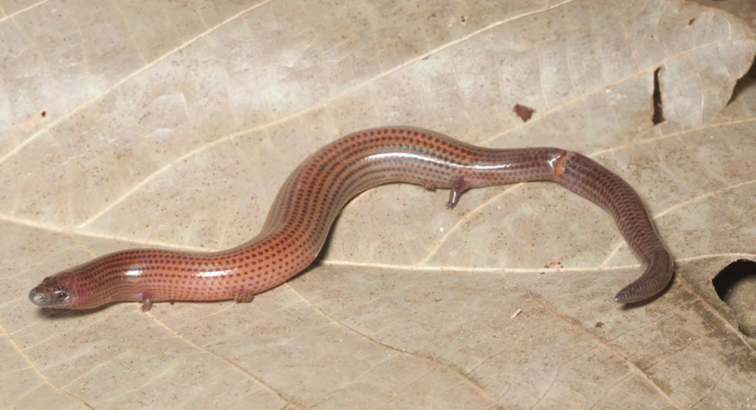
*Brachymeles muntingkamay* (KU 330093) from 900 m on Mt. Cagua, above Location 1b. Photo: RMB.

Recently discovered and described (Siler et al. 2009; Fig. 56), this species previously was known only from Mt. Palali in Nueva Viscaya Province, south-central Luzon (Caraballo Mountain Range). We were quite surprised to find a large population of this distinctive species so far north in the northern Sierra Madre; genetic analysis confirms that the northern population is closely related to the southern, Mt. Palali lineage, suggesting that this species most likely occurs at intervening localities in mid- to high-elevation, reasonably intact forests (700–1000 m) in four or five provinces (Siler et al. in press a). Specimens at these known locations were found inside rotten logs or in soil beneath rotting logs; none were collected in pitfall traps, suggesting that their largely fossorial lifestyle may have prevented discovery until targeted survey efforts associated with CDS’s work (Siler et al. 2009, 2010, Siler and Brown 2011) resulted in their detection. Recent findingssuggest that *Brachymeles muntingkamay* was also observed in a patch of lowland forest in Nassiping, San Mariano (Isabela Prov.), at 60 masl, midway between Mt. Cagua (Cagayan Prov.) and Mt. Palali (Quirino Prov.).

Cagayan Province—Location 1a: KU 330086–89; Location 1b: KU 330090–93; Location 2: KU 327347.

Isabela Province—Nassiping: no specimens (ACD photovoucher)

***Eutropis cumingi* (Brown & Alcala 1980)**

**Figure 57.**
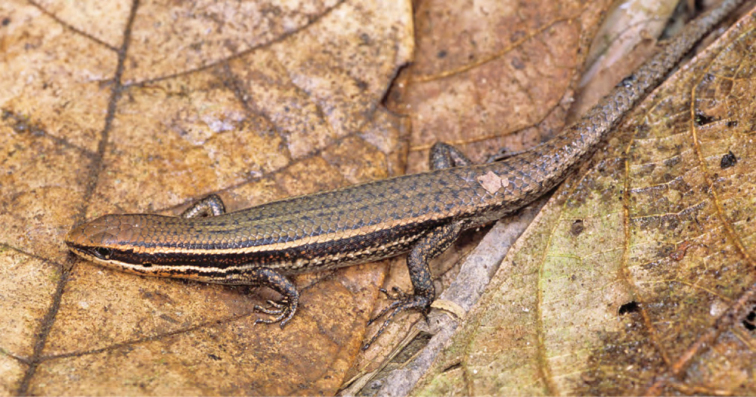
*Eutropis cumingi* (uncataloged specimen in PNM) from mid-elevation, Mt. Cagua, Location 1b. Photo: ACD.

*Eutropis cumingi* (Fig. 57) was described in 1980 from several small series of specimens from Subic Bay, southwest Luzon (CAS 15473; holotype, and CAS 15452, 15454–56, and 15472, 60955–64, paratypes), “northern Luzon” (FMNH 161666–68, paratypes), Ifugao (FMNH 177299–300, paratypes), and generally, “Luzon” (exact locality unknown: FMNH 177304–09, 177311, paratypes). Given its wide distribution, we are not surprised to find specimens diagnosable as this species in the northern Sierra Madre. Past studies have also found it present in the Babuyan and Batanes islands to the north, on Lanyu Island near Tawian (Oliveros et al. 2011), and in the northern Cordillera of Luzon (Diesmos 2008; Diesmos et al. 2005). This species is identified on the basis of its small body size, distinctive scalation, and bright red-orange coloration on the throats of males.

Cagayan Province—Location 15: USNM 498999–9011.

Isabela Province—Location 23: KU 327365, 327376–82; Location 24: KU 327383, 327386; Location 26: KU 327384–85, 327387–88.

***Eutropis multicarinata borealis* (Brown & Alcala 1980)**

**Figure 58.**
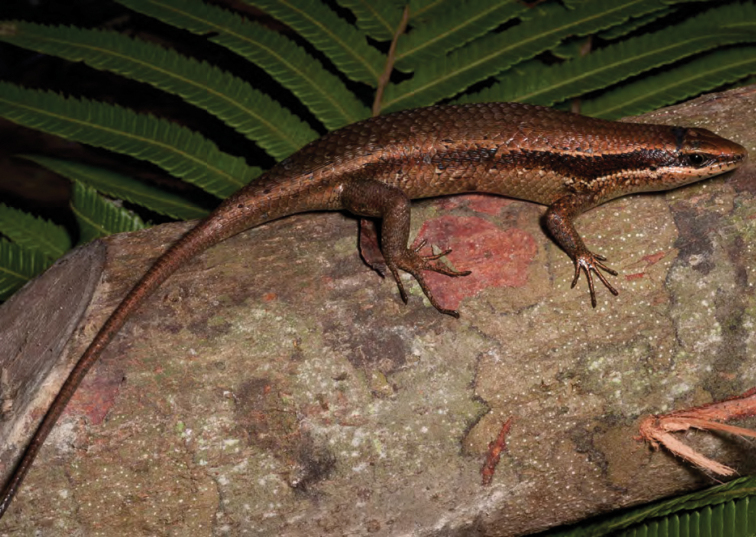
*Eutropis multicarinata borealis* (KU 330072) from mid-elevation, Mt. Cagua, Location 1b. Photo: RMB.

Known from the northern Philippines and Lanyu Island near Taiwan, this species is considered widespread throughout Luzon and associated smaller island groups (Brown and Alcala 1980; Brown et al. 1996, 2000a, 2012; Oliveros et al. 2011; Diesmos et al. 2005; Siler et al. 2011). *Eutropis multicarinata borealis* (Fig. 58) exhibits inordinate amounts of geographically-based body size, scalation, and color pattern variation (Brown and Alcala 1980), suggesting to us that it may be composed of multiple independent evolutionary lineages worthy of taxonomic recognition. Future genetic studies with dense geographical sampling will be necessary to test the hypothesis of a single species, composed of only two subspecies (Brown and Alcala 1980).

Cagayan Province—Location 1a: KU 330070; Location 1b: KU 330071–72; Location 11: PNM 5462–66.

Isabela Province—Location 21; PNM 9519, 9566; Location 23: KU 327366–67, 327533; Location 24: PNM 674, 1385–86; Location 26: KU 327533; Location 28: KU 327530–32; Location 36: PNM 6507, 6517.

***Eutropis multifasciata* (Kuhl, 1820)**

**Figure 59.**
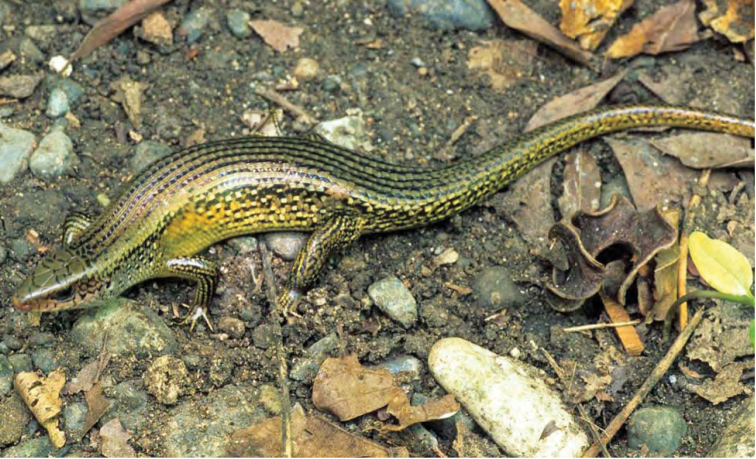
*Eutropis multifasciata* (uncataloged specimen in PNM) from Barangay Dibuluan, San Mariano (Location 31). Photo: ACD.

This widespread Southeast Asian species (Brown and Alcala 1970a; Manthey and Grossman 1997; Fig. 59) is commonly encountered in the Philippines at lower elevations (coastal areas to several hundred meters in elevation) along edges of agricultural land surrounding disturbed forest patches. *Eutropis multifasciata* is active in open sun at midday and can be observed actively forging in the open and retreating into nearby shrubs when disturbed. This species is notable for its striking color polymorphism on lateral surfaces (bright green, orange, or yellow display surfaces), often with multiple color patterns exhibited within the same population.

Cagayan Province—Location 3: KU 307538–39; Location 5: USNM 305883, 498608–09; Location 6: USNM 498606–07; Location 7: USNM 498610–17; Location 11: USNM 498686–91, PNM 669, 1205, 1208, 1267, 1269, 1271, 1291, 1294, 1313; Location 15: USNM 498876–95, 498957; Location 16: USNM 498840–45; Location 17: USNM 498846–75.

Isabela Province—Location 21: KU 327560–61; Location 22: KU 307537; Location 23: KU 327534–48; Location 24: KU 327548–54; Location 25: KU 327555–59; Location 26: KU 327563–66; Location 28: KU 327567–69.

***Lamprolepis smaragdina philippinica* Mertens, 1829**

**Figure 60.**
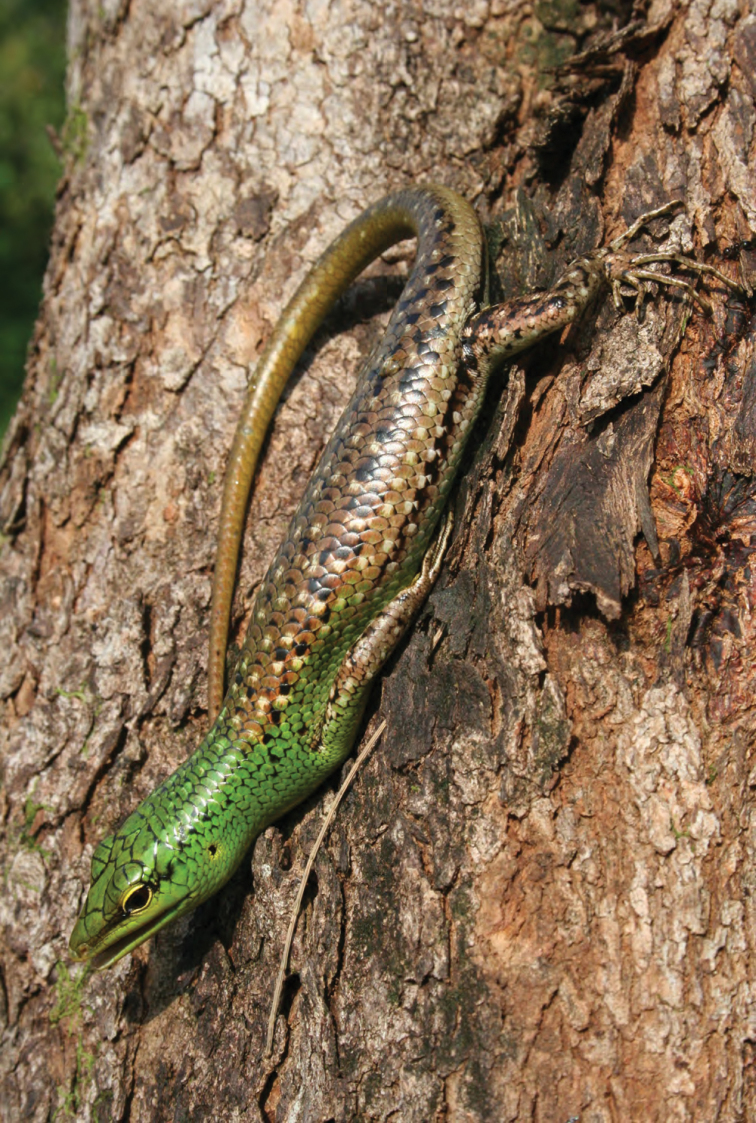
*Lamprolepis smaragdina philippinica* (specimen not collected) from Palanan (Location 33). Photo: MVW.

*Lamprolepis smaragdina philippinica* (Fig. 60) is one of the most locally abundant lizards in coastal areas throughout the archipelago. Also encountered in agricultural plantations (coconut palm plantations, avocado, cacao, and mango plantations) and in regenerating forest nurseries and riparian corridors, *Lamprolepis smaragdina philippinica* exhibits geographically based color variation, with fully green individuals at some localities, brown patches on the head and dorsal surfaces or forelimbs at other sites, and all gray-brown individuals at two known areas (Siler and Linkem 2011). These observations suggest to us that taxonomic partitioning of this species will most likely be necessary with future study (Linkem et al. 2012).

Cagayan Province—Location 1b: KU 330054; Location 3: KU 307489; Location 11: USNM 498685; Location 12: PNM 5461; Location 15: USNM 498838–39, 498998, PNM 5474; Location 20: PNM 7471.

Isabela Province—Location 23: KU 326564; Location 33: no specimens (MVW photo voucher); Location 36: PNM 6728.

***Lipinia* cf. *vulcania* Girard 1857**

**Figure 61.**
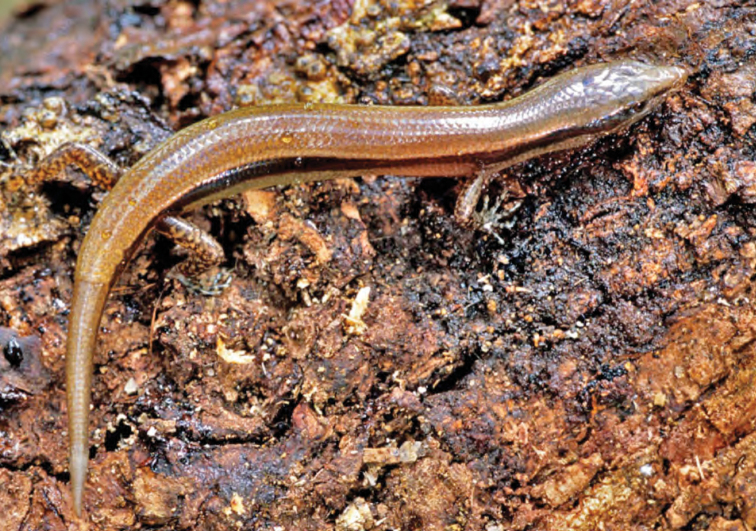
*Lipinia* cf .*vulcania* (specimen in PNM) from Barangay Dibuluan, San Mariano (Location 30). Photo: ACD.

Girard’s (1857) single specimen of this distinctive species reportedly originated at1700 m asl on Dapitan Peak, Zamboanga Peninsula, of Mindanao Island; this unique specimen is now presumed lost (Brown and Alcala 1980). With some hesitation, Brown and Alcala (1980) referred an additional specimen from Luzon (specific locality data unknown) to *Lamprolepis vulcania*. We suspect the two available specimens from Luzon (CAS 16472 and ACD [PNM] 2036) will eventually be recognized as a new species if researchers can visit the type locality on Mindanao and secure additional comparative material that would allow for a thorough taxonomic study. The Luzon population we refer to *Lamprolepis vulcania* cf. *vulcania* (Fig. 61) is most likely an undescribed, but related species.

Isabela Province—Location 23: ACD 2036, deposited in PNM.

***Otosaurus cumingi*Gray 1845**

**Figure 62.**
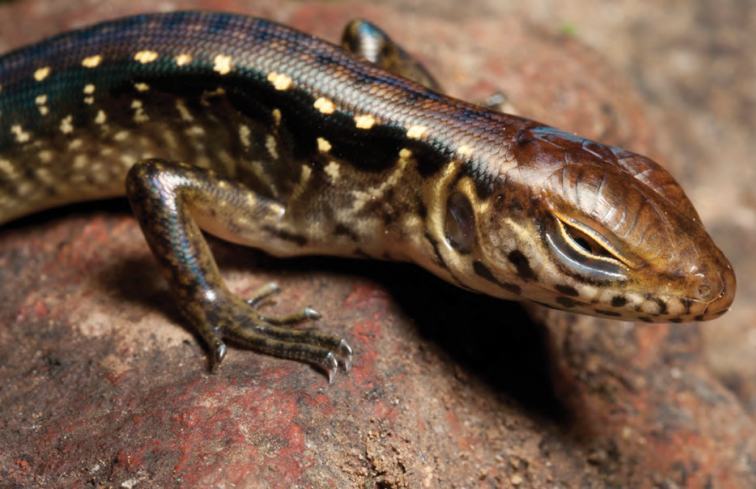
*Otosaurus cumingi* juvenile (KU 330652) from mid-elevation, Mt. Cagua, Location 1b. Photo: RMB.

**Figure 63.**
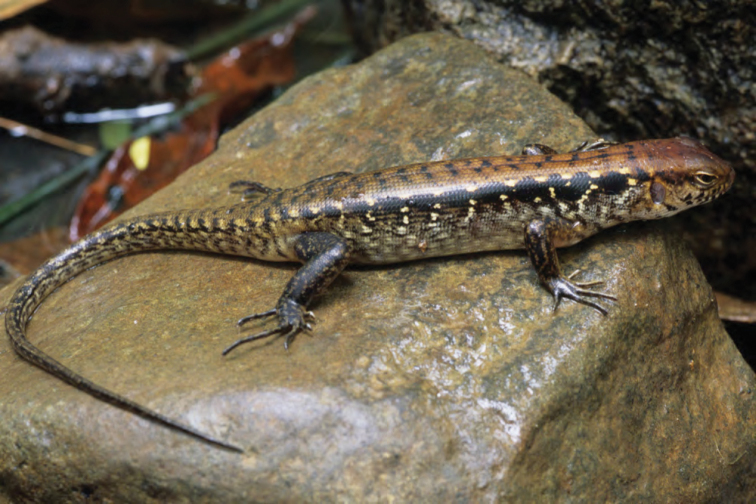
*Otosaurus cumingi* adult male (PNM 8484) mid-elevation, Mt. Cagua, Location 1b. Photo: ACD.

This is the largest Philippine species in the *Sphenomorphus* Group (Greer and Parker 1967) and was formerly referred to the genus *Otosaurus* (Taylor 1922b), but later transferred to *Sphenomorphus* (Brown and Alcala 1980). New phylogenetic studies by Linkem et al. (2011) have resulted in the resurrection of a monotypic *Otosaurus* to accommodate this highly distinctive Philippine “giant” *Sphenomorphus* Group species. *Otosaurus cumingi* (Figs 62, 63) is most frequently encountered active at midday in mid-elevation forests (200–500 m) of varying levels of disturbance. It is a wide-ranging species, and can be encountered on the forest floor, not necessarily confined to riparian corridors or woody debris microhabitats.

Cagayan Province—Location 1b: KU 330063–64, 330652, PNM 8484; Location 15: USNM 498904.

Isabela Province—Location 23: KU 326581–82, PNM 671; Location 31: (specimen in PNM).

***Pinoyscincus abdictus aquilonius* (Brown & Alcala 1980)**

**Figure 64.**
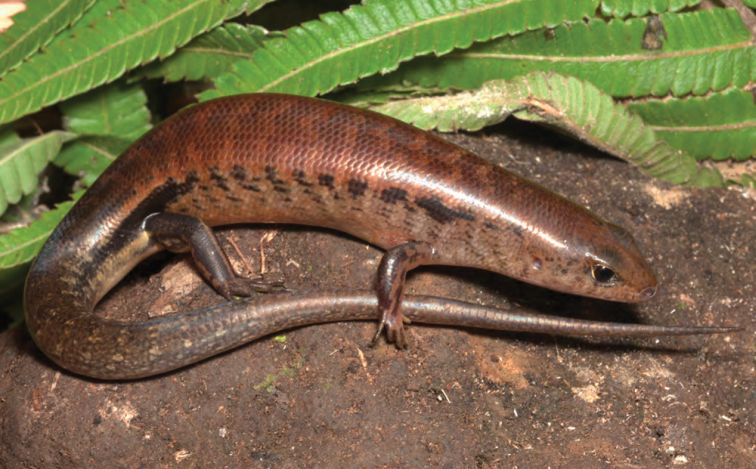
*Pinoyscincus abdictus aquilonius* (KU 330206) low-elevation, Mt. Cagua, below Location 1b. Photo: RMB.

Recently transferred from the paraphyletic genus *Sphenomorphus* to the newly recognized genus *Pinoyscincus* (Linkem et al. 2011) on the basis of a multilocus phylogenetic analysis and a survey of new morphological characters, *Pinoyscincus abdictus aquilonius* (Fig. 64) is a medium-bodied forest species with a preference for intact, low- to mid-elevation habitats (100–600 m) with minimal disturbance (Taylor 1922b; Brown and Alcala 1980; Brown et al. 2000a, 2012; Siler et al. 2011; McLeod et al. 2011).

Cagayan Province—Location 1a: KU 330198–05; Location 1b: KU 330206–21, 330224; Location 2: KU 320497, 330222–23; Location 15: USNM 498897–903; Location 16: USNM 498896.

Isabela Province—Location 21: KU 307677, 327657–58; Location 22: KU 327664; Location 23: KU 326568–69, 327642, 327643–53; Location 24: KU 327659–63; Location 25: KU 327654–56; Location 30: no specimens (MVW photo voucher); Location 36: no specimens (MVW photo voucher).

***Parvoscincus decipiens* (Boulenger, 1895)**

Recently transferred from the paraphyletic genus *Sphenomorphus* to an expanded *Parvoscincus* (Ferner et al. 1997)on the basis of a multilocus phylogenetic analysis and a survey of new morphological characters (Linkem et al. 2011), *Parvoscincus decipiens* (Fig. 61) is a small-bodied forest species with a preference for intact, mid- to high-elevation habitats (400–1200 m) with minimal disturbance (Taylor 1922b; Brown and Alcala 1980; Brown et al. 2000a, 2012; Siler et al. 2011; McLeod et al. 2011).

Cagayan Province—Location 1a: KU 330119–22; Location 1b: KU 330067–69, 330123–30, PNM 8486; Location 3: KU 326718: Location 5; PNM 5371–72; Location 6: USNM 498618–25; Location 13: PNM 8427; Location 15: USNM 498906–12; Location 16: USNM 498905.

Isabela Province—Location 22: KU 326585, 326603, 326715; Location 21: PNM 6544; Location 23: KU 326586, 325592–96, 326706–08; Location 24: KU 326560, 326604–12; Location 25: 326597–602, 326709–12; Location 26: KU 326713–14, 327429; Location 27: 326716–17; Location 30: PNM 6531, 6538, 6541; Location 36: no specimens (MVW photo voucher).

***Parvoscincus* cf. *decipiens***

A second small leaf-litter skink species, related to *Parvoscincus decipiens* (Linkem and Brown in review; Fig. 66) is present at two localities in Cagayan and Isabela Provinces. With near identical ecological habits and microhabitat preference, this undescribed new species is only distinguishable on the basis of coloration, a few differences in scalation, and pronounced genetic variation (Linkem and Brown in review).

Cagayan Province—Location 1b: KU 330120, 330122, 330126, 330128.

Isabela Province—Location 23: KU 329951, 330067–68, 330119.

**Figure 65.**
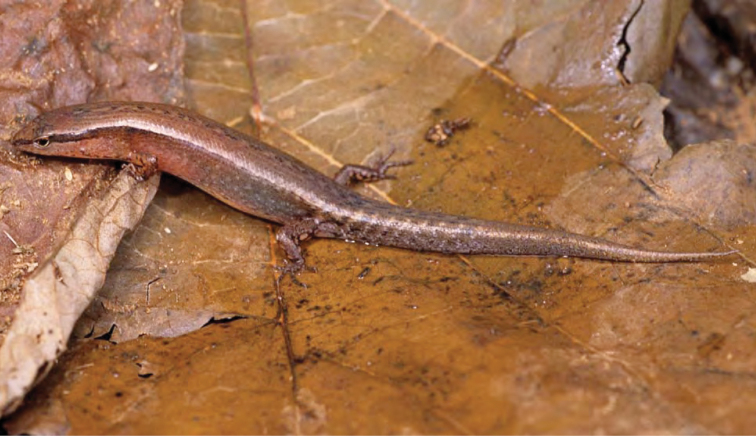
*Parvoscincus decipiens* (KU 326609) from Barangay Dibuluan, San Mariano (Location 31). Photo: ACD.

**Figure 66.**
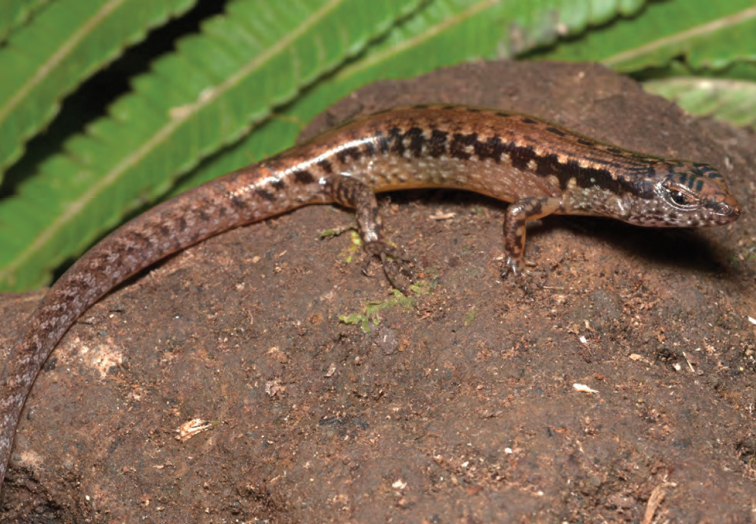
*Parvoscincus* cf. *decipiens* (KU 330124) from 900 m asl, Mt. Cagua, above Location 1b. Photo: RMB.

***Parvoscincus leucospilos* (Peters, 1872)**

**Figure 67.**
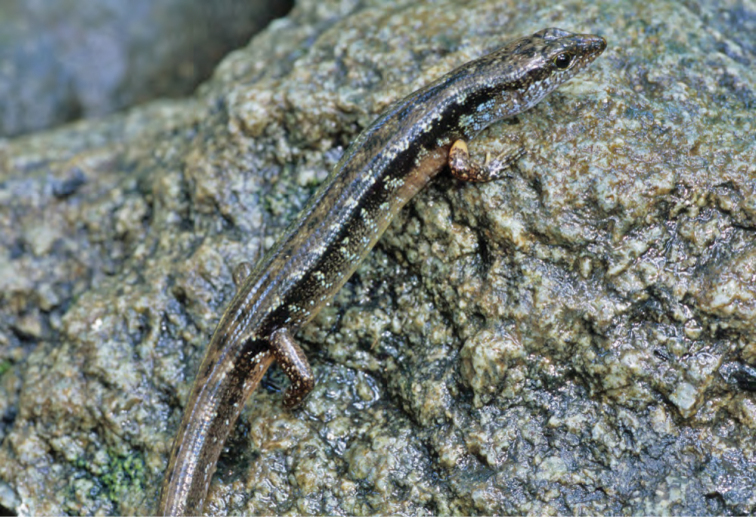
*Parvoscincus leucospilos* (KU 327785) from Barangay Dibuluan, San Mariano (Location 30). Photo: ACD.

Yet another species recently transferred from the paraphyletic genus *Sphenomorphus* on the basis of an extensive phylogenetic analysis and a survey of morphological characters (Linkem et al. 2011), *Parvoscincus leucospilos* (Fig. 67) is a semiaquatic species that has escaped detection in the Philippines since its original description (review: Taylor 1922b; Brown and Alcala 1980) until Brown et al. (2000a) discovered a single specimen in Aurora Province in 1997. Since that time we have found numerous populations of this enigmatic species by focusing on riparian streams in intact forest between 200 and 800 m (Brown et al. 2000a, 2012; Siler et al. 2011; McLeod et al. 2011). Our sighting of this species at 600 m on Mt. Cagua resulted from disturbing the nocturnal resting place of a specimen (under stream side wet leaf litter); following disturbance, the species dove into the nearby running water and escaped capture, a strategy typical for this ecologically unique taxon (RMB, ACD, CDS, *personal observation*).

Cagayan Province—Location 1b: no specimens (RMB and J. E. Fernandez field observation).

Isabela Province—Location 23: KU 320522; Location 24: KU 327785–86; Location 25: KU 327787–96.

***Parvoscincus steerei* (Stejneger, 1908)**

Considered a widespread, variable Philippine endemic, *Parvoscincus steerei*, like its congener, *Parvoscincus leucospilos*, has been transferred from the paraphyletic genus *Sphenomorphus* on the basis of a robust phylogenetic analysis and a survey of new morphological characters (Linkem et al. 2011); an ongoing phylogeographic study, coupled with a review of its conservative morphology may result in taxonomic partitioning once sufficient data have been accumulated. Past studies have noted considerable color pattern and body size variation in this small species of Philippine skink (Taylor 1922b; Brown et al. 1996, 2000a, 2012; Brown and Alcala 1980; Siler et al. 2011). This species is common across a wide range of habitats and elevational gradients but is most frequently encountered under leaf litter and woody debris in riparian habitats between 300 and 900 m above sea level.

Cagayan Province—Location 1a: KU 330107–17; Location 1b: KU 330118; Location 5: PNM 5373–74; Location 6: USNM 498626-29; Location 15: USNM 498914; Location 16: USNM 498913.

Isabela Province—Location 23: PNM 673.

***Parvoscincus tagapayo* (Brown, McGuire, Ferner & Alcala 1999)**

Discovered only in 1999 (Brown et al. 1999, 2000a) and originally considered rare and limited to mid-elevation primary forest, this species is now known to occur in a variety of habitat types in Kalinga, Apayao, Isabela, Nueva Viscaya, Aurora, Ilocos Norte (Brown et al. 2012), and now Cagayan Provinces. This mid-to high-elevation forest obligate qualifies as “Vulnerable” (VU: B1ab(iii)) because it is known only from a limited (probably less than 20,000 km^2^, but more likely more than 5,000 km^2^) extent of occurrence and fragmented habitat type, with habitats (low-to mid-montane dry forests) likely to decline in quality and possibly in extent. Future re-evaluations based on field surveys of actual populations will be necessary to confidently establish the conservation status of this species.

Cagayan Province—Location 2b: KU 330059–60.

### Family Varanidae

***Varanus marmoratus* (Wiegmann, 1834)**

**Figure 68.**
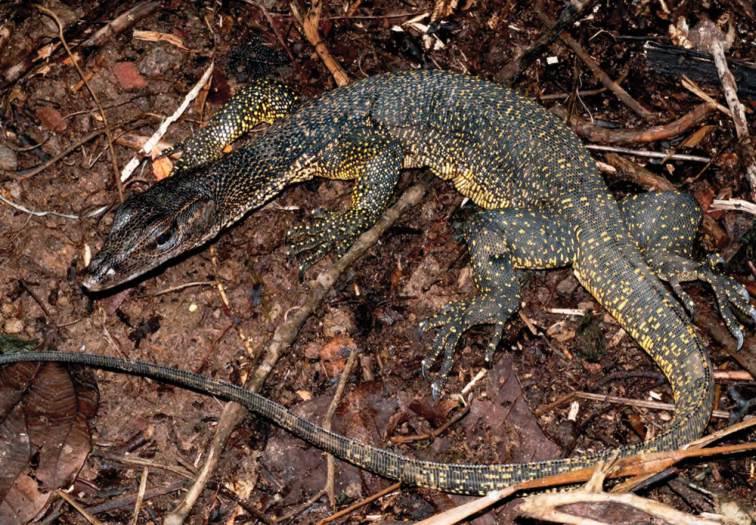
*Varanus marmoratus* (KU 330731) from low-elevation, Mt. Cagua, below Location 1b, near Barangay Magrafil. Photo: RMB.

This Luzon faunal region (Brown and Diesmos 2002, 2009) monitor lizard (Fig. 68) is ubiquitously present in low elevation habitats, including completely denuded coastal areas, agricultural plantations, scrubby vegetation, matrices of secondary growth and primary forest patches, and along forest edges from low- to mid-elevations (Gaulke 1991a, 1991b, 1992a, 1992b). Frequently observed scavenging around human habitats, and heavily disturbed riparian habitats, this species appears to have benefitted from the activities of humans in the northern Sierra Madre. That said, exploitation of this species (for food, leather and pets) is pronounced (Gaulke et al. 1992b, 1998; Brown et al. 2002; Welton et al. 2012), and we have frequently observed this species offered for sale by residents at bush meat stands along the major highways of Cagayan Valley.

Cagayan Province—Location 1a: KU 330729; Location 3: KU 326697; Location 5: USNM 305884; Location 13: PNM 5475; Location 15: USNM 498915–17; Location 19: PNM 5989.

Isabela Province—Location 23: PNM 683.

***Varanus bitatawa* Welton, Siler, Benett, Diesmos, Duya, Dugay, Rico, van Weerd & Brown 2010**

**Figure 69.**
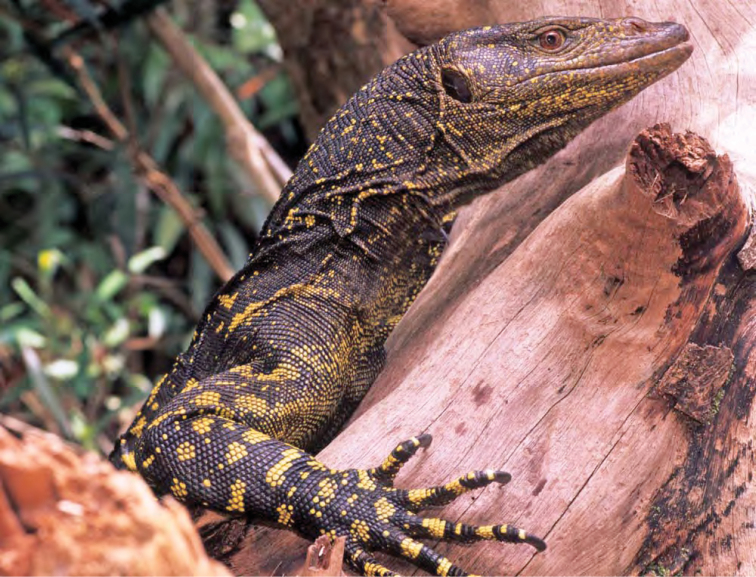
One of the firstphotographs in life of the newly discovered (Welton et al. 2010) *Varanus bitatawa* (KU 322188) from Barangay Dibuluan, San Mariano (Location 23). Photo: ACD.

**Figure 70.**
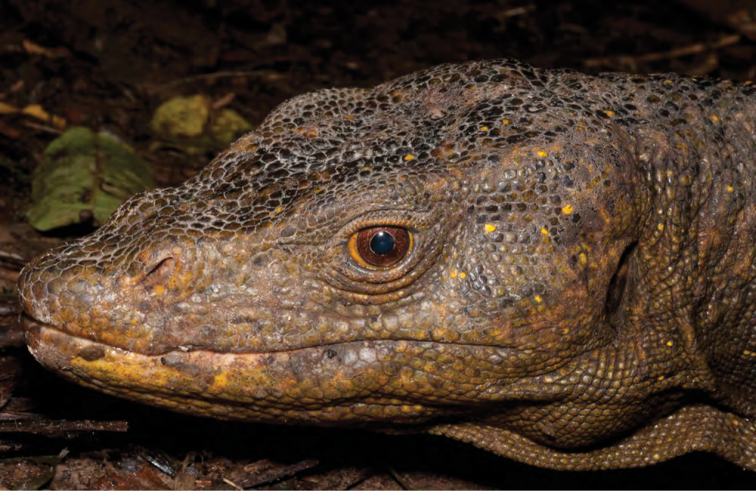
A large (nearly 2 m) adult male *Varanus bitatawa* in life, near Location 2. Photo: RMB.

**Figure 71.**
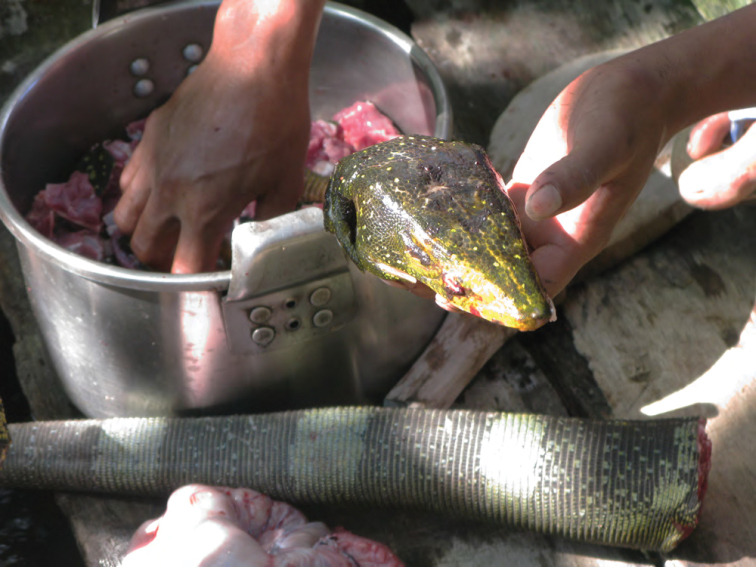
*Varanus bitatawa* (skull and partial specimen salvaged: KU 330636) stew being prepared at Location 2 by Agta tribesmen. Photo: RMB.

This distinctive species of arboreal, frugivorous, large-bodied monitor lizard (Figs 69, 70) was discovered by scientists during the past decade but not described until 2010 (holotype PNM 9719 from Aurora Province); the species was previously well known to Agta tribes peoples (Estioko–Griffin and Griffin 1975; Griffin and Estioko–Griffin 1985) who consider it a choice delicacy and recognize it with a distinctive local name (“*Bitatawa*;” Welton et al. 2010). *Varanus bitatawa* appears to be widespread and common in forested regions of the northern Sierra Madre (Welton et al. 2010; 2012), extending as far south as the Lingayen-Dingalan geologic fault (Defant et al. 1989; Yumul et al. 2003) and three low-lying, arid river valleys constituting the Mid-Sierra Filter Zone (Welton et al. 2010). This hypothesized barrier divides northern Aurora Province from southern Isabela Province (Fig. 71) and may have served as an ecological or physical barrier to dispersal, possibly promoting divergence between *Varanus bitatawa* and its closest relative, *Varanus olivaceus*, (Auffenberg 1976, 1979, 1988) from Bulacan and Quezon Provinces, Polillo, and Catanduañes islands, and the Bicol faunal region. Our new records from barangays Magrafil and Santa Clara (Figs 69, 70), Municipality of Gonzaga, are the northernmost records for this species (Welton et al. 2012). Residents and wildlife managers in the vicinity of Gonzaga report that *Varanus bitatawa* is a prized target for local consumption in bush meat trade (Fig. 70), and is targeted, in particular, by Agta tribal groups who heavily hunt this species for its meat, preferring it to the more common *Varanus marmoratus* (Welton et al. 2012). Although a recent survey found conspicuous signs of arboreal monitors in the northern Cordillera Mountains (Brown et al. 2012), to date *Varanus bitatawa* appears to be restricted to the northern Sierra Madre where it is abundant, frequently encountered by hunters, and heavily hunted for bush meat.

Cagayan Province—Location 1b: KU 330730; Location 2: KU 330636, 330731.

Isabela Province—Location 23: KU 322188 (paratype); Exact locality unknown: KU 327100.

### Reptilia: Snakes. Family Pythonidae

***Broghammerus reticulatus* (Schneider 1801)**

**Figure 72.**
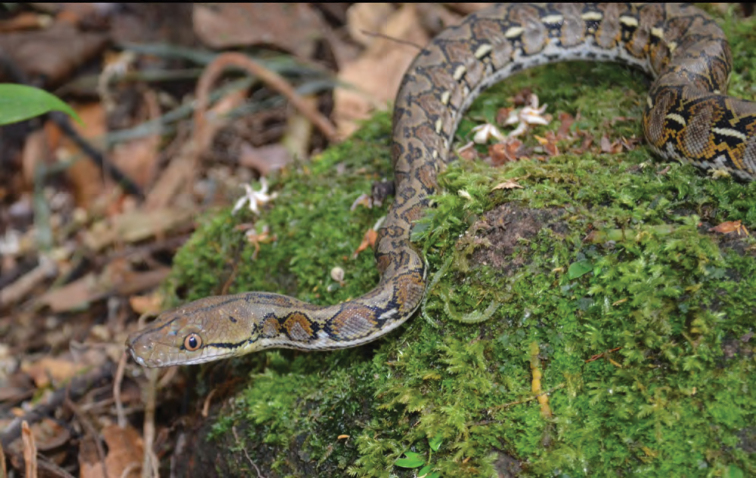
Subadult male*Broghammerus reticulatus* (KU 330021) at Location 1a. Photo: JS.

Reticulated pythons (Fig. 72) are common in a wide variety of low- to mid-elevation habitats, including residential areas, agricultural plantations, and the slash-and-burn shifting disturbed forests typical of the foothills of major Sierra Madre mountain slopes. Also hunted for meat and leather (Gaulke 1998), this species may require additional measures of protection if commercial exploitation becomes prominent in Isabela and/or Cagayan Provinces. Our specimens were collected in riparian habitats where they were actively hunting on the ground and in low vegetation strata at night.

Cagayan Province—Location 1a: KU 330021; Location 15: USNM 498919, 499259; Location 17: USNM 498918.

Isabela Province—Location 21: PNM 9157.

### Family Colubridae

***Ahaetulla prasina preocularis* (Taylor, 1922)**

**Figure 73.**
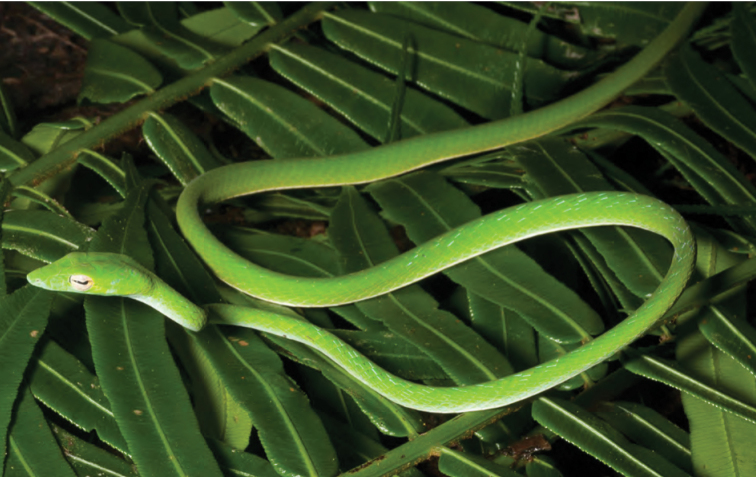
*Ahaetulla prasina preocularis* (KU 330037) green morph from Location 1b. Photo: RMB.

**Figure 74.**
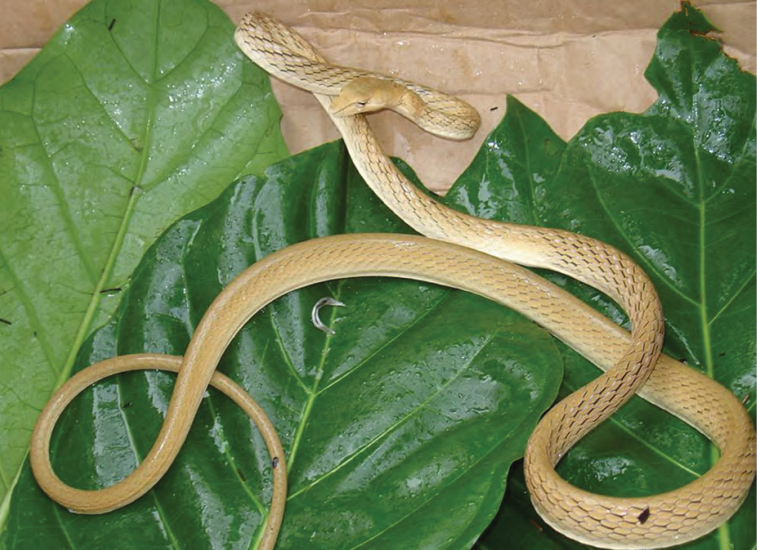
*Ahaetulla prasina preocularis* (specimen not collected) yellow morph from Palanan (Location 35). Photo: MVW.

Widely distributed throughout the Philippines (Leviton 1963a, 1967), this species (Figs 73, 74) is most often encountered sleeping on branches of bushes and saplings in secondary growth and selectively logged primary growth forest, on the edges of agricultural plantations, and in shrubs surrounding residential areas.

Cagayan Province—Location 1a: KU 330032–33; Location 1b: KU 330034–37; Location 3: KU 307433; Location 15: USNM 498920; Location 16: USNM 498921.

Isabela Province—Location 21: PNM 241; Location 23: KU 327171; Location 26: KU 327172; Location 30: PNM 254; Location 32: no specimens (MVW photo voucher); Location 35: no specimens (MVW photo voucher).

***Boiga cynodon* (Boie, 1827)**

**Figure 75.**
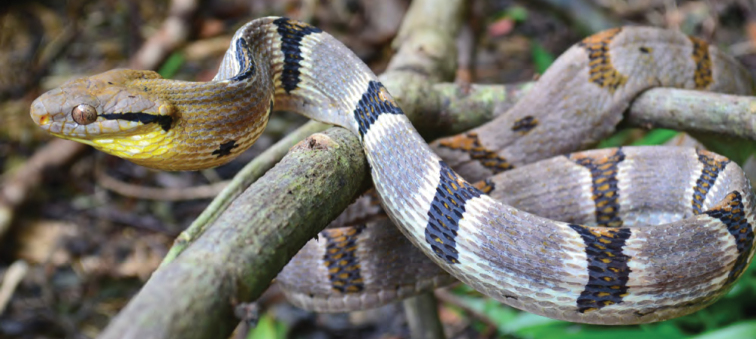
*Boiga cynodon* (KU 330586) from mid-elevation, Mt. Cagua, Location 1b. Photo: LJW..

Widely distributed in Southeast Asia (Leviton 1970), *Boiga cynodon* (Fig. 75) is highly variable in color pattern, ranging from blond to tan and patternless, to gray with brown and black irregular transverse saddles. We collected two specimens away from water, actively foraging in understory vegetation in a mixture of secondary growth and selectively logged primary growth forest (on the lower slopes of Mt. Cagua); several specimens were captured in low-elevation forest patches at Gattaran in stream-side understory vegetation.

Cagayan Province—Location 1b: KU 330586–87; Location 3: KU 327774, 327777.

Isabela Province—Location 24: KU 327773.

***Boiga dendrophila divergens* Taylor, 1922**

**Figure 76.**
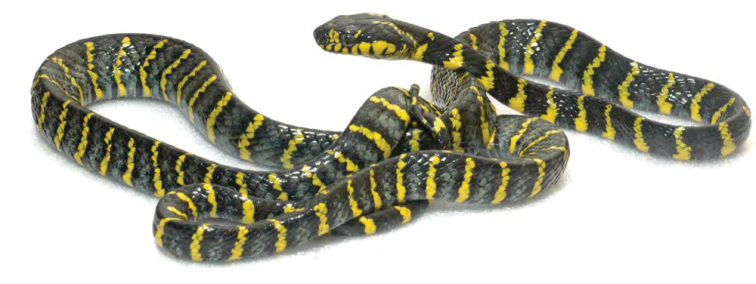
*Boiga dendrophila divergens* (PNM 969) from Santa Ana (Location 12). Photo: ACD.

One specimen of this widespread endemic Luzon subspecies (Fig. 76) has been collected at the Municipality of Santa Ana (circumstances of collection unknown). This species is undoubtedly widespread throughout low elevation and coastal habitats of northeastern Luzon, and its presence has been confirmed in the Babuyan Islands off the northeast tip of Luzon as well (Oliveros et al. 2011).

Cagayan Province—Location 12: PNM 969.

***Boiga philippina* (Peters, 1867)**

**Figure 77.**
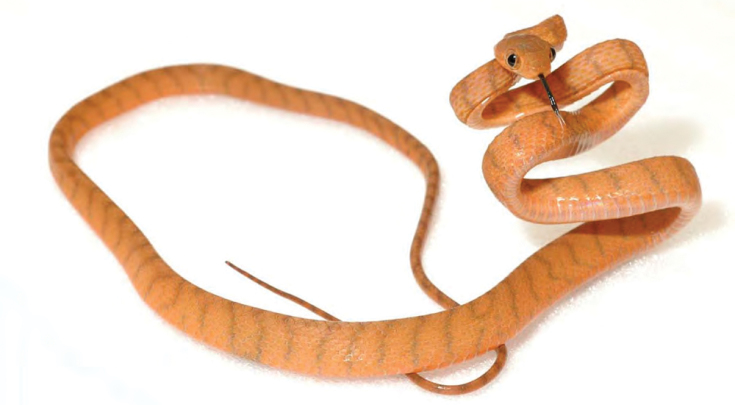
*Boiga philippina* (KU 307435) from Barangay Nassiping, Gattaran (Location 3). Photo: ACD.

A single specimen was collected in the Nassiping Forest Reserve where it was active at night in lower branches of streamside understory vegetation. Also now known from the Babuyan Islands (Oliveros et al. 2011), this species (Fig. 77) appears to be a northern Philippine taxon, in accordance with its reported type locality (“northwestern Luzon;” Peters 1867).

Cagayan Province—Location 3: KU 307435.

***Calamaria bitorques* Peters 1872**

**Figure 78.**
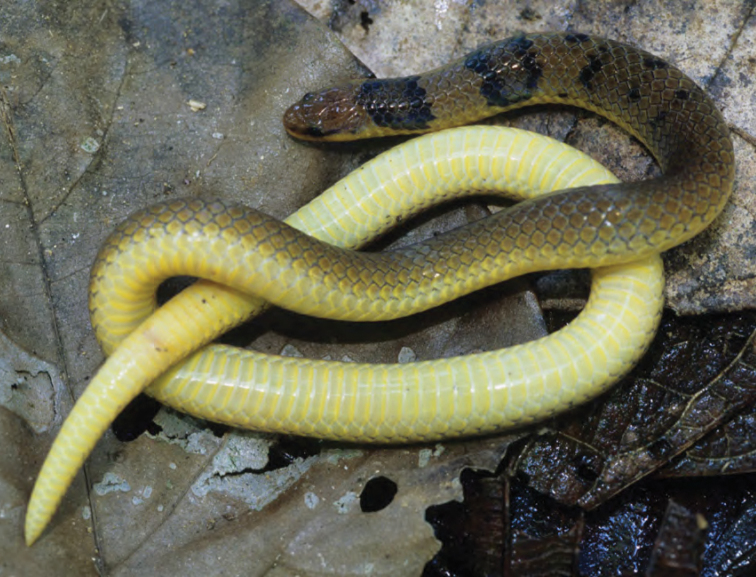
*Calamaria bitorques* (KU 327409) from Barangay Dibuluan, San Mariano (Location 31). Photo: ACD.

Previously collected in the southern Sierra Madre (Aurora Province; Brown et al. 2000a; Siler et al. 2011), this species (Fig. 78) is infrequently encountered but can be distinguished from *Calamaria gervaisi* on the basis of color pattern and larger maximum body size (Inger and Marx 1965).

Cagayan Province—Location 1b: PNM 8475; Location 15: USNM 498922; Location 20: PNM 8273.

Isabela Province—Location 23: KU 327409–10.

***Calamaria gervaisi* Duméril & Bibron, 1854**

**Figure 79.**
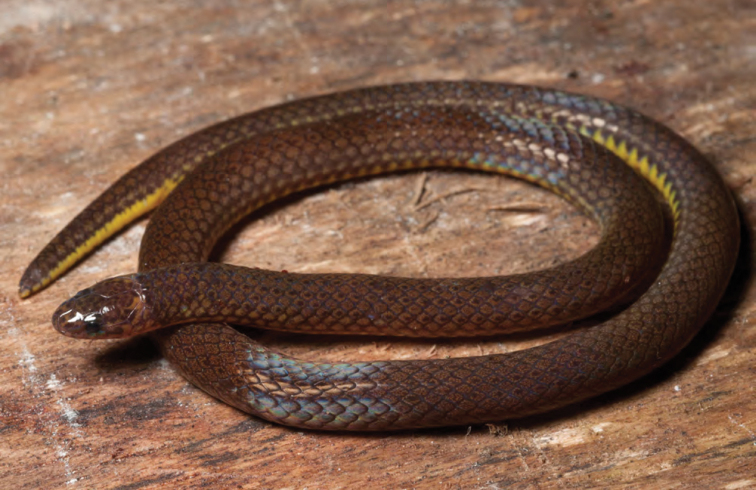
*Calamaria gervaisi* (KU 330084) from 1000, Mt. Cagua, near Location 1a. Photo: RMB.

Widely distributed in the Philippines (Inger and Marx 1965), this species (Fig. 79) has been recorded at numerous sites throughout Luzon (Brown et al. 1996, 2000a, 2012; Diesmos et al. 2005; Siler et al. 2011; McLeod et al. 2011).

Cagayan Province—Location 1b: KU 330081–85.

Isabela Province—Location 21: KU 307441, PNM 102; Location 23: KU 326693; Location 26: KU 327406–07; Location 30: PNM 126.

***Coelognathus erythrurusmanillensis* (Jan, 1863)**

**Figure 80.**
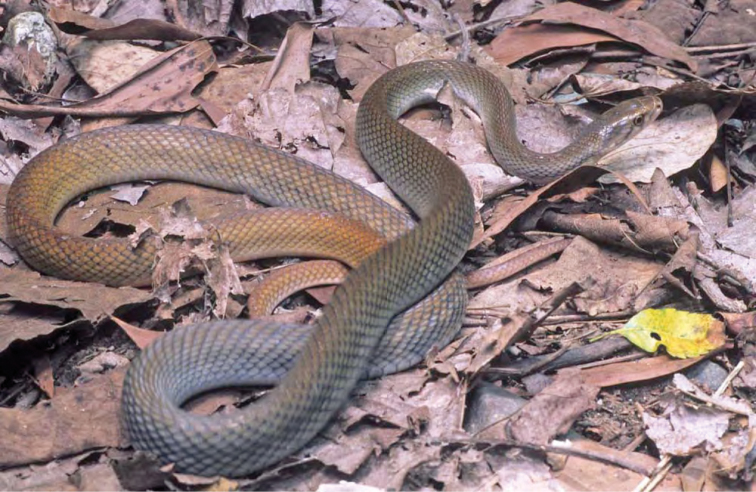
*Coelognathus erythrurus manillensis* (uncataloged specimen in PNM) from Barangay Binatug (Location 22). Photo: ACD.

We encountered this Luzon endemic (Leviton 1979; Fig. 80) actively foraging (in the late morning) on the ground in dry forest at low elevations in Cagayan Province in the Nassiping Forest Reserve.

Cagayan Province—Location 3: KU 307468; Location 10: USNM 498631–33; Location 19: PNM 388.

Isabela Province—Location 24: uncataloged specimen in PNM; Location 30: no specimens (MVW photo voucher).

***Cyclocorus lineatus lineatus* (Reinhardt, 1843)**

**Figure 81.**
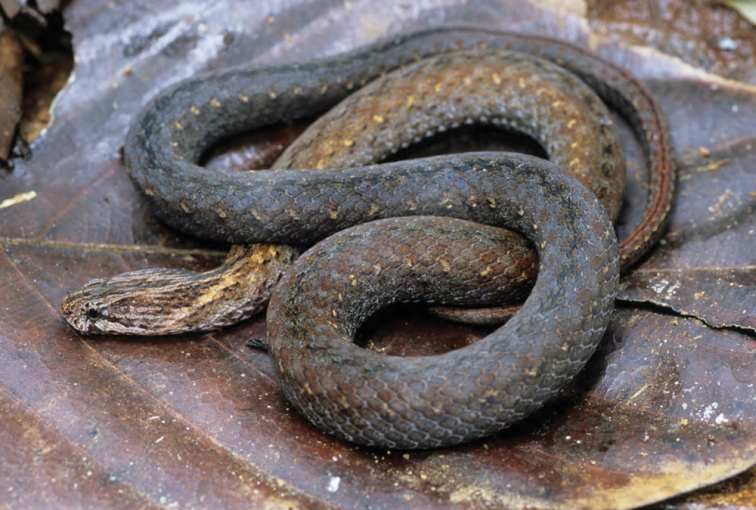
*Cyclocorus lineatus lineatus* (KU 326690) from Barangay Dibuluan, San Mariano (Location 30). Photo: ACD.

One of only four snake genera endemic to the Philippines (Taylor 1922c; Leviton 1963a), Luzon populations of *Cyclocorus lineatus* (Leviton 1965a; Fig. 81) have commonly been encountered by us under the cover of rocks, loose soil, logs, and other debris along banks of streams and rivers (*personal observations*). An additional specimen was collected from within soft, dry rot decaying wood matter in a large stump on the bank of a stream at 600 m on Mt. Cagua.

Cagayan Province—Location 1a: 330024–27; Location 1b: 330028–29, PNM 8477; Location 2: KU 320511; Location 5: USNM 305879–82; Location 6: USNM 498630; Location 15: USNM 498923–25, 292494; Location 18: USNM 292495.

Isabela Province—Location 23: KU 326689–90, 327755; Location 24: KU 327756–59; Location 26: KU 327760–61; Location 28: KU 327762–64; Location 34: no specimens (MVW photo voucher).

***Dendrelaphis luzonensis*Leviton 1961**

**Figure 82.**
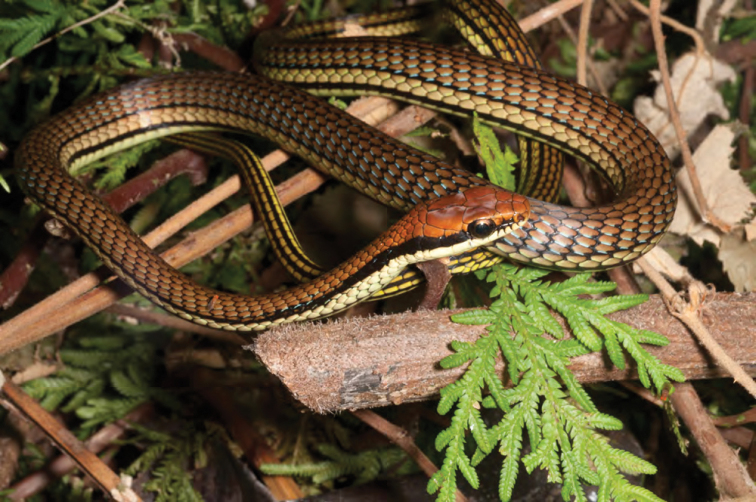
*Dendrelaphis luzonensis* (KU 330030) from mid-elevation, Mt. Cagua, Location 1b. Photo: RMB.

Luzon populations of *Dendrelaphis luzonensis* (Leviton 1961, 1968; van Rooijen and Vogel 2012; Fig. 82) are most often encountered asleep in shrubbery and understory vegetation, ferns and palms, surrounding the banks of streams and rivers, especially at low elevations in and around agricultural areas and on forest edges. We collected a specimen on the lower slopes of Mt. Cagua sleeping in vines clinging to a large tree trunk in selectively logged forest away from water.

Cagayan Province—Location 1b: KU 330030; Location 5: USNM 498636, 499013, CAS 116192; Location 11: PNM 474; Location 17: USNM 498926; Location 19: PNM 458, 461.

Isabela Province—Location 21: PNM 9146, 9189, 9190.

***Dendrelaphis marenae* Vogel & van Rooijen, 2008**

**Figure 83.**
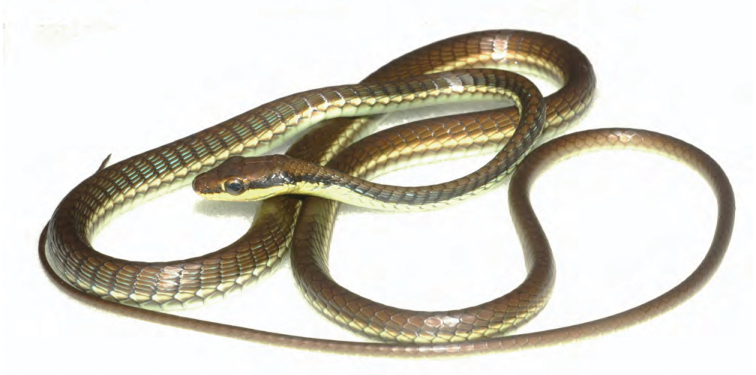
*Dendrelaphis marenae* (uncataloged ACD specimen in PNM). Photo: ACD.

Common in residential and agricultural areas where they are most often seen active during the day on the ground or sleeping in bushes at night, this common species is widely distributed throughout the northern Philippines (Leviton 1968). *Dendrelaphis marenae* (Fig. 83) has recently been morphologically distinguished and diagnosed as a species distinct from the Indochinese *Dendrelaphis pictus*, and the eastern Indonesian *Dendrelaphis grismeri* (Vogel and van Rooijen 2008).

Cagayan Province—Location 5: USNM 498637; CA 116193; Location 10; USNM 498634; Location 13: PNM 8410.

Isabela Province—Location 21: PNM 9163; Location 33: no specimens (MVW photo voucher); Location 36: PNM 542.

***Dryophiops philippina* Boulenger, 1896**

**Figure 84.**
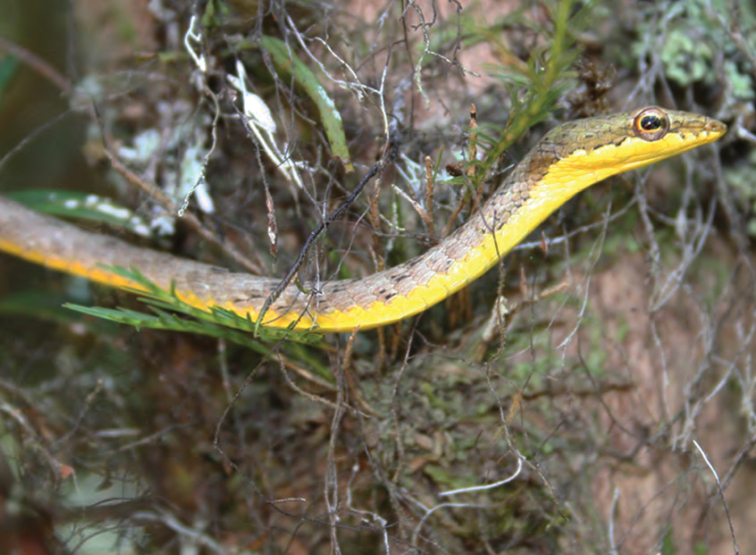
*Dryophiops philippina* (specimen not collected) from Pangden (Location 33). Photo: MVW.

This widespread Philippine endemic (Fig. 84) has been collected in recent years with increasing frequency as workers target the remaining low-elevation and coastal forests of the Philippines. We observed one specimen in ultrabasic forests near the municipality of Palanan.

Isabela Province—Location 32: no specimens (MVW photo voucher).

***Gonyosoma oxycephalum* (Boie, 1827)**

**Figure 85.**
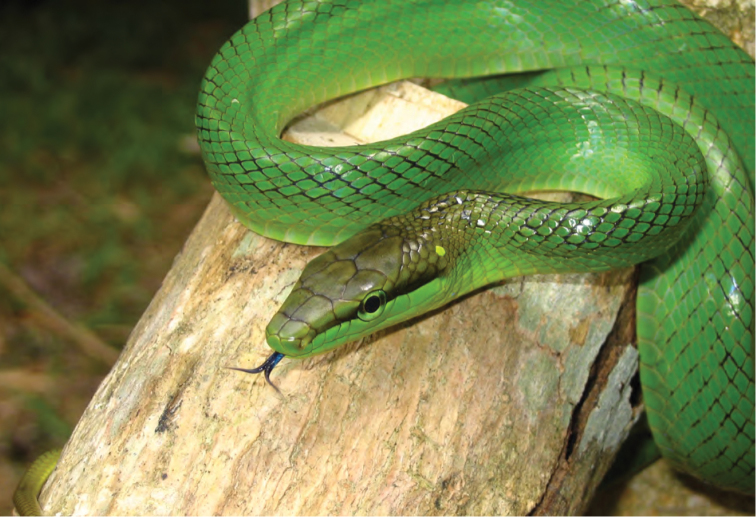
*Gonyosoma oxycephalum* (ACD 3091) from Barangay Binatug (Location 22). Photo: K. M. Hesed.

This widespread, non-endemic, Southeast Asian rat snake (Fig. 85) has been documented throughout the Philippines in a wide variety of habitats. Our records originated in forested areas at low elevations in Binatug and Palanan.

Isabela Province—Location 22: uncataloged specimen deposited in PNM (K. Hesed photo voucher); Location 30: no specimens (MVW photo voucher); Location 32: PNM 2031.

***Hologerrhum philippinum* Günther, 1858**

**Figure 86.**
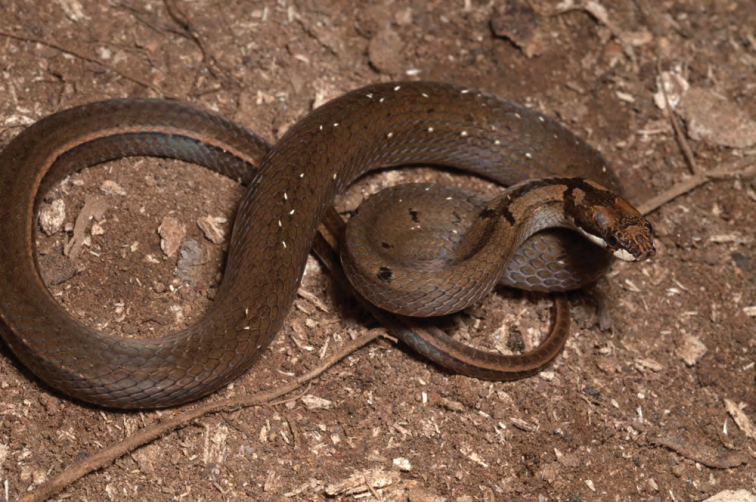
*Hologerrhum philippinum* (KU 330056) from 650 m asl, Mt. Cagua, Location 1b. Photo: RMB.

**Figure 87.**
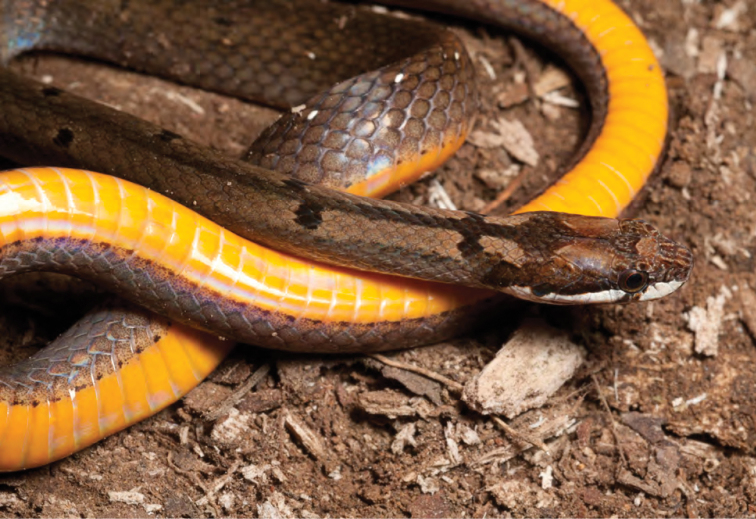
Closeup of *Hologerrhum philippinum* (KU 33056) illustrating details of head scalation and color, and vibrant yellow ventral coloration. Photo: RMB.

Seldom encountered, the genus *Hologerrhum* is one of only four snake genera endemic to the Philippines (Taylor 1922c; Leviton 1963a, 1983; Taylor 1963). Collected only a few times in the past two decades (Brown et al. 1996, 2001; McLeod et al. 2011; Phenix et al. 2011), this species has been encountered in recent years in dry forest among bamboo stands (ACD, *personal observation*) and multiple times in dry streambeds under rocks. Our specimen from Mt. Cagua (Figs 86, 87) was collected in a dry ravine, under a small rock, at 600 m in selective logged primary forest. In contrast to other recent specimens with salmon-red or pinkish red ventral surfaces, our Mt. Cagua specimen had a bright yellow venter. The only other species in the genus (*Hologerrhum dermali* from Panay Island; Ferner et al. 2001; Gaulke 2001) also has a bright yellow venter but differs from *Hologerrhum philippinum* by the presence of a mid-ventral black stripe (Brown et al. 2001).

Cagayan Province—Location 1b: KU 330056, PNM 8480; Location 13: USNM 498718.

Isabela Province—Location 36: PNM 6505.

***Lycodon capucinus* (Boie, 1827)**

**Figure 88.**
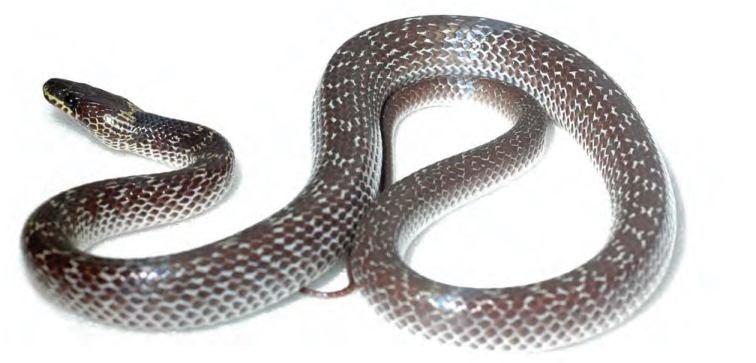
*Lycodon capucinus* (uncataloged ACD specimen in PNM). Photo: ACD.

Widespread and common throughout the Philippines and Southeast Asia (Leviton 1963a, 1965b; Manthey and Grossman 1997; Inger and Voris 2001), *Lycodon capucinus* (Fig. 88) is a frequently encountered snake in residential and agricultural areas at low elevations. We collected one road kill specimen on the road to Barangay Magrafil along the north coast Luzon highway in the Municipality of Gonzaga. The recent phylogenetic study of Siler et al. (2013) revealed moderate levels of genetic diversity among sampled populations of *Lycodon capucinus* across its recognized range in Southeast Asia. The species relationship with the morphologically similar species *Lycodon aulicus* (Linnaeus 1758) has long been controversial (review: Siler et al. in press b), and future studies focused on the *Lycodon aulicus* and *Lycodon capucinus* will be needed to fully resolve species boundaries within this widespread species complex.

Cagayan Province—Location 1b: Uncataloged specimen at KU (RMB 15097).

***Lycodon muelleri* Duméril, Bibron & Duméril, 1854**

**Figure 89.**
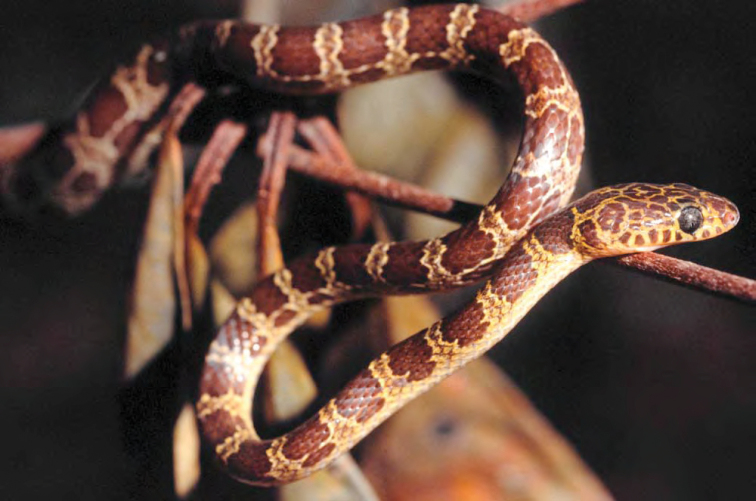
Typical appearanceof *Lycodon muelleri* (PNM 6592) from Barangay Binatug (Location 22). Photo: ACD.

*Lycodon muelleri* (Fig 89) has been recorded throughout Luzon (Leviton 1965b; Brown et al. 2000a; Siler et al. 2011) and is frequently collected in low vegetation along streams and rivers at low elevations (<500 m). Our Isabela Province specimen was collected in the Municipality of San Mariano, at night, on the buttress of a large tree adjacent to a mountain stream. In a recent phylogenetic study of Southeast Asian wolf snakes, a deep genetic divergence was observed between populations of *Lycodon muelleri* from northern and central Luzon and populations sampled on the Bicol Peninsula in southeast Luzon (Siler et al. 2013).

Isabela Province—Location 26: KU 327573; Location 35: no specimens (MVW photo voucher); Location 36: PNM 6592.

***Lycodon solivagus* Ota and Ross, 1984**

A single specimen of this species was collected close to the type locality (Ota and Ross 1994) where it was found dead on the road between Barrio Battalan and Lasam Centro (Municipality of Lasam); this damaged specimen was identified by its distinctive dentition. The only other specimen ever collected is a single individual from Barangay Paitan, Municipality of Quezon, Nueva Vizcaya Province (KU 325974). Unfortunately, tissue samples of this species presently are not available, and how this species is related to the many other Philippine endemic wolf snakes remains undetermined (Siler et al. 2013).

Cagayan Province—Location 5: USNM 499014, PNM 2046.

***Oligodon ancorus* (Girard, 1858)**

Leviton (1962) listed sites for this species throughout much of Luzon. The one specimen recently collected in Cagayan province was encountered as it rested on top of a rock by a small stream.

Cagayan Province—Location 15: USNM 498927.

***Psammodynastes pulverulentus* (Boie, 1827)**

**Figure 90.**
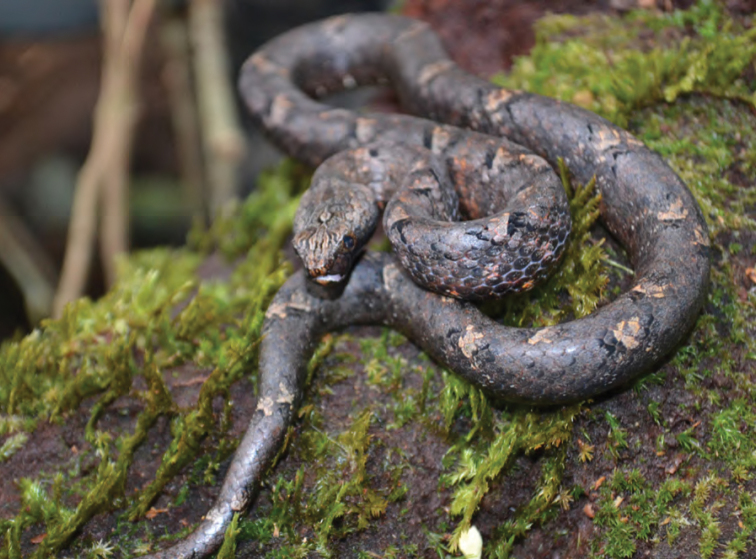
*Psammodynastes pulverulentus* from Location 1a (KU 330023). Photo: JS.

This common, widespread species (Manthey and Grossman 1997; Inger and Voris 2001; Fig. 90) has been documented throughout Luzon (Leviton 1983; Brown et al. 2000a, 2012; Siler et al. 2011; McLeod et al. 2011); one of our specimens was collected on the ground on a stream bank at night and the other was encountered asleep among the lower branches of a small bush on a river bank.

Cagayan Province—Location 1a: KU 330023; Location 1b: KU 330022, PNM 8482; Location 13: PNM 8399.

Isabela Province—Location 30: CAS 15320.

***Pseudorhabdion* cf. *mcnamarae* (Taylor 1917)**

**Figure 91.**
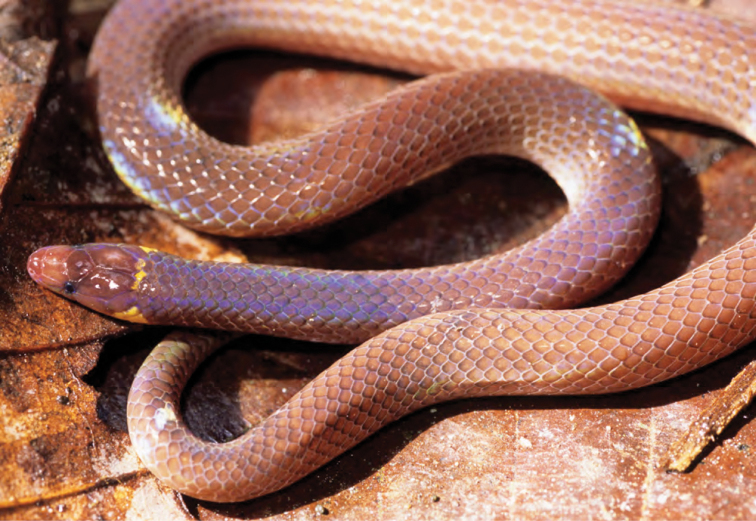
*Pseudorhabdion* cf. *mcnamarae* (KU 327193) from Barangay Binatug (Location 22). Photo: ACD.

*Pseudorhabdion mcnamarae*was originally described from northern Negros (Visayan island group; Taylor 1917, 1922a; Leviton and Brown 1959), but at least one specimen from Luzon has ostensibly been referred to this species (CAS 61544, from Balbalan, Kalinga Province). Although we suspect that *Pseudorhabdion mcnamarae* is actually restricted to the Visayan faunal region (Brown et al. 1999; Gaulke 2001; Ferner et al. 2001) and that the Luzon population (Fig 91) represents another, possibly undescribed, species, we do not recommend taxonomic action until a detailed study of this group can be undertaken.

Isabela Province—Location 22: KU 327206; Location 23: KU 326694–95; Location 26: KU 327189–205.

***Pseudorhabdion* cf. *talonuran* Brown, Leviton & Sison, 1999**

Another specimen of a distinctive species of *Pseudorhabion* has been collected in the Dibanti River basin, Barangay Dibuluan (Municipality of San Mariano). This specimen most closely resembles *Pseudorhabdion talonuran*, a high-elevation species from Panay Island (Brown et al. 1999; Ferner et al. 2001), suggesting a biogeographically improbable, disjunct distribution and the possibility that this single specimen may constitute the first record of a new species from the northern Sierra Madre.

Isabela Province—Location 26: KU 327216.

***Ptyas luzonensis* (Günther, 1873)**

**Figure 92.**
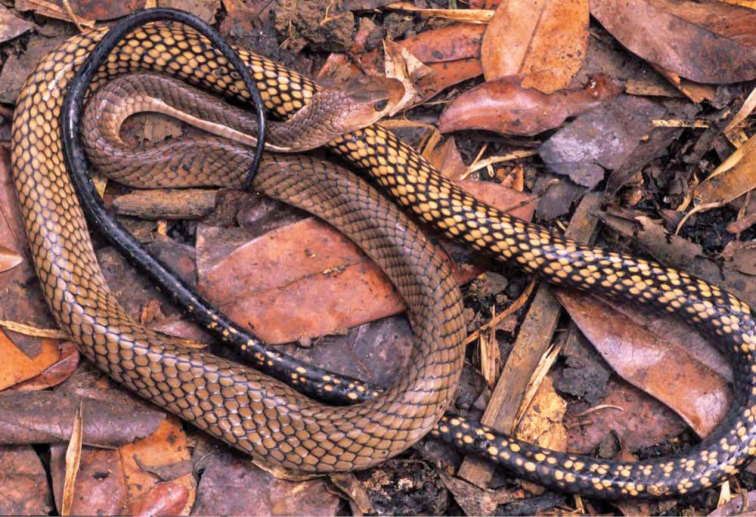
*Ptyas luzonensis* (uncataloged specimen in PNM) from Barangay Dibuluan, San Mariano (Location 31). Photo: ACD.

Now considered common and widespread throughout the Luzon and Visayan faunal regions (Leviton 1983; Ross et al. 1987), this species (Fig. 92) has been documented throughout Luzon at a variety of forested sites (Brown et al. 2012; Siler et al. 2011; McLeod et al. 2011). Whereas most recent specimens have been encountered at night, asleep in branches of understory trees, along the banks of streams in selectively logged primary and secondary growth forests (Siler et al. 2011; Brown et al. 2012; McLeod et al. 2011), the one Cagayan Province record was collected on the ground where it was actively hunting at night along a dry ridge.

Cagayan Province—Location 15: USNM 498931.

***Rhabdophis spilogaster* (Boie, 1827)**

**Figure 93.**
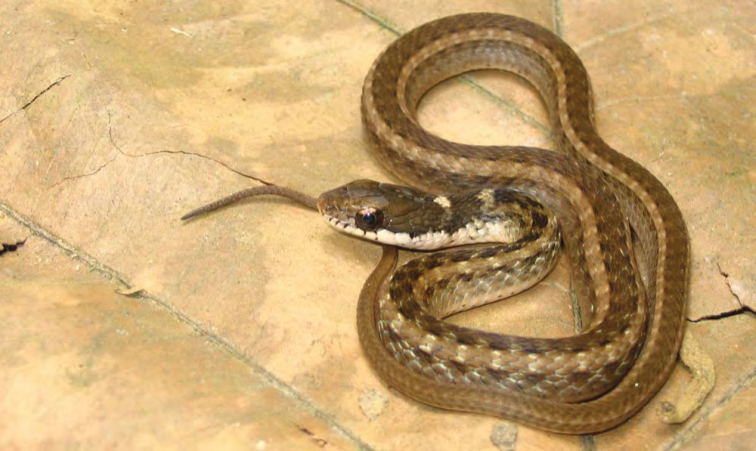
*Rhabdophis spilogaster* (KU 327287) from Barangay Dibuluan, San Mariano (Location 23). Photo: K. M. Hesed.

*Rhabdophis spilogaster* (Fig. 93) is diurnally active in riparian habitats at low- to mid-elevations throughout Luzon. Specimens were collected in artificial fish ponds in residential areas, swimming in side pools of small streams, along the borders of flooded rice fields, and among rocks on stream banks in regenerating secondary growth forest.

Cagayan Province—Location 5: USNM 498635; Location 12: PNM 4094; Location 13: PNM 4093; Location 15: USNM 498928–30.

Isabela Province—Location 21: PNM 4106; Location 23: KU 327287–89, PNM 4060; Location 26: KU 327290–94.

***Tropidonophis dendrophiops* (Günther, 1883)**

**Figure 94.**
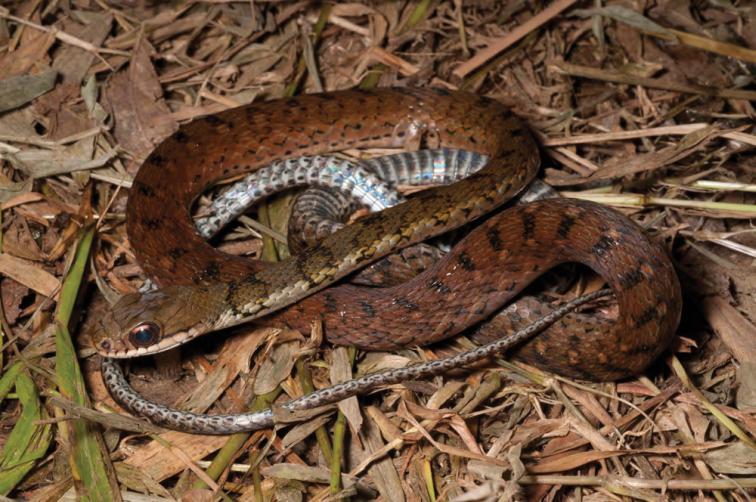
*Tropidonophis dendrophiops* (KU 330031) from mid-elevation, Mt. Cagua, Location 1b. Photo: RMB.

This moderately common natricine snake (Fig. 94) is an ecological generalist that is frequently encountered in riparian habitats (Leviton 1963a; Malnate and Underwood 1988). Our specimens were collected during mid- to late morning when they were active in nearly dry streambeds; specimens were first observed actively crawling among rocks and other debris.

Cagayan Province—Location 1b: KU 330031, PNM 8481.

Isabela Province—Location 23: KU 327620–21.

### Family Elapidae

***Hemibungarus calligastercalligaster* (Wiegmann, 1835)**

**Figure 95.**
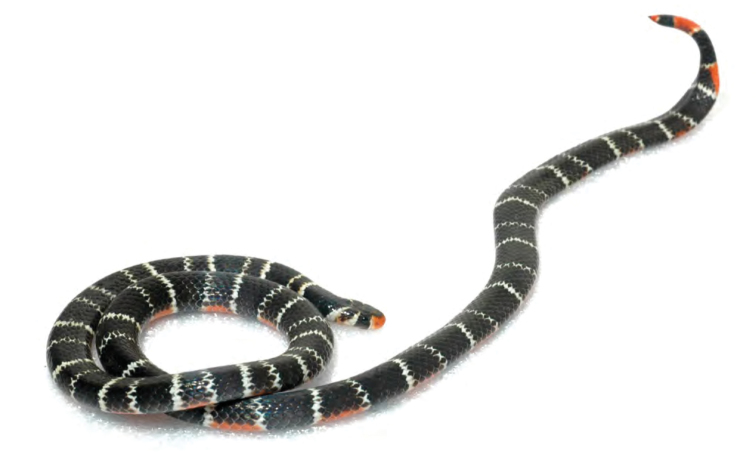
*Hemibungarus calligaster calligaster* (uncataloged ACD specimen in PNM). Photo: ACD.

A single specimen of this widespread coral snake (Leviton 1963b; Brown 2006; Siler and Welton 2010; Siler et al. 2011; Fig. 95) was collected in a forest fragment in Santa Ana. It is widespread throughout the eastern seaboard of Luzon but has not yet been documented in the Cordillera (Diesmos et al. 2005; Brown et al. 2012).

Isabela Province—Location 21: PNM 6607.

***Naja philippinensis* Taylor 1922**

A single specimen of the distinctive Philippine cobra (Leviton et al. 1964b) was collected at Barangay San Pedro, Municipality of Lasam. Recent encounters with this species in Aurora Province (Siler et al. 2011) plus historical records (Leviton et al. 1964b) suggest that it is widespread and common throughout the Sierra Madre. Persecution and exploitation of this species have been identified as potential conservation threats (Gaulke 1998, Brown et al. 2002; IUCN 2011). Circumstances of capture of the single documented Cagayan Province specimen are unclear; the animal was most likely captured by residents in agricultural areas surrounding human settlements (*personal observations*).

Cagayan Province—Location 10: USNM292493.

***Ophiophagus hannah* (Cantor 1936)**

Residents of San Mariano and Palanan related to us numerous instances of sightings and resident killings of very large, light tan-colored cobras in the vicinity of settlements and agricultural areas. This species has been reported widely on Luzon (Leviton 1964b; McLeod et al. 2011; Siler et al. 2011; Devan-Song and Brown 2012) and is known to the residents of Isabela as well. One of us (ACD) sighted an additional king cobra in a forest fragment at Apaya (Location 23). Thus, our own records plus resident reports provide, in our opinion, sufficient credibility to include *Ophiophagus hannah* in this report, although we consider these records unconfirmed until voucher specimens become available.

Isabela Province—Locations 22 and 29: no specimens (ACD field identification).

### Family Homalopsidae

***Cerberus schneideri* (Schlegel, 1837)**

**Figure 96.**
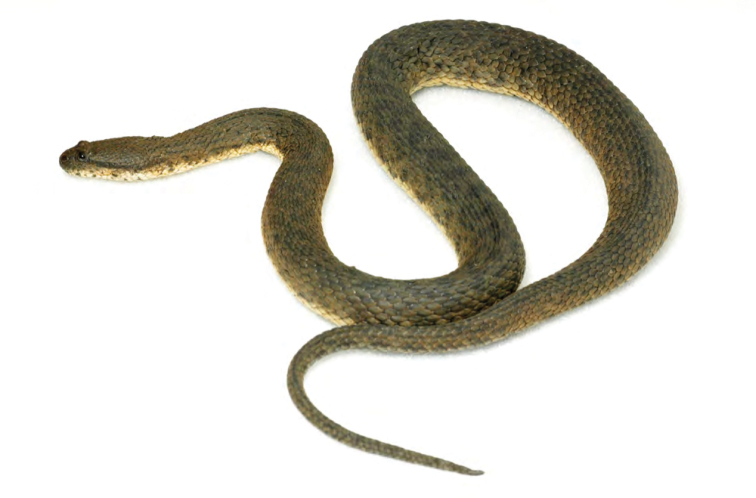
*Cerberus schneideri* (uncataloged ACD specimen in PNM). Photo: ACD.

Dog-faced water snakes (Fig. 96) are distributed in coastal areas throughout much of Southeast Asia (Gyi 1970). In a recent comprehensive systematic review, Murphy et al. (2012) identified species-level diagnostic differences corresponding to previously identified phylogenetic breaks (Alfaro et al. 2004, 2008), necessitating the elevation of a formerly synonymized name (*Cerberus schneideri)* to accommodatethe distinctive lineage distributed throughout the coasts of Malaysia, Indonesia and the Philippines. The taxon *Cerberus rynchops* is now restricted to the coasts of Thailand, Myannmar, the Indian subcontinent, and Sri Lanka. *Cerberus schneideri* has been documented on most major islands of the Philippines and our specimen originated in the Municipality of Santa Ana, along the northeast coast of Luzon.

Cagayan Province—Location 12: PNM 7544.

### Family Lamprophiidae

***Oxyrhabdium leporinum leporinum* (Günther, 1858)**

**Figure 97.**
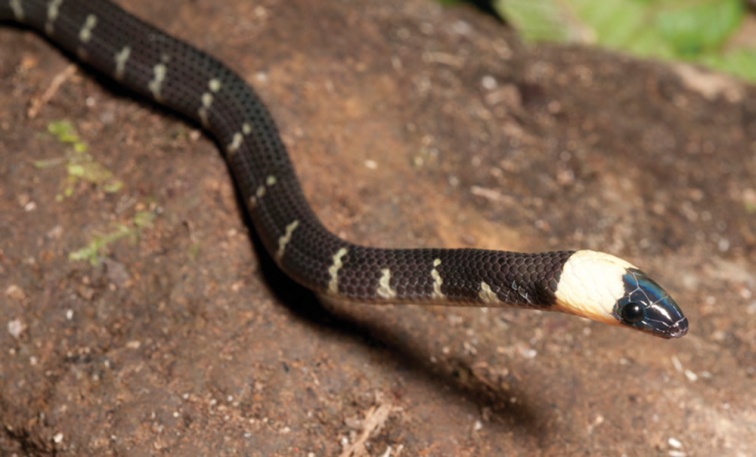
*Oxyrhabdium leporinum leporinum*(KU 330079)from mid-elevation, Mt. Cagua, Location 1b. Photo: RMB.

Adults of this common Luzon faunal region endemic (Fig. 97) are frequently encountered actively foraging along stream banks in forests of varying levels of disturbance (Leviton 1964c; Brown et al. 2000a, 2012; Diesmos et al. 2005; Siler et al. 2011); juveniles are most frequently found at night, sleeping perched in herbaceous layer vegetation, ferns, and small shrubs in riparian habitats (McLeod et al. 2011). Our specimens were found coiled on axils of ferns along a stream bank in selectively logged forest at 600+ masl.

Cagayan Province—Location 1b: KU 330079–80.

### Family Typhlopidae

***Ramphotyphlops braminus* (Daudin, 1803)**

Specimens of this common, parthenogenetic, presumably introduced species were collected under rocks, palm fronds, and other debris on the edge of forests, and within selectively logged forests at low- to mid-elevations.

Cagayan Province—Location 15: USNM 498933–48, PNM 5476–80; Location 16: USNM 498932.

Isabela Province—Location 24: KU 326636, 326639; Location 25: KU 326640.

***Typhlops ruficaudus* (Gray, 1845)**

A single specimen of this species was collected in a fern axil in a small primary growth forest fragment. As the taxonomy of Philippine typhlopids has improved in the past decade (McDowell 1974; Wynn and Leviton 1993; McDiarmid et al. 1999) this species has emerged as a moderately common component of Luzon’s herpetofauna (McLeod et al. 2011).

Isabela Province—Location 28: KU 328598.

***Typhlops* sp. 1**

This distinctive, probable new species appears related to the *Typhlops ruficaudus* Group (Wynn and Leviton 1993), but differs from other members of that group on the basis of several characters of scalation and body size.

Isabela Province—Location 23: KU 328594.

***Typhlops* sp. 2**

This distinctive, probable new species appears phenotypically most similar to *Typhlops luzonensis*, but differs from that species on the basis of several characters of scalation and body size.

Cagayan Province—Location3: KU 328597.

### Family Viperidae

***Trimeresurus flavomaculatus* (Gray, 1842)**

**Figure 98.**
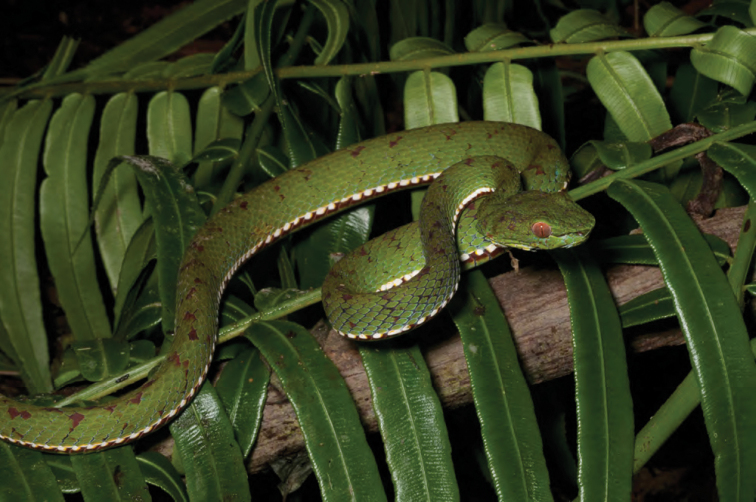
*Trimeresurus flavomaculatus* (KU 330049)from mid-elevation, Mt. Cagua, Location 1b. Photo: RMB.

Exceedingly common on the lower slopes of Mt. Cagua, *Typhlops flavomaculatus* (Fig. 98) was observed in high densities surrounding ephemeral pools during the start of the rainy season at mid-elevations (400–700 m). We encountered multiple individuals per night at the same temporary pond as they actively hunted frogs (*Occidozyga laevis*, *Polypedates leucomystax*, *Rhacophorus pardalis*, *Kaloula rigida* and *Kaloula picta*) in the lower strata (30–100 cm above the forest floor) of shrub layer vegetation or in temporary pools along muddy paths in secondary forest. *Trimereserus* *flavomaculatus* is widespread and common throughout the Luzon faunal region (Leviton 1964a; Brown et al. 1996, 2000a, 2012; Diesmos et al. 2005; Siler et al. 2011; McLeod et al. 2011; Devan-Song and Brown 2012).

Cagayan Province—Location 1a: KU 330038–41; Location 1b: KU 330042–53.

Isabela Province—Location 23: KU 327223–24; Location 26: KU 327225–27; Location 32: no specimens (MVW photo voucher).

***Tropidolaemus subannulatus* (Gray, 1842)**

This common and widespread Luzon and Visayan region pit viper is frequently encountered in forested areas from sea level to mid-montane elevations. Although no specimens are available, it has been photographed in the Dibanti River area, Municipality of San Mariano.

Isabela Province—Location 26: no specimens (ACD photo voucher).

### Reptilia: Turtles. Family Geoemydidae

***Cuora amboinensisamboinensis* (Daudin, 1802)**

**Figure 99.**
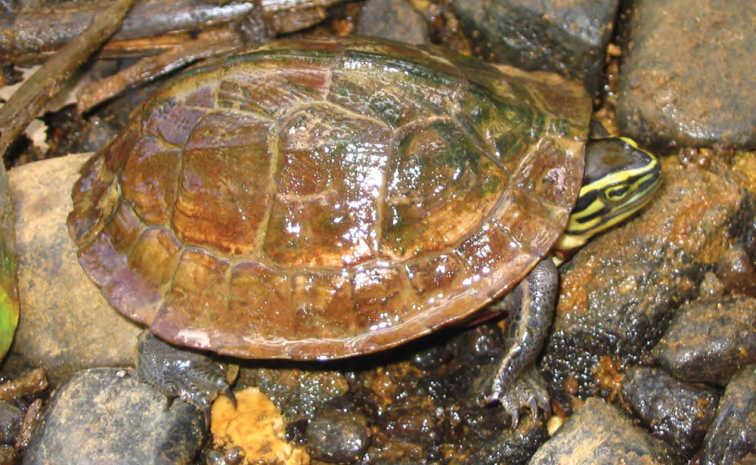
*Cuora amboinensis amboinensis* (ACD 3229, deposited in PNM) from Barangay Binatug (Location 22). Photo: K. M. Hesed.

Diesmos et al. (2008) documented the presence of this species (Fig. 99) at several sites in Cagayan and Isabela Province. At the Municipality of Baggao, local residents in the vicinity of hot springs collected this species along the Intal River. Additional Isabela Province specimens were collected along irrigation canals in flooded rice fields near disturbed secondary growth forest.

Cagayan Province—Location 15: USNM 498949, 499260–61; Location 19: uncataloged specimen in PNM.

Isabela Province—Location 21: PNM 6730; Location 29: PNM 658; Location 30: PNM 6499.

### Family Trionychidae

***Pelochelys cantorii* Gray, 1864**

**Figure 100.**
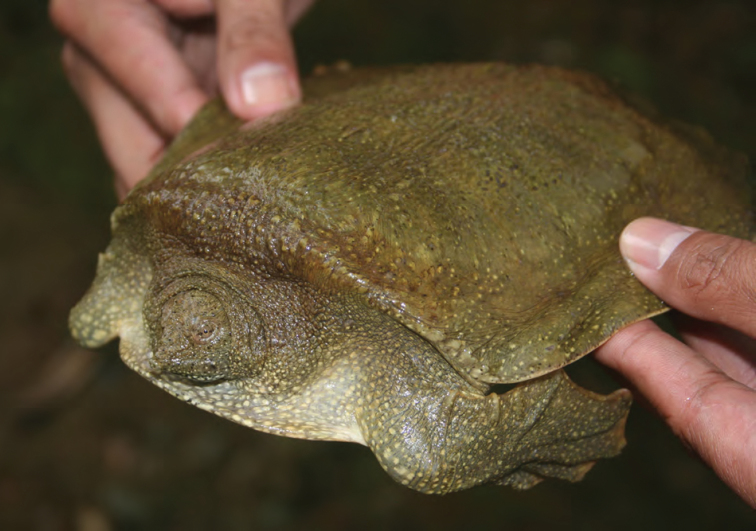
*Pelochelys cantorii* from the vicinity of San Mariano (specimen not collected); Photo: MVW.

**Figure 101.**
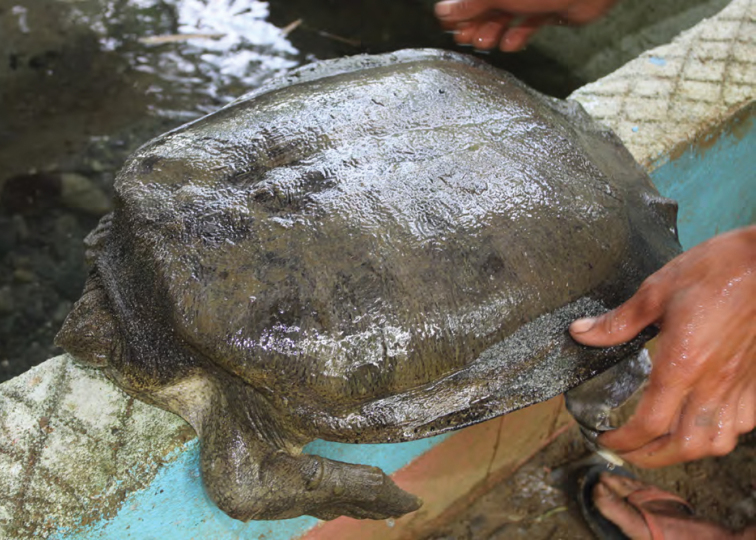
*Pelochelys cantorii* from the vicinity of St Victoria (specimen not collected); Photo: MVW.

Diesmos et al. (2008) documented the presence of this species (Figs 100, 101) at several sites in Cagayan and Isabela Province. The taxonomic status of Philippine populations of *Pelochelys cantorii* requires re-evaluation. As currently understood, *Pelochelys cantorii* occurs from southern India through Bangladesh, southern China, Myanmar, Vietnam, Cambodia, Laos, Thailand, Malaysia, and southern Borneo, Indonesia (Lim and Das 1999; Ernst et al. 2000; Webb 1995, 2002; Stuart and Platt 2004; Fritz and Havas 2007). We consider this widespread distribution unlikely for a single species. Morphological variation in skull and carapace morphology (neural bone counts) has been reported between Philippine and mainland Asian populations of *Pelochelys cantorii* (Baur 1891; Taylor 1920b, 1921), suggesting that Philippine populations may be distinct. If so, Gray’s (1864) epithet (*Pelochelys cumingii*) would be the appropriate name for this possible Philippine endemic.

Cagayan Province—Location 3: ACD field observation (June, 2006; no specimen); “Upper Cagayan River Basin:” PNM 8487; Location 31: no specimens (ACD field observation).

Isabela Province—Location 24 and St. Victoria: no specimens (MVW photo voucher).

### Family Cheloniidae

***Caretta caretta* (Linnaaeus, 1758)**

Loggerhead Turtles have been observed in coastal waters of Isabela Province (van Lavieren et al. 2009) but studies of their habitats and natural history along the east coast of Luzon are lacking.

***Chelonia mydas* (Linnaaeus, 1758)**

Green Turtles have been observed nesting on the beaches of the coast of Isabela Province (MVW, *personal observations*); we expect the vast, undeveloped coastline of Cagayan Province also supports nesting populations to the north.

Isabela Province—Location 30: no specimens (MVW, *personal observations*).

***Eretmochelys imbricata* (Linnaeus, 1766)**

Hawksbill Turtles nest on the beaches of the coast of Isabela Province (MVW, *personal observations*) and we expect this species also nests along the beaches of Cagayan Province to the north.

Isabela Province—Location 30: no specimens (MVW, *personal observations*).

### Reptilia: Crocodiles. Family Crocodylidae

***Crocodylus mindorensis* Schmidt, 1935**

**Figure 102.**
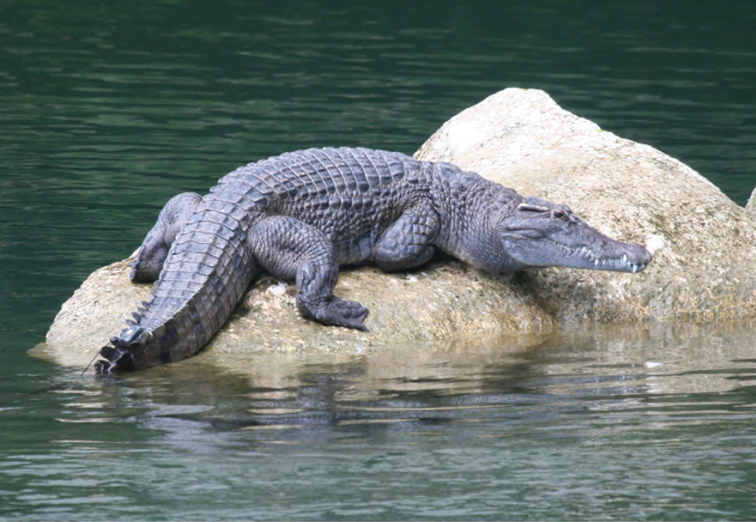
*Crocodylus mindorensis* basking on a rock in the Disulap River, Barangay Disulap (Location 29): Photo MVW.

**Figure 103.**
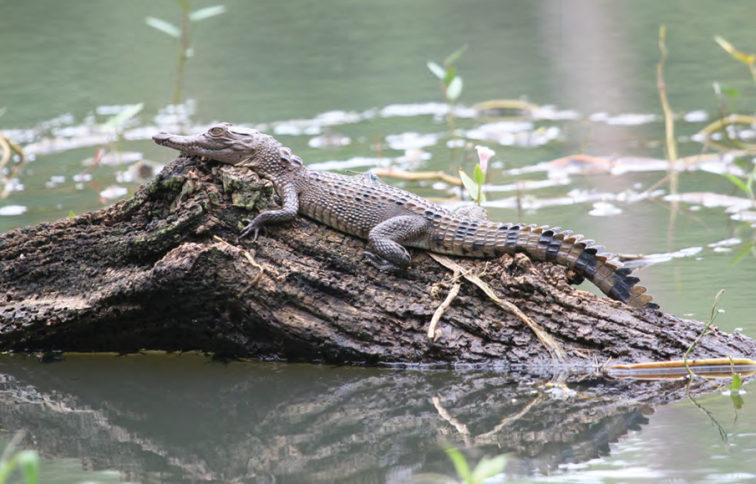
*Crocodylus mindorensis* basking on a log in the Dunoy Lake, Barangay Dibuluan (Location 24): Photo MVW.

The target of an extensive local conservation program (van Weerd and van der Ploeg 2004a,b; van der Ploeg and van Weerd 2004; van Weerd 2010; van der Ploeg et al. 2011a, 2011b, 2011c), *Crocodylus mindorensis* (Figs 102, 103) is now known from the municipalities of Maconacon, Divilacan, Palanan, San Mariano, Benito Soliven and San Guillermo in Isabela Province. Tolerant of substantial habitat disturbance and capable of living in close proximity with humans, remaining populations of this species are most severely threatened by continued habitat loss and degradation, and persecution by people. The USNM specimens were purchased from a dealer and reportedly came from the northern part of the Cagayan Valley (exact locality unknown). Recent records include field observations of live *Crocodylus mindorensis* animals in riverine and coastal habitats, including under saline conditions, in the municipalities of Maconacon, Divilacan and Palanan along the Pacific coast of Isabela and in inland freshwater rivers, creeks and small ponds in San Mariano, Benito Soliven and San Guillermo in Isabela Province. There are, as yet, unconfirmed reports of *Crocodylus mindorensis* in the municipality of Baggao in Cagayan, and the species occurred with certainty until recently in small lakes and rivers in Peñablanca, also in Cagayan Province. There are, furthermore, unconfirmed reports of *Crocodylus mindorensis* from the tributaries to the Cagayan River that originate in the Cordillera Mountains along the western edge of Cagayan Valley, in both Isabela and Cagayan Provinces.

Cagayan Province—Location: “Northern Cagayan Valley:” USNM 252669, 252699, 252670.

Isabela Province—Locations 23, 28, 34, 36, Divilacan, Benito Soliven and San Guillermo: No specimens (MVW *personal observations* and photo vouchers).

***Crocodylus porosus* Schneider, 1801**

**Figure 104.**
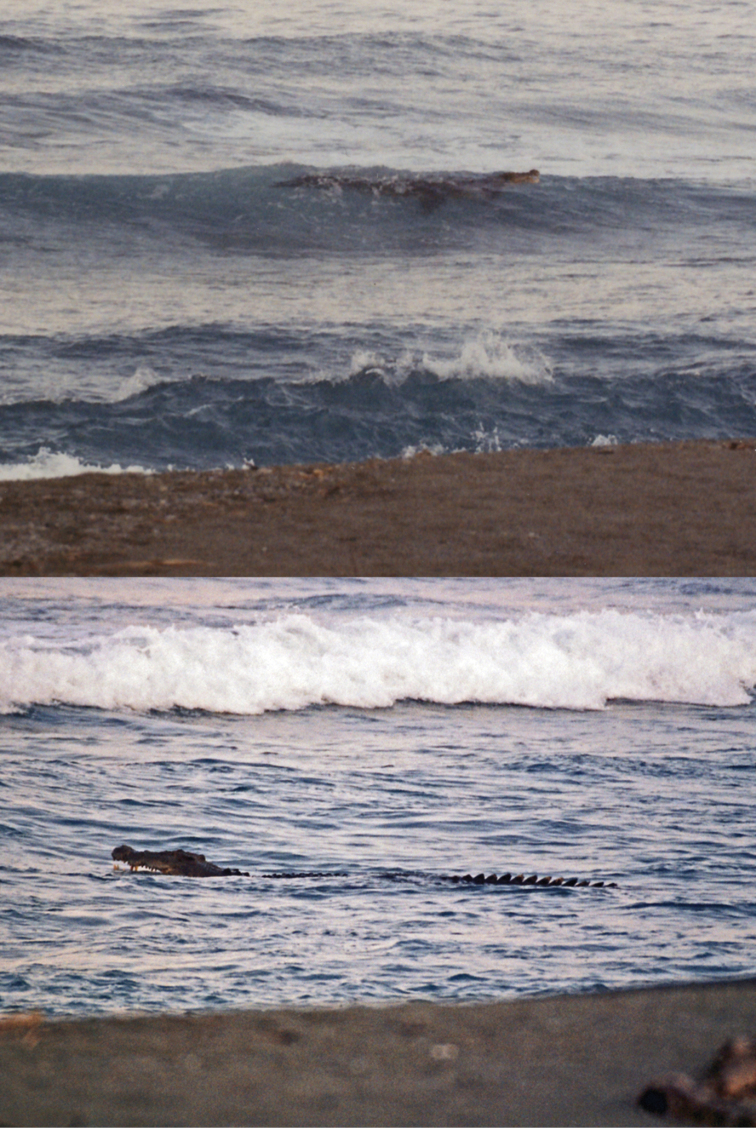
*Crocodylus porosus* foraging in surf at Maconacon, March 2004. Photo MVW.

The large-bodied saltwater crocodile *Crocodylus porosus* is distributed in estuarine and coastal areas, swamps and rivers, from India, throughout Southeast Asia, eastward to Australia (Iskandar 2000). Recent records include documented field observations of *Crocodylus porosus* in coastal areas in the Municipalities of Maconacon and Palanan, Isabela Province (Fig. 104). On 17 March 2004, from the beach near the village of Reina Mercedes in Maconacon, MVW observed and photographed a 3.5 m long saltwater crocodile floating and diving c. 100 m out at sea. At night it crossed a sandbank and entered a tidal marsh area next to the village where fresh tracks were found the following day. On 27 March 2004, MVW observed and filmed a c. 3 m-long basking saltwater crocodile in a mangrove swamp near the village of Culasi in Palanan. Spanish geographers describe the presence of large numbers of large crocodiles, presumably *Crocodylus porosus*, basking on the banks of Cagayan River in the 17^th^-19^th^ century. Saltwater crocodiles have now been extirpated from Cagayan River itself but continue to survive in small numbers along the sparsely inhabited eastern coast of northeastern Luzon.

Isabela Province—Locations 34, 36: No specimens (MVW *personal observations* and photo vouchers).

## Discussion

Recent studies focusing on the major subcenters of herpetological endemism of the Luzon faunal region (Brown and Diesmos 2002, 2009) have documented between 52 to 85 species for a given area (Ross and Gonzales 1992; Brown et al. 1996, 2000a, 2012; *unpublished data*; Diesmos et al. 2005; Oliveros et al. 2011; Siler et al. 2011a; McLeod et al. 2011; Devan-Song and Brown 2012). As reviewed by Auffenberg (1988; see also Hall 1998, 2001, 2002; Yumul et al. 2003, 2009), the occurrence of multiple geological components of central and southern Luzon correspond in some cases to approximate paleoisland precursors that accreted in recent geological history (mid- to late Cenozoic) to form today’s geographically complex, tectonically active southern portion of Luzon (Hall 2002; Yumul et al. 2003). In contrast, northern Luzon, at the northern end of the Philippine Sea Plate and contained within east-dipping Manila Trench and the west-dipping proto-East Luzon Trough (Florendo 1994; Hall 2002; Yumul et al. 2003) is characterized by two main montane components: the Cordillera and the Sierra Madre. Because of the intriguing possibility of a history of isolation on past paleoisland microterranes or ancient montane formations, biogeographers have often noted the occurrence of regional faunas that appear to coincide with the major geological components of the island (Auffenberg 1998; Brown et al. 1996; Diesmos et al. 2005; Siler et al. 2011a; Welton et al. 2010, 2012). Thus, our current expectation is that some degree of local faunal endemism should be discernable and uniquely associated with the Cordilleras, the Sierra Madre, the Zambales, the volcanoes of southern Luzon, and the Bicol Peninsula (and associated islands of Polillo and Catanduanes; Brown et al. 1996, 2000a, 2012; Ross and Gonzales 1992; Diesmos et al. 2005; Devan-Song and Brown 2012).

This study further supports our general expectation of within-Luzon biogeographic provincialism in the sense that the northern Sierra Madre, like the other major components of Luzon, contains a high percentage of locally endemic amphibians and reptiles. At the same time, this study confirms recent and unexpected findings of a considerable degree of overlap in faunal elements between the northern Cordilleras and the northern Sierra Madre (Diesmos et al. 2005; Brown et al. 2012).

In this study we have made a preliminary, first-pass, enumeration of the amphibians and reptiles of the northern Sierra Madre and documented species diversity of this region at more than 100 species. This level of diversity surpasses all but one faunistic study for Luzon (Siler et al. 2011a) and rivals other regional high-diversity areas throughout the archipelago. Only the cumulative results of the central Mindanao surveys of Taylor (based on several years of work in Bunawan, Agusan del Norte, Mindanao Island; Taylor 1920a, b, 1921, 1922a, b, c, d, 1923), the eastern Mindanao surveys of Brown and Alcala (*unpublished data*, available at California Academy of Sciences Herpetological database web portal), and new, repeated western Mindanao surveys of Brown et al. (*unpublished data*, available at the University of Kansas Specify database web portal) include species diversity estimates upwards of 80–90 species for a given site.

In a 10-day preliminary survey at Aurora National Park, Brown et al. (2000a), used species accumulation curves to demonstrate that even substantial sampling efforts (involving well trained field teams with 4–6 workers) will underestimate diversity—as measured by species accumulation curves that failed to asymptote during their intensive, but brief, field survey effort. In a more recent study a decade later, Siler et al. (2011a) increased Aurora diversity to 85 species by visiting different drainages in the same park, focusing on different habitat types, sampling a wider range of elevations, and concentrating survey efforts across a broader range of atmospheric conditions. The lessons from the combinations of these two studies, as well as two studies from the northern Cordilleras (Diesmos et al. 2005; Brown et al. 2012) are very clear—and provide important “best practice” guidelines for future biodiversity inventories and conservation efforts in this and other similar archipelagos.

We concentrate on these two pairs of studies because they are the only two of their kind for Luzon: the northern Cordillera surveys (Diesmos et al. 2005; Brown et al. 2012) and the repeat assessments at Aurora National Park (Brown et al. 2000a; Siler et al. 2011a). Together, these studies indicate that no site can be reasonably characterized for resident biodiversity with a single visit, even if a considerable effort is exerted over a multi-week field expedition (typical of the last several decades of field surveys throughout the Philippines; *personal communications* with A. C. Alcala, D. S. Rabor, L. A. Heaney, R. S. Kennedy, P. C. Gonzales, and colleagues). Because of annual herpetological species activity patterns associated with seasonality and the reproductive effort (Taylor 1917; Brown and Alcala 1963, 1981, 1986; Auffenberg and Auffenberg 1988, 1989; review: Brown et al. 2002; Gaulke 2011), it is imperative that expeditionary inventory fieldwork focuses on a given area for minimum of one survey in the dry season and a follow-up effort in the rainy (“monsoon”) season. Our recent efforts have attempted to maximize atmospheric variation (principally, variation in occurrence and severity of precipitation events) by concentrating survey work at the beginning of the rainy season (June–August) in hopes of sampling the same sites when dry and, subsequently, following the first heavy rains. However, even these sequential efforts targeting major shifts in habitat and climactic variables fail to capture annual variation in the reproductive effort (and thus, detectability of a given species) in the majority of species present (Alcala, 1967; Alcala and Brown 1967, 1982; Brown and Alcala 1970b, 1982; Alcala and Alcala 1980; Auffenberg 1988; Auffenberg and Auffenberg 1988, 1989; Gaulke 2011).

A second major lesson involves the naturally patchy distribution of many amphibian and reptile species (Brown and Alcala 1963, 1981, 1986). The experience drawn from the Siler et al. (2011a) follow up to the Brown et al. (2000a) Aurora surveys indicate the need for concentrated survey efforts across many sub-sites (habitat types) within a general area. The Siler et al. (2011a) effort nearly doubled the known herpetological diversity of Aurora National Park by focusing on south versus north facing slopes, different streams and river drainages, geological variables (karst versus volcanic soils), variation in elevational gradients, and different forest types/plant communities. Most of Siler et al.’s (2011a) sampling locations were within kilometers of Brown et al.’s (2000a) work; the critical difference, in our opinion, was the simple fact that Siler et al. detected additional species by sampling a broader range of habitat heterogeneity and climactic variability. Because of our collective experience with the last 15 years of survey work on Luzon, we are now compelled to be extremely cautious about generalizing conclusions regarding patterns of biodiversity, abundance, biogeography, endemism, and especially conservation status from any single-visit faunal inventories. As Brown et al. (2012) emphasized in a recent study of Ilocos Norte Province (northwest Luzon), arid conditions associated with the dry season render conclusions about amphibian communities derived from surveys during these times moot for the fundamental reason that negative data are uninformative for the purposes of determining species presences/absences. The same is clearly true for any single-visit survey to a given area: the apparent absence of a species (non-detection or negative data) during a survey actually tells us nothing about the abundance of that taxon, its distribution or, most significantly, its conservation status (Brown et al. 2012).

With these caveats in mind, diversity patterns for the reasonably well surveyed areas of Luzon include 52–55 species for the Northern Cordillera (Diesmos et al. 2005; Brown et al. 2012), 63 species for Bulacan Province, southern Sierra Madre (McLeod et al. 2011), 52–60 species from the Zambales Mountains (Brown et al. 1996; Devan-Song and Brown 2012), 52 species from the Babuyan Island Group, north of Luzon (Oliveros et al. 2011), 58 species for Catanduanes Island (Ross and Gonzales 1992), 52–56 species from the coastal forests of Subic Bay (Devan-Song and Brown 2012), and 85 species from the central Sierra Madre (Brown et al. 2000a; Siler et al. 2011a). A number of species are conspicuously absent from our total estimates; many of these being common throughout coastal areas of Luzon (Inger 1954; Leviton 1962, 1963a, b, 1964a, b, 1965a, b, 1967, 1970; Brown and Alcala 1974; 1980; Alcala and Brown 1998), but we predict that these species will be recorded during future surveys in the northern Sierra Madre. These species include widespread endemic amphibians like *Rhacophorus bimaculatus*, the introduced species *Hylarana erythraea* (Diesmos et al. 2006), and several undescribed *Platymantis* species now known from Aurora Province to the south of Isabela (Brown et al. 2000a; Siler et al. 2011a). Lizard species that we expect will eventually be recorded from the northern Sierra Madre include *Gonocephalus sophiae*, *Hydrosaurus pustulatus*, *Luperosaurus angliit* (Brown et al. 2011),*Brachymeles boulengeri*, *Brachymeles elerae* (Siler, 2010), *Dasia grisea*, *Emoia atrocostata*, *Eutropis bontocensis*, *Parvoscincus luzonensis*, *Parvoscincus lawtoni*, and *Parvoscincus igorotorum* (Brown et al. 2010b). Snakes that we expect to be residents in the northern Sierra Madre include *Boiga angulata*, *Chrysopelea paradisi*, *Pseudorhabdium oxycephalum*, *Rhabdophis baurbori*, *Myersophis alpestris* (Taylor 1963; Leviton 1983),* Acutotyphlops banaorum* (Wallach et al. 2007), *Typhlops luzonensis*, *Typhlops ruber*, and *Typhlops cumingi* (McDowell 1974; McDiarmid et al. 1999). Additionally, we anticipate that the introduced freshwater turtles *Pelodiscus sinensis* and *Chrysemys picta* (Diesmos et al. 2008) will eventually be encountered in the northern Sierra Madre. Coastal areas (with their potential habitats for sea snakes, marine turtles, rare forest geckos, and selected scincid lizards; Alcala, 1986; W. Brown et al. 1978, 1980; R. Brown et al. 2007, 2011) are likely to support additional species diversity.

At present the Luzon faunal region’s herpetological diversity stands at more than 150 species. A total of 49 total amphibian species have been documented, 44 of which are native (5 introduced; Diesmos et al. 2006), and 32 of which are endemic (Brown and Alcala 1970a, 1994; Brown 2007; Brown et al. 2008; Brown and Diesmos 2009; Diesmos and Brown 2011). Luzon supports at least 106 native reptiles, 76 of which are endemic to this faunal region (Leviton 1963a; Brown and Alcala 1978, 1980; Diesmos et al. 2002). If the percentage of species associated with unresolved taxonomic problems identified here (~38%) can be extrapolated to the total fauna, as many as 20 of Luzon’s current amphibian species and as many as 40 current reptile taxa may be associated with future taxonomic changes. The majority of these will most likely involve partitioning of species complexes into two or more distinct evolutionary units (Ron and Brown 2008; Bain et al. 2008; Brown et al. 2008; Brown and Stuart 2012).

In addition to this expected increase in biodiversity associated with refined taxonomic partitioning (e.g., splitting) of species groups, the northern Philippines has been the focus of the majority of *de novo* new species discovery in recent decades (W. Brown et al. 1997a, b, c; 1999a, b; Alcala et al. 1998; Alcala and Brown 1998, 1999; R. Brown et al. 1999, 2000b, 2007, 2011; Siler 2010; Siler and Brown 2010; Welton et al. 2010; Diesmos and Brown 2011). Our estimates place the remaining known new taxa (i.e., already in collections, represented by specimens clearly identified as new species) awaiting description in the Luzon faunal region at approximately 25 amphibian and 15 reptile species (RMB, A. C. Alcala, ACD, and CDS, *unpublished data*). When the two sources of undescribed herpetological diversity for Luzon are combined, a striking potentiality for unknown diversity emerges: we anticipate that the diversity of the island may grow to as many as 90–100 (70–80% endemic) amphibian species and as many as 150–160 reptile species with ongoing biodiversity studies in the near future. Clearly the herpetological biodiversity of the northern Philippines is substantially underestimated.

The results of this and related studies from the northern Philippines contribute to an ongoing revision of the biogeographic characterization of the Philippines as a “fringing archipelago” with a depauperate fauna in its northern regions (Dickerson 1928; Taylor 1928; Darlington 1957; Myers 1960, 1962; Carlquist 1965; Leviton 1963a; Brown and Alcala 1970a). As more recent studies have focused on *in situ* diversification within the archipelago (Heaney 2000; Brown and Guttman 2002; Evans et al. 2003; Heaney et al. 2005; Jansa et al. 2006; Linkem et al. 2010; Siler et al. 2010c, 2011b, 2012; Siler and Brown 2011; Esselstyn et al. 2009; Esselstyn and Brown 2009), the northern Philippines has emerged as a major regional hotspot for the autochthonousproduction of vertebrate biodiversity via a variety of evolutionary processes of diversification (Brown and Diesmos 2009).

Conservation of Luzon’s vertebrate biodiversity—in particular the more spectacular Philippine evolutionary radiations and complex ecological communities supported by the remaining forested areas of Luzon—remains an on-going effort, challenged by rapid development, large-scale extractive logging and mining industries and conversion of natural habitats into agricultural lands driven by a burgeoning human population (Liu et al. 1993; Uitamo 1999; van der Ploeg et al. 2011d). A suite of recent studies has shown that some forested regions closest to the country’s large major metropolitan areas remain among the least studied of Luzon’s forests (Diesmos 1998; Diesmos and Brown 2011; McLeod et al. 2011; Devan-Song and Brown 2012); these areas are immediate priorities for comprehensive faunal surveys of the type presented here. In contrast, forested areas that have been properly surveyed for herpetological diversity rank among the areas supporting the country’s most diverse herpetological communities (Brown et al. 2000a, 2012; Siler et al. 2011a). Before reasonably well-informed, biologically meaningful conservation measures are to be effective, a basic understanding of distribution patterns and cross-taxon congruence of Luzon’s vertebrate biodiversity will be necessary (Brown and Diesmos 2009; van Weerd and Udo de Haes 2010; Diesmos and Brown 2011). In the absence of actual, field-based, empirical, survey data, conservation status assessments (IUCN 2010) and priority setting exercises (Diesmos et al. 2002; Diesmos and Brown 2011) will remain incomplete, uninformed, and overly reliant on secondary sources, extrapolation, and “expert” opinion (Diesmos et al. 2004; Brown et al. 2012). In the northern Sierra Madre where, despite the fact that large areas are protected on paper, threats to the remaining large tracts of forested areas have been clearly identified (NORDECO 1988; Hicks 2000; van Weerd and Udo de Haes 2010; van der Ploeg et al. 2011d; Minter et al. 2012), and a major challenge will be to monitor herpetological communities through time in order to assess communities’ responses to land use changes, climate change, resource extraction, introduced species, emerging infectious disease, and habitat degradation. With the initial baseline information provided here, tremendous opportunities exist for future studies in taxonomy, biogeography, ecology and conservation of northern Luzon’s amphibians and reptiles.
